# Climate vulnerability assessment of key fishery resources in the Northern Humboldt Current System

**DOI:** 10.1038/s41598-022-08818-5

**Published:** 2022-03-21

**Authors:** Jorge E. Ramos, Jorge Tam, Víctor Aramayo, Felipe A. Briceño, Ricardo Bandin, Betsy Buitron, Antonio Cuba, Ernesto Fernandez, Jorge Flores-Valiente, Emperatriz Gomez, Hans J. Jara, Miguel Ñiquen, Jesús Rujel, Carlos M. Salazar, Maria Sanjinez, Rafael I. León, Mark Nelson, Dimitri Gutiérrez, Gretta T. Pecl

**Affiliations:** 1 Institute for Marine and Antarctic Studies, University of Tasmania, Hobart, TAS Australia; 2 Falkland Islands Fisheries Department, Directorate of Natural Resources, Stanley, Falkland Islands; 3 Instituto del Mar del Perú, Callao, Lima, Peru; 4 Laboratorio de Ciencias del Mar, Facultad de Ciencias Y Filosofía, Centro de Investigación Para El Desarrollo Integral Y Sostenible (CIDIS), Universidad Peruana Cayetano Heredia, Lima, Peru; 5 Facultad de Ciencias Biológicas, Universidad Nacional Mayor de San Marcos, Lima, Peru; 6 Laboratorio de Ecofisiología de Crustáceos, Instituto de Acuicultura, Universidad Austral de Chile, Puerto Montt, Chile; 7 Sociedad Peruana de Derecho Ambiental, San Isidro, Lima, Peru; 8 ECS, in Support of NOAA Fisheries, Office of Science and Technology, Silver Spring, MD USA; 9 Centre for Marine Socioecology, University of Tasmania, Hobart, TAS Australia

**Keywords:** Marine biology, Climate change

## Abstract

The Northern Humboldt Current System sustains one of the most productive fisheries in the world. However, climate change is anticipated to negatively affect fish production in this region over the next few decades, and detailed analyses for many fishery resources are unavailable. We implemented a trait-based Climate Vulnerability Assessment based on expert elicitation to estimate the relative vulnerability of 28 fishery resources (benthic, demersal, and pelagic) to the impacts of climate change by 2055; ten exposure factors (e.g., temperature, salinity, pH, chlorophyll) and 13 sensitivity attributes (biological and population-level traits) were used. Nearly 36% of the species assessed had *“high”* or *“very high”* vulnerability. Benthic species were ranked the most vulnerable (gastropod and bivalve species). The pelagic group was the second most vulnerable; the Pacific chub mackerel and the yellowfin tuna were amongst the most vulnerable pelagic species. The demersal group had the relatively lowest vulnerability. This study allowed identification of vulnerable fishery resources, research and monitoring priorities, and identification of the key exposure factors and sensitivity attributes which are driving that vulnerability. Our findings can help fishery managers incorporate climate change into harvest level and allocation decisions, and assist stakeholders plan for and adapt to a changing future.

## Introduction

**Figure 1:**
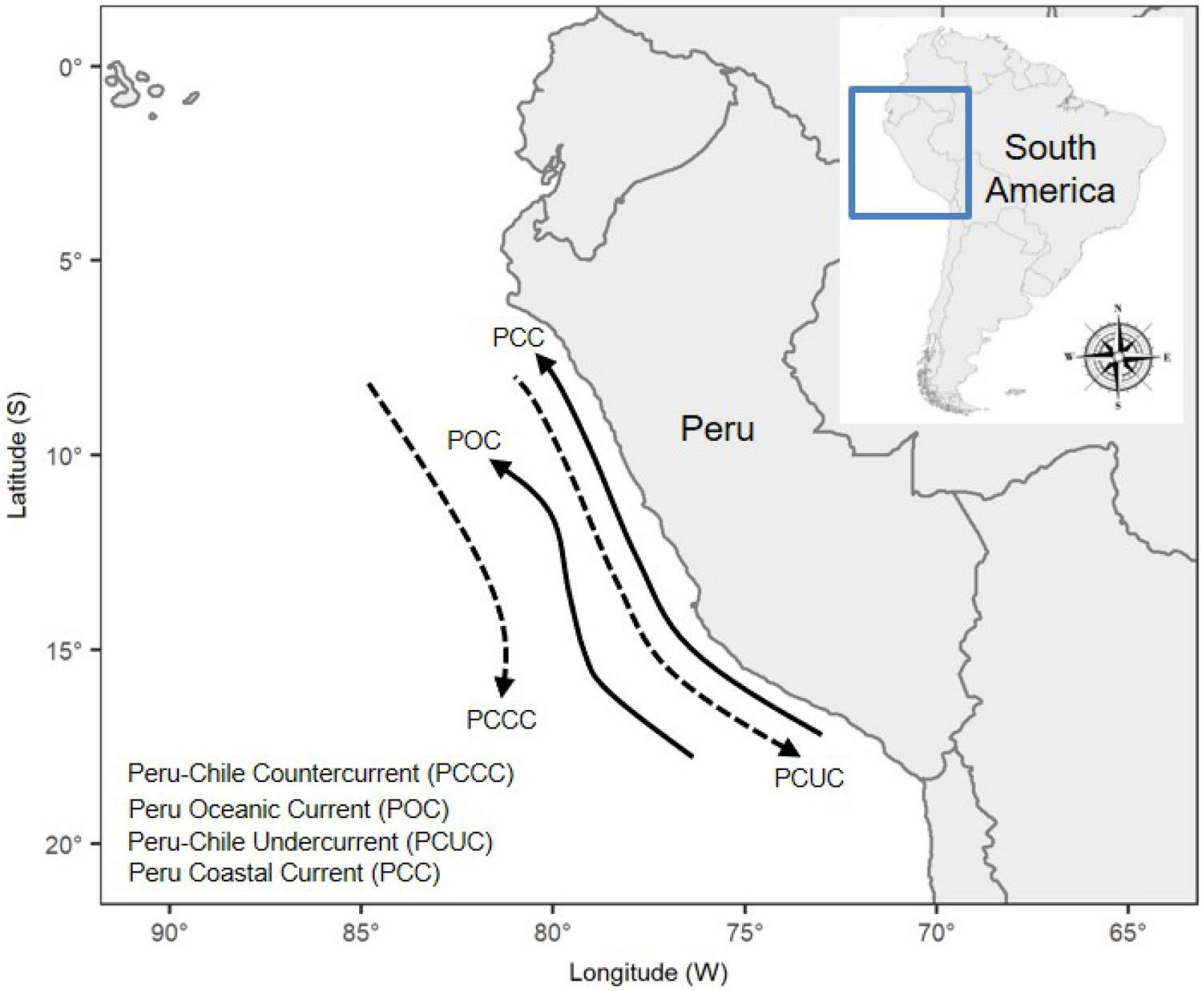
Peruvian portion of the Northern Humboldt Current System. Sub-surface (dashed line) and surface (solid line) currents are not to scale. The Peru–Chile Countercurrent flows southward and veers to the west at around 15°S. The Peru Oceanic Current flows equatorward at > 150 km offshore. The Peru–Chile Undercurrent flows poleward along the outer continental shelf and inner slope. The Peru Coastal Current flows equatorward and close to the coast^1–3^.

The Northern Humboldt Current System (NHCS) is part of the Humboldt Current System in the Southeastern Pacific^1–3^ (Fig. 1). The NHCS is characterised by high rates of upwelling and flux of nutrients^4^ that sustain high abundances of marine resources off Peru. In 2018, Peru was ranked the second most important marine fisheries producer in the world, contributing nearly 8% of the world’s total catch, and leading the production and export of fishmeal and fish oil^5^. Finfish, molluscs, and crustaceans comprise approximately 90%, 8%, and 1% of Peruvian industrial fisheries, respectively^6^. From 2009 to 2013 about 6 million tons were landed annually, and these were mainly composed of the Peruvian anchovy (84%) and the jumbo flying squid (7%)^7^. In 2020, all Peruvian anchovy landings were from the north and central zones of Peru^8^. Peru has a moderate to high dependency on fish and fisheries^9, 10^, with marine and inland Peruvian fisheries employing approximately 99,000 people, and the value of fisheries exports reaching USD$2.736 billion in 2017 alone^11^. Climate change is expected to alter significantly the marine environments of Peru over the coming decades. Oceanic circulation along the coast of Peru will change due to modifications in surface winds and increased stratification, with a reduction in wind stress and decrease in upwelling intensity^12^. The surface layer within 100 km from the central coast of Peru is projected to warm up to 4.5 °C by 2100; the 20 °C isotherm (as a proxy for the thermocline) may be deepening at ~ 1.5 m/decade by 2065, and by ~ 5 m/decade between 2065 and 2100^13^. Sea-level rise is projected to increase 1.2–3.4 mm/year over the same period and mainly towards the south, and greater salinity increases are expected from the central area off Peru towards the north, with up to 1.25 PSU increase by 2070^14^.

The impacts of climate change on marine species and the fisheries they support are anticipated to accelerate^15, 16^ as climate change intensifies over the coming decades^17, 18^. Changes in species distribution are one of the most documented responses to climate change as species track favourable temperatures, generally shifting poleward or to deeper waters^19–21^. Decrease in abundance of cold-water species and increase in abundance of warm-water species are expected with climate-driven oceanic warming^16^. However, tropical ectothermic species have higher upper thermal limits compared with temperate ectothermic species^22^. Hence, tropical ectothermic species that live close to their thermal limits and that have limited dispersal or movement capacity may not be able to track favourable temperatures, and their abundances would also be negatively affected via declines in recruitment or breeding success^15, 16^. As a consequence, species that are traditionally caught in some areas may decrease in abundance or disappear, whereas species that are not traditionally caught may increase in abundance. Moreover, the decoupling of phenological events across functional groups and trophic levels will result in changes to trophic interactions and food webs, with consequences for the structure and function of marine ecosystems^23^.

Changes in distribution, abundance and phenology can affect the location and timing of fishing areas, duration of navigation and fuel consumption, and composition and abundance of catches^24^. Such modifications in fish-species composition and reductions in fisheries production associated with climate change will increase the vulnerability of countries whose adaptive capacity is limited^24, 25^. The effects of interannual and decadal variability have been evident on the NHCS marine life^26–28^, and marine species in the Peruvian upwelling ecosystem seem vulnerable to high temperatures associated with El Niño^29^. Environmental variability in the region associated with climate change may exacerbate environmental effects on NHCS marine resources and the economic activities that depend on them. Hence, Peru has been ranked the 10^th^ most vulnerable economy to marine climate change^9^. Maximum catch potential of commercial marine species is expected to decrease in different Exclusive Economic Zones (EEZs) due to climate change, and greater impacts are anticipated in tropical EEZs driven by oceanic warming, acidification, deoxygenation, and sea-level rise. In particular, maximum marine catch potential is projected to decline 20–50% in Peruvian latitudes by the year 2050 relative to the year 2000 under the Representative Concentration Pathway 8.5 (RCP 8.5)^25, 30, 31^.

The use of vulnerability assessments methodologies in the planning and scoping of management is of particular interest to identify climate change-related priority issues that affect the operability and sustainability of a fishery^32^. Different approaches can be used to assess the vulnerability of species to climate change, including correlative, mechanistic, and trait-based approaches^33, 34^. Correlative assessments are based on models that describe the correlation between the species' distribution and the contemporary climate, which can be used to identify the potential geographic distribution of the species under future climate conditions. Mechanistic assessments rely on models that project future ranges of species (mechanistic niche) from their physiological tolerances, which are usually estimated from field or laboratory observations, or from energy balance equations; or project changes in abundance (demographic models) usually by simulating climate change impacts on individuals, subpopulations, or species. The trait-based approach makes use of a range of biological and life history information to estimate species' relative vulnerability to climate change impacts^33–35^. Correlative and mechanistic approaches are very data intensive, whereas trait-based approaches are less resource-intensive; all three approaches have some level of uncertainty. Therefore, when data and resources are not a limitation, the three approaches may be combined to provide more robust vulnerability assessments^33, 34^.

The trait-based approach is useful to help non-GIS experts develop regional assessments and to identify conservation priorities in the absence of specific data on species’ distribution^33^. The trait-based approach is relatively rapid to perform, it accounts for the effect of species characteristics, and can be used to assess large numbers of species^34^. This approach is easy to understand, transferable, transparent, repeatable, scientifically defensible, precautionary, and useful for management^35, 36^. As such, various adaptations of the method have been implemented in Southeast Australia^35^, the Northeast U.S. shelf^37^, the southern Benguela system^38^, and the Eastern Bering Sea Shelf^39^.

This study implements a trait-based Climate Vulnerability Assessment (CVA) that makes use of existing information to estimate the relative vulnerability of fishery resources in the NHCS, one of the most important fishing areas in the world. Understanding which NHCS key fishery resources require a more detailed examination of likely climate change impacts is crucial to provide direction for prioritisation of more appropriate research and monitoring programs, including the implementation of correlative or mechanistic approaches, and to allow the development and implementation of adaptive fisheries management plans to buffer the negative impacts of climate change and to maximise opportunities.

## Methods

**Figure 2:**
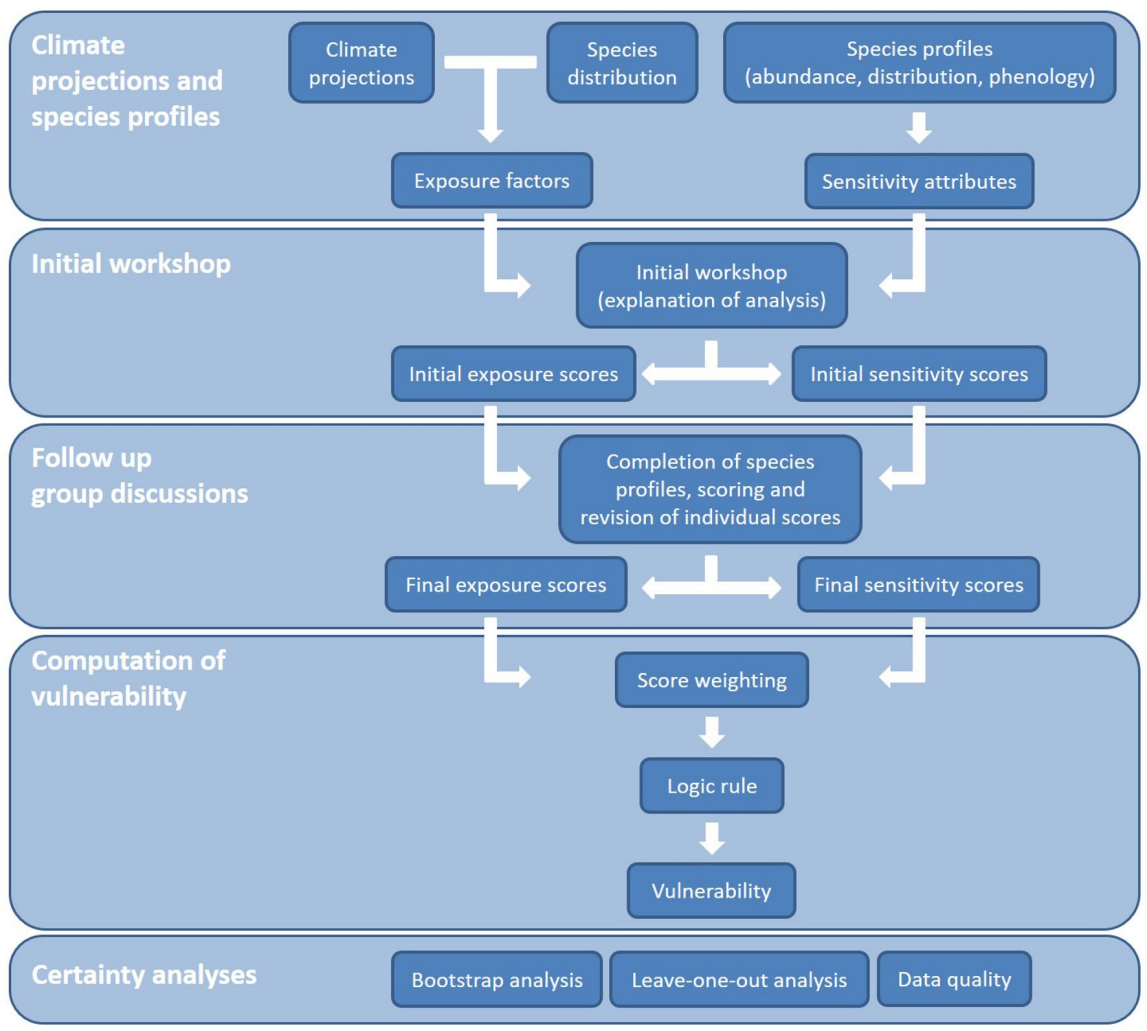
Flowchart of the Climate Vulnerability Assessment (Modified from a previous study^39^).

The CVA implemented in this study is based on the vulnerability framework derived by the Intergovernmental Panel on Climate Change (IPCC), where vulnerability is a function of exposure, sensitivity and adaptive capacity^40^. In this study, the vulnerability framework is implemented at the species level. Exposure is defined as the ‘climatic stimuli that have an impact on a species´, sensitivity is the ‘degree to which a species is affected by or is responsive to climate stimuli’, and vulnerability is the ‘degree to which a species is susceptible to injury, damage, or harm’. Exposure and sensitivity determine the potential impact which is regulated by the adaptive capacity, i.e., the biological responses that could reduce or mitigate the exposure or sensitivity^41^. However, most species characteristics that confer high adaptive capacity can also be traits that imply low sensitivity, causing methodological difficulties when both are included in species vulnerability assessments^42^. Therefore, species vulnerability assessments are often based on exposure and/or sensitivity^35, 38, 43, 44^, excluding adaptive capacity or addressing it within sensitivity. Here, the CVA for key fishery resources to the impacts of climate change in the NHCS was estimated as a function of exposure and sensitivity, with sensitivity including adaptive capacity traits. The CVA implemented in this study is explained in detail in the following lines, and it is summarised in Fig. 2.

### Participant experts

Twelve regional experts recruited from the Peruvian Marine Research Institute (IMARPE) and Peruvian universities participated in the assessment, including fisheries scientists, marine ecologists, and oceanographers with expertise on the species assessed and the area of study. IMARPE is the institution that monitors and assesses the Peruvian fishery resources for the government of Peru. Four experts assessed each species from one of three groups of species (benthic, demersal or pelagic). Previous assessments have involved two to five experts per species^37, 39, 45^; four experts were considered appropriate to detect consistencies and uncertainties for each species assessed.

### Species assessed

A total of 28 fishery resources were selected based on their ecological and commercial (higher catch volume and revenue) importance in the NHCS. The species were classified into three groups, i.e., benthic (n = 5), demersal (n = 12), and pelagic (n = 11; Table 1). The team of expert scientists made ‘species profiles’ based on information published in peer-reviewed scientific papers and in institutional scientific reports. The ‘species profiles’ included information on life history, population dynamics, geographic distribution, habitat, prey, abundance, and response to environmental variability and physical drivers that may be associated with climate change (Supplementary Methods S1: Species profiles). This information was used for the exposure and sensitivity assessment of each species.

**Table 1:** Key fishery resources in the Northern Humboldt Current System examined in the vulnerability assessment to climate change. *Commercial catch data only available from 2010–2015.

Group	Common name	Scientific name	Mean annual catch (t; 2010–2019)
Benthic	Changos octopus	*Octopus mimus*	2997
	Chocolate rock shell	*Thaisella chocolata*	2477
	Peruvian calico scallop	*Argopecten purpuratus*	49,682
	Purplish crab	*Platyxanthus orbignyi*	2009
	Ribbed mussel	*Aulacomya atra*	5334
Demersal	Corvina drum	*Cilus gilberti*	890
	Fine flounder	*Paralichthys adspersus*	317
	Flathead grey mullet	*Mugil cephalus*	18,485
	Humpback smooth-hound	*Mustelus whitneyi*	5623
	Lorna drum	*Sciaena deliciosa*	8250
	Lumptail searobin	*Prionotus stephanophrys*	2264
	Patagonian squid	*Doryteuthis gahi*	9370
	Peruvian banded croaker	*Paralonchurus peruanus*	1751
	Peruvian hake	*Merluccius gayi peruanus*	56,969
	Peruvian rock seabass	*Paralabrax humeralis*	2419
	Peruvian sea catfish	*Galeichthys peruvianus*	280*
	Peruvian weakfish	*Cynoscion analis*	4963
Pelagic	Blue shark	*Prionace glauca*	4138
	Chilean jack mackerel	*Trachurus murphyi*	87,003
	Common dolphinfish	*Coryphaena hippurus*	46,614
	Eastern Pacific bonito	*Sarda chiliensis chiliensis*	61,622
	Jumbo flying squid	*Dosidicus gigas*	430,147
	Mote sculpin	*Normanichthys crockeri*	2099*
	Pacific chub mackerel	*Scomber japonicus*	68,208
	Pacific sardine	*Sardinops sagax*	218
	Peruvian anchovy	*Engraulis ringens*	4,115,575
	Peruvian silverside	*Odontesthes regia*	7060
	Yellowfin tuna	*Thunnus albacares*	15,377

### Exposure

Exposure was assessed by examining changes in the mean of 10 exposure factors^37^: sea surface temperature, sea bottom temperature, air surface temperature, sea surface salinity, sea bottom salinity, sea surface pH, sea surface chlorophyll, primary productivity, precipitation, and sea level rise. Sea surface exposure factors represent the upper 10 m of the water column. Most factors were examined separately for the oceanic (> 100 km from the coast) and neritic (< 100 km from the coast) zones. Changes in inland precipitation were used as a proxy of the amount of water in streams and rivers that discharges in the coastal environment and may affect inshore species or particular life-history stages. Inland air surface temperature was used as a proxy for water temperatures in estuaries and nearshore areas^46, 47^.

The amount of change for most exposure factors was assessed from a set of 13–37 global climate models under the RCP 8.5 used in the IPCC Assessment Report 5 (IPCC AR5). RCP 8.5 represents the ‘business-as-usual’ scenario, which assumes little or no stabilization of greenhouse gas emissions by the year 2100. RCP 8.5 was used because global warming is likely to reach 1.5 °C above pre-industrial levels within the next three decades regardless of immediate reductions of greenhouse gas emissions^48^. The amount of change of each exposure factor was a measure of the change in mean climate conditions of the modeled future (2006–2055) relative to the past (1956–2005), resulting in units of standard deviations from the historical mean; these values were generated by the NOAA Ocean Climate Change web Portal (https://psl.noaa.gov/ipcc/ocn/).

The probabilities of the theoretical distribution of the future mean climate to change ≤ 1 standard deviation, > 1 standard deviation but ≤ 2 standard deviations, or > 2 standard deviations from the historical mean were used to classify the magnitude of change of each exposure factor in *low*, *medium*, or *high* categories, respectively (Supplementary Methods S2: Climate exposure factors). The experts took into account the magnitude of change of the exposure factor and its effect on the species assessed to score each exposure factor. The magnitude of change in sea level along the coasts of Peru defined according to the literature, and the species' dependence on coastal marine ecosystems (i.e., wetlands and estuaries) at any life-history stage were considered to score exposure to sea level rise (Supplementary Methods S3: Climate exposure factor: Sea level rise). Data of other environmental variables such as dissolved oxygen were not available at the NOAA Ocean Climate Change web Portal and therefore were not included in the assessment.

### Sensitivity

Species sensitivity was estimated by examining 13 biological attributes related to abundance, distribution or phenology (Table 2). Current status taken from the IUCN Red List (https://www.iucnredlist.org/) was included as an attribute of abundance in the absence of regional biomass estimates. Each attribute was classified as one of three categories of sensitivity: (1) *low* sensitivity, the species has a high capacity to respond to the impacts of climate change and is therefore at lower risk; (2) *medium* sensitivity; and (3) *high* sensitivity, the species has a low capacity to respond to the impacts of climate change and is at higher risk^35^.

**Table 2:** Sensitivity attributes, categories and criteria used to assess the relative sensitivity of key fishery resources to climate change in the Northern Humboldt Current System, adapted from a previous study^35^.

Sensitivity attribute		Category		
		(1) *Low* sensitivity, high capacity to respond (lower risk)	(2) *Medium* sensitivity	(3) *High* sensitivity, low capacity to respond (higher risk)
Abundance	*Fecundity*–egg production (total fecundity)	> 20,000 eggs per year	100–20,000 eggs per year	< 100 eggs per year
	*Recruitment period*—successful recruitment event that sustains the abundance of the fishery	Consistent recruitment events every 1–2 years	Occasional and variable recruitment period	Highly episodic recruitment event
	Average age at maturity	≤ 2 years	2–10 years	> 10 years
	*Generalist vs. specialist*—food and habitat	Reliance on neither habitat or prey	Reliance on either habitat or prey	Reliance on both habitat and prey
	*Current status*—Biomass	Robust	Vulnerable	Uncertain/Threatened
Distribution	*Capacity for larval dispersal or larval duration*—hatching to settlement (benthic species), hatching to yolk sac re-adsorption (pelagic species)	> 2 months	2–8 weeks	< 2 weeks or no larval stage
	*Capacity for adult/juvenile movement*—lifetime range post-larval stage	> 1000 km	10–1000 km	< 10 km
	*Physiological tolerance*—latitudinal coverage of adult species as a proxy of environmental tolerance	> 20° latitude	10–20° latitude	< 10° latitude
	*Spatial availability of unoccupied habitat for most critical life stage*—ability to shift distributional range	Substantial unoccupied habitat; > 6° latitude or longitude	Limited unoccupied habitat; 2–6° latitude or longitude	No unoccupied habitat; 0–2° latitude or longitude
Phenology	*Environmental variable as a phenological cue for spawning or breeding*—cues include salinity, temperature, currents, and freshwater flows	No apparent correlation of spawning to environmental variable	Weak correlation of spawning to environmental variable	Strong correlation of spawning to environmental variable
	*Environmental variable as a phenological cue for settlement or metamorphosis*	No apparent correlation to environmental variable	Weak correlation to environmental variable	Strong correlation to environmental variable
	*Temporal mismatches of life-cycle events*—duration of spawning, breeding or moulting season	Continuous duration; > 4 months	Wide duration; 2–4 months	Brief duration; < 2 months
	*Migration* (seasonal and spawning)	No migration	Migration is common for some of the population	Migration is common for the whole population

### Scoring system

An initial workshop was carried out to define the groups of experts and the sensitivity attributes, and to explain the CVA. A ‘tallies’ system was adapted from a previous study^49^ to identify the expert’s uncertainty for a given scoring. Four experts independently assigned four tallies each, among the three categories (*low*, *medium*, *high*) of every exposure factor and sensitivity attribute for each species of their corresponding species group (benthic, demersal or pelagic). Four tallies were assigned to one category if there was certainty in the information. As a precautionary approach, four tallies were assigned to the *high* category if there was no information about the effect of the exposure factor on the species, or if there was no information on the sensitivity attribute. The four tallies were assigned to the *low* category for exposure factors that do not affect a species. The four tallies were spread across the three categories if there was uncertainty, allowing the expert to choose one category as the most likely. During the initial workshop, the experts had the chance to score species whose ‘species profiles’ were already complete. Over the following six months, the experts completed the pending ‘species profiles’ and scored each species within their respective group. Each group of experts discussed the scores and if deemed necessary made changes (although they were not required to reach consensus), and then recorded the final scores.

### Score weighting

A weighted score was calculated for each exposure factor and sensitivity attribute^49^:

$$\left( {\left( {{\text{L}} \times 1} \right) + \left( {{\text{M}} \times 2} \right) + \left( {{\text{H}} \times 3} \right)} \right){ / }\left( {{\text{L}} + {\text{M}} + {\text{H}}} \right)$$

where L is the total number of tallies in the (1) *low* category, M is the total number of tallies in the (2) *medium* category, and H is the total number of tallies in the (3) *high* category. Cumulative weighted scores of exposure and sensitivity were estimated separately for each species, and these were used to do the species exposure and sensitivity rankings, respectively. Cumulative weighted scores of exposure factors were summed across species to detect the factors with greater effect on the species; cumulative weighted scores of exposure and sensitivity were also calculated at the species group level. Means of weighted scores were estimated for sensitivity attributes at the abundance, distribution, and phenology levels.

### Logic rule

A logic rule was adapted from a previous study^49^ to calculate a 1 to 3 component score for each species. A species received a *‘high’* component score (value of 3) if 3 or more exposure factor or sensitivity attribute weighted scores were ≥ 2.5. A species received a *‘medium’* component score (value of 2) if 3 or more exposure factor or sensitivity attribute weighted scores were 1.5–2.49. A species received a *‘low’* component score (value of 1) if 3 or more exposure factor or sensitivity attribute weighted scores were < 1.5.

### Vulnerability

The vulnerability rank was estimated by multiplying the numerical values of the exposure and sensitivity component scores, e.g., if exposure component = 1 and sensitivity component = 3, Vulnerability = 1 × 3 = 3. The vulnerability rank was classified as 1–2) “*low”*, 3–4) “*medium”*, 6) “*high”*, and 9) “*very high”*. The category *“very high”* was used to identify the species that were estimated to be most at risk.

### Certainty analyses

Bootstrap analysis was used to calculate the certainty of exposure, sensitivity, and vulnerability categories. Certainty refers to the percentage of bootstrapped iterations that were identical to the original distribution of: (1) exposure and sensitivity bins, and (2) vulnerability categories. To calculate certainty, scores across all experts for each exposure factor (n = 16; 4 experts, 4 tallies) and sensitivity attribute (n = 16; 4 experts, 4 tallies) were drawn 10,000 times randomly with replacement. The logic rule was applied after each iteration, and the relative frequencies of the exposure factors (n = 10) and sensitivity attributes (n = 13) that were assigned to each bin (*low, medium, high*) were recorded. The overall vulnerability category was also calculated for each iteration, and the relative frequencies of the iterations that scored in each vulnerability category were recorded. Certainties were classified as very high (> 95%), high (91–95%), moderate (70–90%), and low (< 70%).

A leave-one-out analysis was also used to assess the influence of each exposure factor and sensitivity attribute on the vulnerability of the species by removing each factor and attribute and re-applying the logic rule; certainty was recorded as the percentage of vulnerability scores that were identical to the original vulnerability estimates.

The quality of the information in the ‘species profiles’ used to score each exposure factor and sensitivity attribute was classified in four categories: (0) no data, (1) reviewer judgement, (2) related data, and (3) high quality data (Table 3). Relative frequencies of data quality categories were examined at the species group level and at the type of sensitivity attributes level. Frequencies of data quality categories were also shown in matrices of species *vs* exposure factors, and species *vs* sensitivity attributes. The data quality categorisation was useful to provide a guideline of the information available and on key information gaps. Critical gaps in information may allow identification of areas for future research to further inform future CVAs^37, 49^.

**Table 3:** Data quality scores for the vulnerability assessment of key fishery resources to climate change in the Northern Humboldt Current System (adapted from a previous study^49^).

Data quality score	Description
3	**High quality data:** The score is based on data which have been observed, modeled or empirically measured for the species and area of interest, and that comes from a reputable source
2	**Related data:** The score is based on data which has a higher degree of uncertainty. The data used to score the attribute may be based on related or similar species, come from outside the study area, or the reliability of the source may be limited
1	**Reviewer judgement:** The attribute score reflects the judgement of the reviewer and is based on their general knowledge of the species, or other related species, and their relative role in the ecosystem
0	**No data:** No information to base an attribute score on. Very little is known about the species or related species and there is no basis for forming an expert opinion

## Results

### Exposure

**Figure 3:**
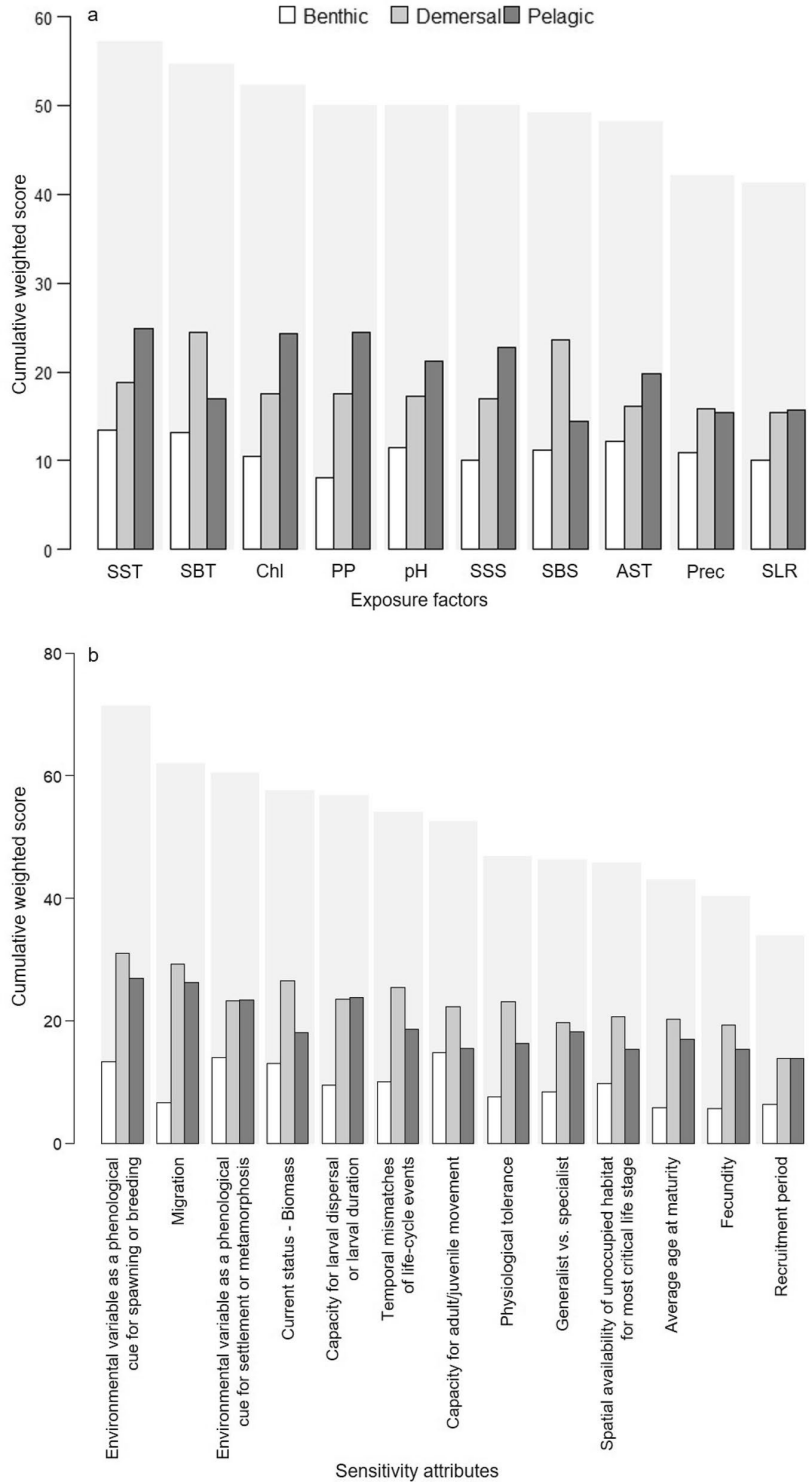
Cumulative weighted score of (**a**) climate exposure factors and (**b**) sensitivity attributes. Thin bars correspond to the cumulative weighted score of species groups. Thick light shaded bars correspond to the total cumulative weighted score. SST: Sea surface temperature; SBT: Sea bottom temperature; Chl: Chlorophyll concentration; PP: Primary productivity; pH: as a proxy for ocean acidification; SSS: Sea surface salinity; SBS: Sea bottom salinity; AST: Air surface temperature; Prec: Precipitation; SLR: Sea level rise.

Sea surface temperature and sea bottom temperature were the exposure factors with highest cumulative weighted scores summed across species, i.e., 57 and 55, respectively (Fig. 3a). Based on the literature, temperature was considered by the experts to be important for changes in distribution, abundance, reproductive seasons, larval development, growth rates, size structure, and recruitment. Chlorophyll concentration and primary productivity had high cumulative weighted scores and were considered to be associated with the availability of nutrients and food; pH and sea surface salinity also had relatively high cumulative weighted scores. Air surface temperature, precipitation and sea-level rise had the lowest cumulative weighted scores but were considered crucial for the few species or life-history stages that occur in estuaries and near shore.

**Figure 4:**
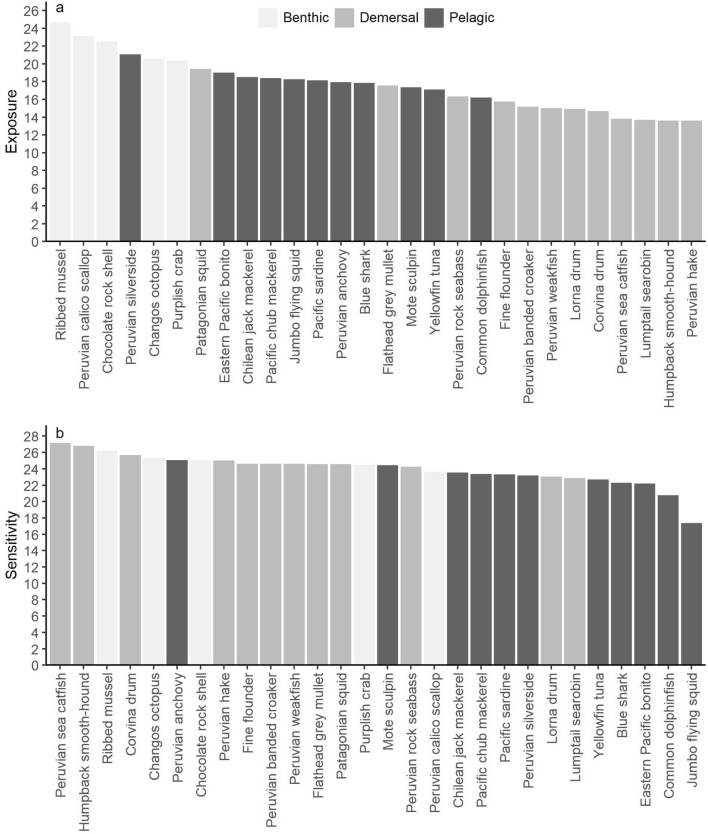
Rankings of (**a**) exposure and (**b**) sensitivity (cumulative weighted scores) of key fishery resources to climate change in the Northern Humboldt Current System. The rankings can reflect high scores due to known exposure/sensitivity or due to gaps in information.

**Figure 5:**
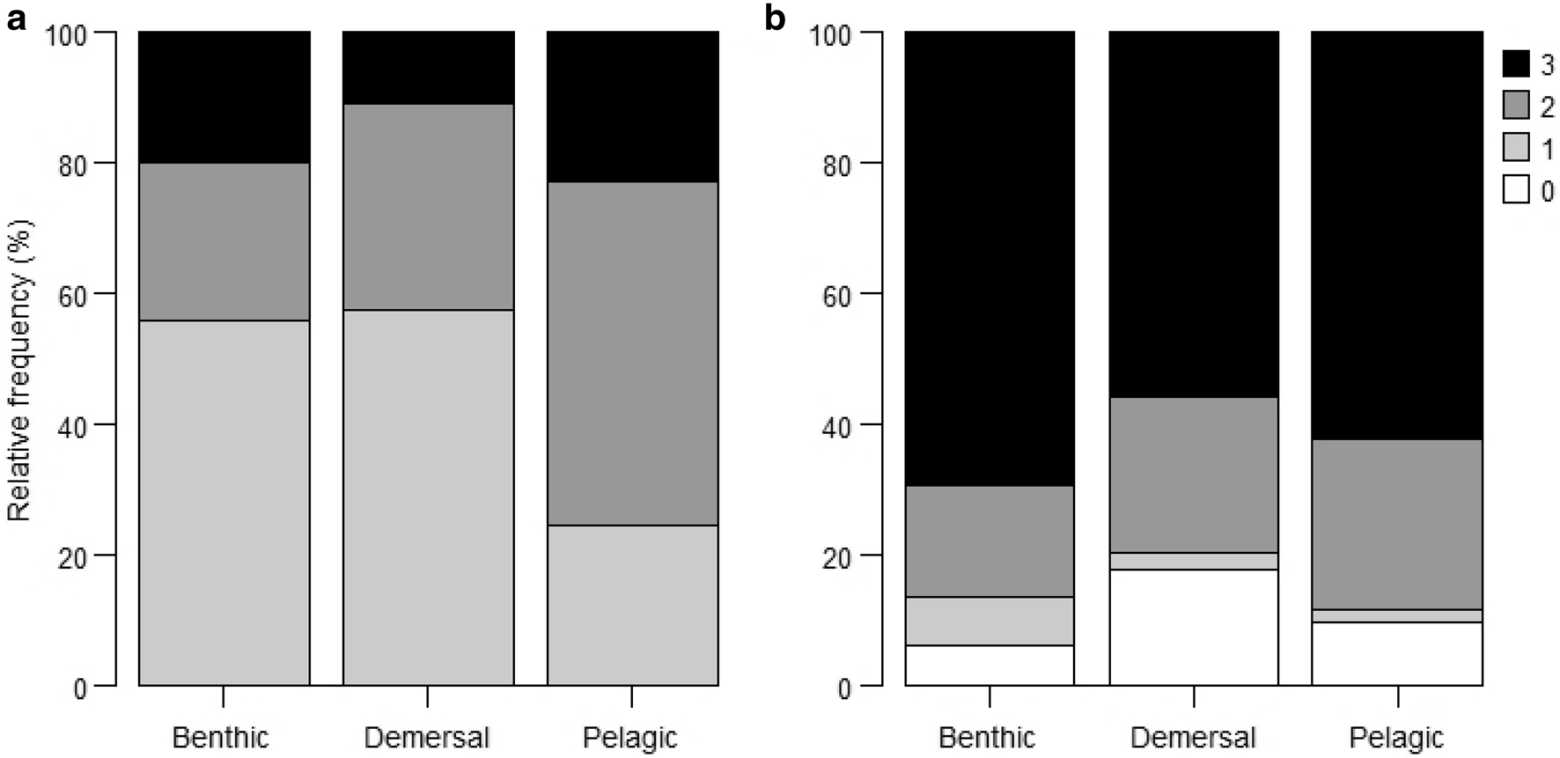
Data quality of (**a**) climate exposure factors and (**b**) sensitivity attributes per group of key fishery resources in the Northern Humboldt Current System. Data quality score: 3) High quality data; 2) Related data; 1) Reviewer judgement; 0) No data.

Benthic species had the highest exposure (22.66 ± 0.79 SE), followed by pelagic (18.13 ± 0.37 SE) and demersal (15.30 ± 0.51 SE) species. The overall cumulative exposure scores at the species level ranged from 13.6 to 24.6, with benthic species such as ribbed mussel, Peruvian calico scallop, and chocolate rock shell being at the top of the exposure ranking (Fig. 4a). Pelagic species had the greatest proportion of high quality data (data quality = 3; 23%) and related data (data quality = 2; 53%); benthic species had 20% high quality data and 24% related data, whereas demersal species had 11% high quality data and 32% related data. Reviewer judgement (data quality = 1) was common for benthic (56%) and demersal (57%) species (Fig. 5a; Supplementary Fig. S1). Bootstrap analysis showed that 61% of the species assessed had very high certainty (> 95%) in exposure category, 7% had high certainty (91–95%), 18% had moderate certainty (70–90%), and 14% had low certainty (< 70%; Supplementary Table S1).

### Sensitivity

Environmental variables as a phenological cue for spawning or breeding, and for settlement or metamorphosis, were amongst the attributes with the greatest contribution to the sensitivity of species due to the lack of information (Fig. 3b). Benthic species had the highest sensitivity (24.94 ± 0.43 SE), followed by demersal (24.82 ± 0.37 SE) and pelagic (22.47 ± 0.71 SE) species. All groups were more sensitive to phenology attributes (2.21 ± 0.05 SE) compared with distribution (1.80 ± 0.05 SE) and abundance (1.58 ± 0.04 SE). Sensitivity to phenology was relatively greater for demersal species (2.27 ± 0.05 SE) than for benthic (2.20 ± 0.15 SE) and pelagic (2.16 ± 0.08 SE) species. The cumulative sensitivity scores at the species level ranged from 17.4 to 27.2. Nearly half the species had relatively high sensitivity, including four benthic species (e.g., ribbed mussel, Changos octopus, chocolate rock shell), ten demersal species (e.g., Peruvian hake, Patagonian squid), and one pelagic species (i.e., Peruvian anchovy) (Fig. 4b).

High quality data comprised most of the information at the sensitivity attribute level, i.e., abundance (69%), distribution (63%), and phenology (49%); related data comprised 27% of abundance, 24% of distribution, and 28% of phenology information. Greater data gaps (data quality = 0) were detected in phenology attributes (29%), whereas distribution and abundance attributes had data gaps of only 8% and 4% respectively (Supplementary Fig. S2). High quality data were predominant for benthic (69%), pelagic (62%), and demersal (56%) species, whereas related data contributed 17% for benthic, 23% for demersal, and 26% for pelagic species. Greater data gaps were detected for demersal (18%) and pelagic (10%) species (Fig. 5b; Supplementary Fig. S3). Approximately 47% of the species assessed had very high certainty (> 95%) in the sensitivity category, 18% had high certainty (91–95%), 21% had moderate certainty (70–90%), and 14% had low certainty (< 70%; Supplementary Table S1).

### Vulnerability

Overall, benthic species were more vulnerable (7.40 ± 1.03 SE) than pelagic (4.55 ± 0.28 SE) and demersal (3.92 ± 0.42 SE) species. Three benthic species were ranked as “*very high”* vulnerability, which comprised 11% of the species assessed; these were the ribbed mussel, chocolate rock shell, and Peruvian calico scallop. Nearly 25% of the species assessed were ranked as “*high*” vulnerability; these were one benthic (i.e., Changos octopus), four demersal (e.g., flathead grey mullet, Patagonian squid), and two pelagic species (i.e., Pacific chub mackerel, yellowfin tuna). Most species (57%) were ranked as “*medium”* vulnerability; these were one benthic, seven demersal (e.g., fine flounder, lorna drum), and eight pelagic species (e.g., Peruvian anchovy, Chilean jack mackerel, common dolphinfish, jumbo flying squid).

**Figure 6:**
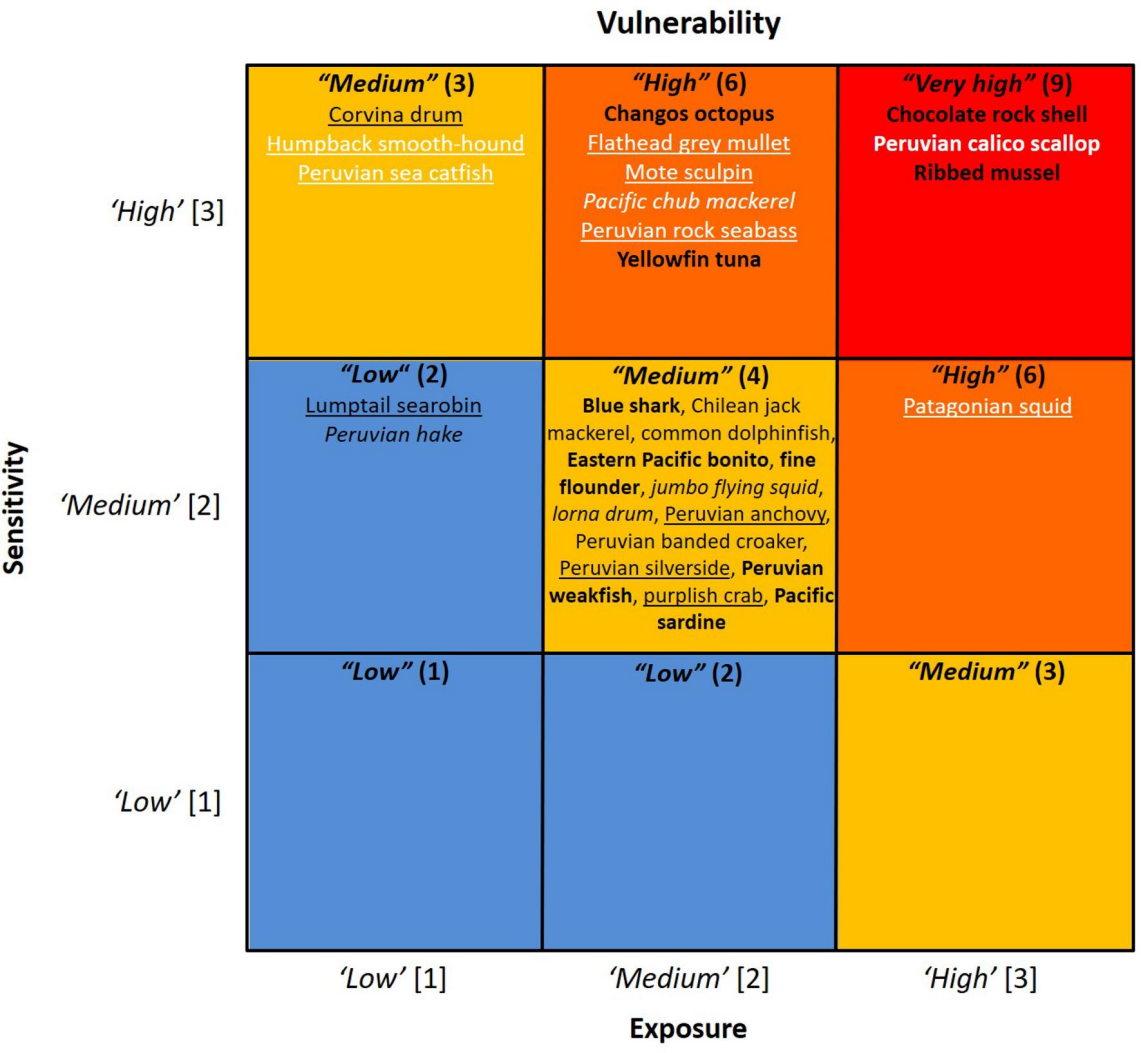
Vulnerability matrix, estimated from exposure to climate factors and sensitivity attributes, of key fishery resources to climate change in the Northern Humboldt Current System. Certainty from the bootstrap analysis is indicated by the style of font: > 95%) bold; 91–95%) regular; 70–90%, *italic*; < 70%, underline. The vulnerability ranking of species indicated in white font changed with the leave-one-out analysis.

Only two demersal species had “*low”* vulnerability (i.e., Peruvian hake, lumptail searobin), comprising 7% of the species assessed. Approximately 36% of the species assessed had very high certainty (> 95%) in the vulnerability category, 11% had high certainty (91–95%), 18% had moderate certainty (70–90%), and 35% had low certainty (< 70%; Fig. 6; Supplementary Table S1). The leave-one-out analysis did not change the vulnerability category of 20 out of the 28 species; the other eight species with lower expert consensus decreased one vulnerability category. The Peruvian calico scallop changed from “*very high”* to “*high”* vulnerability. Flathead grey mullet, mote sculpin, Pacific chub mackerel, Peruvian rock seabass, and Patagonian squid changed from “*high”* to “*medium”* vulnerability. Peruvian sea catfish and humpback smooth-hound changed from “*medium”* to “*low”* vulnerability (Fig. 6; Supplementary Table S2).

## Discussion

Peruvian waters in the NHCS sustain the second most important fisheries production in the world^5^. Historically, the resources underpinning these fisheries are subject to substantial climate variability. However, climate change over the next few decades will likely see this variation exacerbated and the associated socio-economic effects amplified. Here, through a transparent and repeatable CVA we were able to rank 28 key fishery resources according to their estimated relative level of vulnerability to the impacts of climate change. Some of these species have the world’s largest landings, i.e., Peruvian anchovy, Pacific chub mackerel, and Chilean jack mackerel^5^, and contribute the majority of the total annual fisheries production in Peru^6^. Our study shows that the impacts of climate change on Peruvian fishery resources are expected to be substantial, particularly for benthic species.

### Vulnerability ranking

Benthic species were ranked the most vulnerable, with higher exposure and sensitivity compared with demersal and pelagic species. In general, species and ecosystems that are highly associated with the ocean floor are at greater risk than species that inhabit the water column^50^, with shallow water invertebrate species likely being more responsive to climate change^51^. Benthic species in the adult stage are characterized by limited movement capacity, and must respond in situ to multiple exposure factors. Accordingly, the only species ranked with “*very high*” vulnerability were benthic molluscs, i.e., ribbed mussel, chocolate rock shell and Peruvian calico scallop. Significant acidification in NHCS coastal waters has been predicted under climate change scenarios^52^, which may affect the physiology, growth and mineralization of benthic calcifiers^53^. However, the response of these molluscs to acidification may be variable depending on the life cycle stage^54^. Intensification of heavy rains induced by climate change increases river discharge, decreasing salinity and increasing turbidity, causing higher mortality of benthic species such as the Peruvian calico scallop^55^. This species may decrease in abundance at Sechura bay in the north coast of Peru, where heavy rains cause large river discharges.

The pelagic group was the second most vulnerable, with 27% of the pelagic species ranked with “*high*” vulnerability. The Peruvian anchovy is the most important commercial species in terms of landings in Peru. Two stocks are managed within the Peruvian EEZ; the northern-central stock is located between 2°S and 16°S and contributes > 90% of Peruvian anchovy landings, and the southern stock that is located to the south of 16°S^6^. Consistent with our estimate of “*medium*” vulnerability for the Peruvian anchovy by 2055, the rapid recovery of this species from previous El Niño conditions^56^ suggest high resilience to physical and chemical variability. Nevertheless, climate change is expected to have negative impacts on the Peruvian anchovy population in the longer term; its biomass is predicted to decrease after mid-century due to oceanic warming, and due to a reduction of coastal upwelling and primary productivity^57^. Accordingly, total biomass has been estimated to decrease 8.2–13.9% per decade for the period 2009–2100^58^. Coastal warming events, together with salinity and oxygen fluctuations, have already shown effects on the spatial distribution^59^, population structure, reproductive season, and fecundity of this species^28^. In addition, projected southward change in distribution closer to the coast^57, 58^ can affect the distribution of the fishing fleet, with the northern fleet consuming more fuel to reach southern fishing grounds and to return to port. Peruvian anchovy juveniles tend to have a more coastal distribution compared with adults, which facilitates targeting the adult portion of the population. However, the overlap in distribution of juveniles and adults associated with warming events results in fishing pressure on juveniles, with negative impacts on the Peruvian anchovy stocks^24^.

The demersal group had the lowest vulnerability across the species groups, with only 25% of the species ranked with *“high”* vulnerability. Demersal species are anticipated to change in distribution, most likely poleward and towards deeper waters, which is a common response to warming^16, 19, 60^. Peruvian hake is the most abundant demersal commercial species in the NHCS^61^. Climate-driven distributional changes of Peruvian hake are expected; this species has been responsive to El Niño 1991–1993 when large individuals moved from the traditional fishing areas in Peruvian waters^62^. Increase in fuel consumption and augmented operational costs for the Peruvian hake industrial fleet are anticipated under such changes^63^. Changes in upwelling conditions associated with climate change may impact the movement of eggs (mid-waters) and larvae (onshore)^64^, although the directionality of such changes requires further examination^13^.

### Caveats of the CVA

Availability, selection and inclusion of environmental data in the exposure analysis are partly determined by our capacity to monitor the environmental variables. Temperature is relatively easy to record, which facilitates documenting and better understanding the impacts of oceanic warming on marine biota compared with the impacts caused by other exposure factors. For example, data of dissolved oxygen were not available and therefore were not included in the assessment despite subsurface deoxygenation being characteristic of the Humboldt Current System^65, 66^ and the oxycline shoaling in Peruvian coastal waters since the 1990s^67^. Consequently, the oxygen regime is of particular interest in the Humboldt Current System and must not be overlooked, particularly given the global trend of ocean deoxygenation^68^. Low oxygen concentration affects active species via increased metabolic demand^69^ as well as species with limited movement capacity, such as scallops^70^. Fluctuations of the oxycline depth vary the habitat compression of the epipelagic realm, altering the distribution and behavior of nekton^71^ and their catchability by fishing fleets. The shift from Peruvian anchovy to Pacific sardine appeared to be influenced by the change from relatively low oxygen concentration to increased oxygen concentration^67^. Oxygen changes also impact the distribution, abundance and diversity of benthic^72^ and demersal species. Benthic colonization of sublittoral bottoms off central Peru occur under oxygenation events associated with El Niño^72, 73^, whereas the latitudinal distribution of Peruvian hake appears to be modulated by the intensity of the southward subsurface flows that ventilate the water column^62^. The reduction of upwelling associated with thermal stratification^12^ may trigger changes in the distribution and abundance of several species, with expected impacts on commercial catches^74^. Changes in salinity linked to freshwater flow^75^, and sea-level rise will also affect species that migrate towards the coast, or near rivers or estuaries to spawn or to make use of those habitats as nursery grounds^76^. The present study highlights the need for mechanistic studies that will provide more information on the effect of climate exposure factors on the species assessed, which would benefit future CVAs.

Approximately 56% of the information regarding the effect of exposure factors on the fishery resources was data characterized as high quality data or from related data, showing some level of uncertainty in the exposure component of the assessment. In addition, the global climate models used in this CVA as a proxy for the amount of change of most exposure factors are generally consistent with downscaled physical trends; however, high uncertainty in downscaled biogeochemical trends highlights the need for more realistic climate projections of the NHCS^13^. CVAs are usually limited in this sense, as they often lack regionalised projections under climate change scenarios^77^. The lack of available phenology information was common in this study, as is the case in other species sensitivity assessments^35, 38^. Given the precautionary approach used for the sensitivity component of the assessment (i.e., lack of information was given the highest score for any given sensitivity attribute), sensitivity and as a consequence vulnerability may have been overestimated for some species. Overestimation is preferable than underestimation when uncertainties derived from data gaps are common as it is better to protect species with vulnerability lower than that estimated compared to not protecting a species with vulnerability higher than what was estimated. All CVAs (including trait-based, correlative, and mechanistic approaches) have a degree of uncertainty^34^, which should be estimated to demonstrate how robust the data are and also to provide caveats to researchers, resource managers and policy makers that may make decisions based on the assessment. The bootstrap analysis calculated high certainties (> 90%) for 68% of the species in terms of exposure and for 65% of the species in terms of sensitivity, and only for 47% of the species in terms of vulnerability. A small proportion of species had low certainties (< 70%) for exposure (14%) and sensitivity (14%), whereas a greater proportion of species had low certainty for vulnerability (35%). The leave-one-out analysis resulted in the decrease of one vulnerability category for eight species, including the highly valued Peruvian calico scallop and the Pacific chub mackerel. Nevertheless, this assessment aims not only to provide pertinent information to prioritise the most vulnerable species but also to identify important information gaps and to direct research efforts into better understanding those gaps in knowledge. Research efforts should be directed to increase our knowledge on the effects of pH and dissolved oxygen on most of the fishery resources assessed, and on the effects of temperature, salinity, productivity, and precipitation on benthic and demersal species. One of the benefits of the CVA approach is that it can be repeated and updated regularly^35^, extended by including additional environmental variables, and complemented with correlative and mechanistic approaches^33, 34^.

### Adaptation plans

Adaptation plans will depend on the biological characteristics of the fishery resources, on the characteristics of the fishery, and on the capacity of fishers to adapt to changes associated with climate change. Changes in the distribution and abundance of fishery resources can affect accessibility for artisanal and industrial fishing fleets, which may require changes in size and capacity of fishing vessels, characteristics of fishing gears, and in fuel consumption^58, 63, 78^. Conflicts can arise between neighbouring human communities and nations as resources decrease in abundance from common fishing areas but increase in abundance in other areas. For instance, Peruvian waters were projected to receive no new climate-driven fishery stocks by 2100^79^, although some current Peruvian fishery stocks are likely to move within the Peruvian EEZ or somewhere else driven by climate change. Participation of fishers, scientists, resource managers, and policy makers is vital in the climate change adaptation process, as it is planning ahead for cooperative management. Data sharing between institutions and between nations will allow a better understanding and management of shared stocks. The models used to anticipate changes in distribution and abundance must be robust and reliable, and the level of uncertainty must be acknowledged to make the best adaptive management decisions^79^.

Fishers that are most vulnerable to climate change are those who do not use technological innovations (e.g., GPS, radio-communications, etc.), who do not have other job alternatives, or with limited school education level^80^. Capacitation to fishers on the use of better technology and financial support for the adaptation of fishing gears will increase their capacity to access fishery resources that are responsive to climate change. Added value of fishery products and market diversification will buffer financial loss, to some extent, due to projected declines in catch potential in the NHCS^25, 30, 31^. In addition, income diversification will reduce dependency on fisheries and fish products, e.g., ecotourism, aquaculture, sub-aquatic activities, etc. A number of additional strategies have already been proposed for the adaptation of artisanal fisheries to climate change in Peru. Some of these are to create awareness amongst stakeholders on climate change impacts on the artisanal fishery, prioritize research plans for the development of the artisanal fishery, improvement of the artisanal fleet and fishing gear, promote the sustainable extraction of fishery resources, and increased control and sanction of illegal fishing and poor fishing practices^81^. These are some of the strategies that can be implemented to decrease the vulnerability of people whose livelihoods depend on fishery resources^82–84^.

Adaptive management strategies already being implemented in the NHCS can be transferred to the climate change context. Substantial efforts are currently taking place to monitor physical, chemical, and biological conditions of the marine ecosystem to assess effects on the Peruvian anchovy population dynamics^24^. Further fishing pressure on juvenile anchovy due to changes in distribution of the stock associated with climate change^57, 58^ can be regulated with the juvenile total allowable catch (TAC) that is currently implemented during the stock assessment process^24^. The objective of this strategy is to protect the stock by closing the fishery if juvenile TAC has been reached regardless of total TAC^24^. Adaptation management strategies can be developed and implemented for Peruvian fishery resources at the biological group (benthic, demersal, pelagic), stock, or life cycle stage levels to ensure the sustainability of the fisheries in the face of climate change.

## Conclusions

Coastal benthic species had the highest vulnerability as a result of the highest exposure and sensitivity from across the species groups, with chocolate rock shell, Peruvian calico scallop, and ribbed mussel ranked with “*very high*” vulnerability. Our findings suggest the need for immediate monitoring of benthic species in the face of climate change, and of other species with “*high*” vulnerability that contribute significantly to the total catch production in the NHCS, i.e., Pacific chub mackerel. Nearly 57% of the species assessed were ranked with “*medium*” vulnerability, including the Peruvian anchovy, jumbo flying squid, Chilean jack mackerel, and common dolphinfish, with anticipated variable responses across species.

The balance between negative and positive impacts of climate change on the fishery socio-ecological system will be determined in part by our capacity to anticipate biological responses to climate change, and our ability to minimize threats and optimise opportunities. In this sense, transdisciplinary research to increase our understanding on the effects of climate change at the species and ecosystem levels, and vulnerability assessments of the biological^37, 39^ and social components of the fisheries^84^ are necessary. This will enable advice to fishers, stakeholders, and governments to co-manage human interactions with fisheries and ecosystems^85^. Better support to human communities whose livelihoods depend on fishery resources under the effects of climate change would also be possible, e.g., by the adaptation of fishing gears, use of better technology, added value to fish products, income diversification, and financing mechanisms to alleviate socio-economic impacts derived from climate change^82–84^.

## Supplementary Information

Supplementary Information.

## Data Availability

Data are available upon request.

## References

[1] Penven P, Echevin V, Pasapera J, Colas F, Tam J (2005). Average circulation, seasonal cycle, and mesoscale dynamics of the Peru Current System: A modeling approach. J. Geophys. Res. Oceans.

[2] Montes I, Colas F, Capet X, Schneider W (2010). On the pathways of the equatorial subsurface currents in the Eastern Equatorial Pacific and their contributions to the Peru–Chile Undercurrent. J. Geophys. Res..

[3] Chaigneau A (2013). Near-coastal circulation in the Northern Humboldt Current System from shipboard ADCP data. J. Geophys. Res. Oceans.

[4] Bakun A, Weeks S (2008). The marine ecosystem off Peru: What are the secrets of its fishery productivity and what might its future hold?. Prog. Oceanogr..

[5] FAO. *The State of World Fisheries and Aquaculture 2020. Sustainability in Action* 206 (FAO, Rome, 2020). 10.4060/ca9229en.

[6] PRODUCE. *Anuario Estadístico Pesquero y Acuícola 2017* 200 (PRODUCE, Lima, 2018).

[7] Gutiérrez D, Akester M, Naranjo L (2016). Productivity and sustainable management of the Humboldt Current Large Marine Ecosystem under climate change. Environ. Dev..

[8] PRODUCE. *Anuario Estadístico Pesquero y Acuícola 2020* 182 (PRODUCE, Lima, 2021).

[9] Allison EH (2009). Vulnerability of national economies to the impacts of climate change on fisheries. Fish. Fish..

[10] Barange M (2014). Impacts of climate change on marine ecosystem production in societies dependent on fisheries. Nat. Clim. Change.

[11] FAO. Perfiles de Pesca y Acuicultura por Países. Perú. Hojas de datos de perfiles de los países. In *Departamento de Pesca y Acuicultura de la FAO* (FAO, Rome, 2018). http://www.fao.org/fishery/facp/PER/es Accessed October 2020.

[12] Oerder V (2015). Peru–Chile upwelling dynamics under climate change. J. Geophys. Res. Oceans.

[13] Echevin V (2020). Physical and biogeochemical impacts of RCP8.5 scenario in the Peru upwelling system. Biogeosciences.

[14] ECLAC. *Climate Variability, Dynamics and Trends. The Effects of Climate Change on the Coasts of Latin America and the Caribbean* (United Nations, Santiago, 2015).

[15] Poloczanska ES (2013). Global imprint of climate change on marine life. Nat. Clim. Change.

[16] Poloczanska ES (2016). Responses of marine organisms to climate change across oceans. Front. Mar. Sci..

[17] Stocker, T.F. *et al.* Technical summary. In *Climate Change 2013: The Physical Science Basis. Contribution of Working Group I to the Fifth Assessment Report of the Intergovernmental Panel on Climate Change* (eds. Stocker, T.F. *et al.*) 33–115 (Cambridge University Press, 2013).

[18] Bindoff, N.L. *et al.* Changing Ocean, marine ecosystems, and dependent communities. In *IPCC Special Report on the Ocean and Cryosphere in a Changing Climate* (eds. Pörtner, H.O. *et al*.) 447–587 (2019).

[19] Dulvy NK (2008). Climate change and deepening of the North Sea fish assemblage: A biotic indicator of warming seas. J. Appl. Ecol..

[20] Sunday JM, Bates AE, Dulvy NK (2012). Thermal tolerance and the global redistribution of animals. Nat. Clim. Change.

[21] Burrows MT (2014). Geographical limits to species-range shifts are suggested by climate velocity. Nature.

[22] Vinagre C (2019). Upper thermal limits and warming safety margins of coastal marine species—Indicator baseline for future reference. Ecol. Indic..

[23] Edwards M, Richardson AJ (2004). Impact of climate change on marine pelagic phenology and trophic mismatch. Nature.

[24] Bahri, T. *et al.* (eds.) *Adaptive Management of Fisheries in Response to Climate Change*. FAO Fisheries and Aquaculture Technical Paper No. **667** (Rome, FAO, 2021). 10.4060/cb3095en.

[25] Lam VWY (2020). Climate change, tropical fisheries and prospects for sustainable development. Nat. Rev. Earth. Environ..

[26] Barber RT, Chavez F (1983). Biological consequences of El Niño. Science.

[27] Chavez FP, Ryan J, Lluch-Cota SE, Ñiquen M (2003). From anchovies to sardines and back: Multidecadal change in the Pacific Ocean. Science.

[28] Ñiquen M, Bouchon M (2004). Impact of El Niño events on pelagic fisheries in Peruvian waters. Deep Sea Res. Part II Top. Stud. Oceanogr..

[29] Richard J, Morley SA, Thorne MAS, Peck LS (2012). Estimating long-term survival temperatures at the assemblage level in the marine environment: Towards macrophysiology. PLoS ONE.

[30] Golden CD (2016). Nutrition: Fall in fish catch threatens human health. Nature.

[31] Lam VWY, Cheung WWL, Reygondeau G, Sumaila R (2016). Projected change in global fisheries revenues under climate change. Sci. Rep..

[32] Poulain, F., Himes-Cornell, A. & Shelton, C. Chapter 25: Methods and tools for climate change adaptation in fisheries and aquaculture. In *Impacts of Climate Change on Fisheries and Aquaculture: Synthesis of Current Knowledge, Adaptation and Mitigation Options*. FAO Fisheries and Aquaculture Technical Paper No. 627 (eds. Barange, M. *et al.*) 628 (FAO, 2018).

[33] Pacifici M (2015). Assessing species vulnerability to climate change. Nat. Clim. Change.

[34] Foden WB (2018). Climate change vulnerability assessment of species. WIREs Clim. Change.

[35] Pecl G (2014). Rapid assessment of fisheries species sensitivity to climate change. Clim. Change.

[36] Hobday AJ (2011). Ecological risk assessment for the effects of fishing. Fish. Res..

[37] Hare JA (2016). A vulnerability assessment of fish and invertebrates to climate change on the Northeast U.S. Continental Shelf. PLoS ONE.

[38] Ortega-Cisneros K (2018). Assessment of the likely sensitivity to climate change for the key marine species in the southern Benguela system. Afr. J. Mar. Sci..

[39] Spencer PD, Hollowed AB, Sigler MF, Hermann AJ, Nelson MW (2019). Trait-based climate vulnerability assessments in data-rich systems: An application to eastern Bering Sea fish and invertebrate stocks. Glob. Change Biol..

[40] IPCC. *Climate Change 2014: Impacts, Adaptation, and Vulnerability. Part A: Global and Sectoral Aspects. Contribution of Working Group II to the Fifth Assessment Report of the Intergovernmental Panel on Climate Change* (eds. Field, C.B. *et al.*) 1132 (Cambridge University Press, 2014).

[41] Beever EA (2016). Improving conservation outcomes with a new paradigm for understanding species’ fundamental and realized adaptive capacity. Conserv. Lett..

[42] Fortini L, Schubert O (2017). Beyond exposure, sensitivity and adaptive capacity: A response based ecological framework to assess species climate change vulnerability. Clim. Change Resp..

[43] Gardali T, Seavy NE, DiGaudio RT, Comrack LA (2012). A climate change vulnerability assessment of California’s at-risk birds. PLoS ONE.

[44] Thompson, L. M., Staudinger, M. D. & Carter, S.L. *Summarizing components of U.S. Department of the Interior vulnerability Assessments to Focus Climate Adaptation Planning* (U.S. Geological Survey, Reston, 2015).

[45] Bueno-Pardo J (2021). Climate change vulnerability assessment of the main marine commercial fish and invertebrates of Portugal. Sci. Rep..

[46] Pilgrim JM, Fang X, Stefan HG (1998). Stream temperature correlations with air temperatures in Minnesota: Implications for climate warming. J. Am. Water Resour. Assoc..

[47] Hare JA, Able K (2007). Mechanistic links between climate and fisheries along the east coast of the United States: Explaining population outbursts of Atlantic croaker (*Micropogonias undulatus*). Fish. Oceanogr..

[48] IPCC. Summary for Policymakers. In *Global Warming of 1.5 °C. An IPCC Special Report on the Impacts of Global Warming of 1.5 °C Above Pre-industrial Levels and Related Global Greenhouse Gas Emission Pathways, in the Context of Strengthening the Global Response to the Threat of Climate Change, Sustainable Development, and Efforts to Eradicate Poverty* (eds. Masson-Delmotte, V. *et al.*) 32 (World Meteorological Organization, 2018).

[49] Morrison, W. E. *et al.**Methodology for Assessing the Vulnerability of Marine Fish and Shellfish Species to a Changing Climate*. U.S. Department of Commerce, NOAA. NOAA Technical Memorandum. NMFS-OSF-3: pp 48. http://www.st.nmfs.noaa.gov/Assets/ecosystems/climate/documents/TM%20OSF3.pdf (2015).

[50] Fulton EA (2011). Interesting times: Winners, losers, and system shifts under climate change around Australia. ICES J. Mar. Sci..

[51] Pethybridge HR (2020). Contrasting futures for Australia's fisheries stocks under IPCC RCP8.5 emissions—A multi-ecosystem model approach. Front. Mar. Sci..

[52] Franco AC, Gruber N, Frolicher TL, Kropuenske Artman L (2018). Contrasting impact of future CO_2_ emission scenarios on the extent of CaCO_3_ mineral undersaturation in the Humboldt Current System. J. Geophys. Res. Oceans.

[53] Kroeker KJ (2013). Impacts of ocean acidification on marine organisms: Quantifying sensitivities and interaction with warming. Glob. Change Biol..

[54] Ramajo L (2016). Biomineralization changes with food supply confer juvenile scallops (*Argopecten purpuratus*) resistance to ocean acidification. Glob. Change Biol..

[55] Kluger LC, Kochalski S, Aguirre-Velarde A, Vivar I, Wolff M (2019). Coping with abrupt environmental change: The impact of the coastal El Niño 2017 on artisanal fisheries and mariculture in North Peru. ICES J. Mar. Sci..

[56] Bertrand A, Segura M, Gutiérrez M, Vásquez L (2004). From small-scale habitat loopholes to decadal cycles: A habitat-based hypothesis explaining fluctuation in pelagic fish populations off Peru. Fish. Fish..

[57] Gutiérrez, D. *et al.* Fortalecimiento del conocimiento actual sobre los impactos del cambio climático en la pesquería peruana. In *Avances del Perú en la adaptación al cambio climático del sector pesquero y del ecosistema marino-costero* (eds. Zavala, R. *et al.*) 125 (BID-MINAM-PRODUCE-IMARPE, 2019).

[58] Oliveros-Ramos, R., Ñiquen, M., Csirke, J. & Guevara-Carrasco, R. Chapter 14: Management of the Peruvian anchoveta (*Engraulis ringens*) fishery in the context of climate change. In *Adaptive Management of Fisheries in Response to Climate Change*. FAO Fisheries and Aquaculture Technical Paper No. 667 (eds. Bahri, T. *et al.*) (FAO, 2021). 10.4060/cb3095en

[59] Castillo R (2019). Anchovy distribution off Peru in relation to abiotic parameters: A 32-year time series from 1985 to 2017. Fish. Oceanogr..

[60] Brown CJ (2016). Ecological and methodological drivers of species' distribution and phenology responses to climate change. Glob. Change Biol..

[61] Ballón M, Wosnitza-Mendo C, Guevara-Carrasco R, Bertrand A (2008). The impact of overfishing and El Niño on the condition factor and reproductive success of Peruvian hake, *Merluccius gayi peruanus*. Prog. Oceanogr..

[62] Guevara-Carrasco R, Lleonart J (2008). Dynamics and fishery of the Peruvian hake: Between the nature and the man. J. Mar. Syst..

[63] Avadí A, Adrien R, Aramayo V, Fréon P (2018). Environmental assessment of the Peruvian industrial hake fishery with LCA. Int. J. Life Cycle Assess..

[64] Brochier T (2013). Climate change scenarios experiments predict a future reduction in small pelagic fish recruitment in the Humboldt Current system. Glob. Change Biol..

[65] Levin L (2002). Benthic processes on the Peru margin: A transect across the oxygen minimum zone during the 1997–1998 El Niño. Prog. Oceanogr..

[66] Ulloa O, Pantoja S (2009). The oxygen minimum zone of the eastern South Pacific. Deep Sea Res. Part II Top. Stud. Oceanogr..

[67] Bertrand A (2011). Oxygen: A fundamental property regulating pelagic ecosystem structure in the coastal Southeastern Tropical Pacific. PLoS ONE.

[68] Breitburg D (2018). Declining oxygen in the global ocean and coastal waters. Science.

[69] Pörtner HO (2010). Oxygen- and capacity-limitation of thermal tolerance: A matrix for integrating climate-related stressor effects in marine ecosystems. J. Exp. Biol..

[70] Brokordt K, Pérez H, Herrera C, Gallardo A (2015). Reproduction reduces HSP70 expression capacity in *Argopecten purpuratus* scallops subject to hypoxia and heat stress. Aquat. Biol..

[71] Rose, K. A. *et al.* Impacts of ocean deoxygenation on fisheries. In *Ocean Deoxygenation: Everyone’s Problem-Causes, Impacts, Consequences and Solutions* (eds. Laffoley, D., Baxter, J. M.) 519–544 (IUCN, 2019).

[72] Gutiérrez D (2008). Oxygenation episodes on the continental shelf of central Peru: Remote forcing and benthic ecosystem response. Prog. Oceanogr..

[73] Tarazona J, Salzwedel H, Arntz WE (1988). Positive effects of ‘‘El Niño” on macrozoobenthos inhabiting hypoxic areas of the Peruvian upwelling system. Oecologia.

[74] Yu W, Yi Q, Chen X, Chen Y (2016). Modelling the effects of climate variability on habitat suitability of jumbo flying squid, *Dosidicus gigas*, in the Southeast Pacific Ocean off Peru. ICES J. Mar. Sci..

[75] Mendo, J., Wolff, M., Carbajal, W., Gonzáles, I. & Badjeck, M. Manejo y explotación de los principales bancos naturales de concha de abanico (*Argopecten purpuratus*) en la costa Peruana. In *Estado actual del cultivo y manejo de moluscos bivalvos y su proyección futura: Factores que afectan su sustentabilidad en América Latina*. FAO Actas de Pesca y Acuicultura 12 (eds. Lovatelli, E., Farías, A., Uriarte, I.) 101–114 (FAO, 2008).

[76] De Silva, S.S. & Soto, D. Climate change and aquaculture: Potential impacts, adaptation and mitigation. In *Climate Change Implications for Fisheries and Aquaculture: Overview of Current Scientific Knowledge. FAO Fisheries and Aquaculture Technical Paper 530* (eds. Cochrane, K., De Young, C., Soto, D., Bahri, T.) 151–212 (FAO, 2009).

[77] Aragão G (2021). The importance of regional differences in vulnerability to climate change for demersal fisheries. ICES J. Mar. Sci..

[78] Woods PJ (2021). A review of adaptation options in fisheries management to support resilience and transition under socio-ecological change. ICES J. Mar. Sci..

[79] Pinsky ML (2018). Preparing ocean governance for species on the move. Science.

[80] Abusamah A, Shaffril HAM, Hamzah A, Abusamah B (2019). Factors affecting small-scale fishermen’s adaptation toward the impacts of climate change: Reflections from Malaysian fishers. SAGE Open.

[81] Zavala, R., Gonzales, N., Lazo, O. & Morales, R. Inclusión transversal del cambio climático en el manejo de zonas marino-costeras. In *Avances del Perú en la adaptación al cambio climático del sector pesquero y del ecosistema marino-costero* (eds. Zavala, R. *et al.*) 125 (BID-MINAM-PRODUCE-IMARPE, 2019).

[82] Macfadyen, G. & Allison, E. *Climate Change, Fisheries, Trade and Competitiveness: Understanding Impacts and Formulating Responses for Commonwealth Small States* 103 (Commonwealth Secretariat-Poseidon-WorldFish, London, 2009).

[83] Madin EMP (2012). Socio-economic and management implications of range-shifting species in marine systems. Glob. Environ. Change.

[84] Jara HJ (2020). Current and future socio-ecological vulnerability and adaptation of artisanal fisheries communities in Peru, the case of the Huaura province. Mar. Policy.

[85] Sherman K (2014). Toward ecosystem-based management (EBM) of the world's large marine ecosystems during climate change. Environ. Dev..

