# Author Correction: Climate vulnerability assessment of key fishery resources in the Northern Humboldt Current System

**DOI:** 10.1038/s41598-022-10919-0

**Published:** 2022-04-21

**Authors:** Jorge E. Ramos, Jorge Tam, Víctor Aramayo, Felipe A. Briceño, Ricardo Bandin, Betsy Buitron, Antonio Cuba, Ernesto Fernandez, Jorge Flores-Valiente, Emperatriz Gomez, Hans J. Jara, Miguel Ñiquen, Jesús Rujel, Carlos M. Salazar, Maria Sanjinez, Rafael I. León, Mark Nelson, Dimitri Gutiérrez, Gretta T. Pecl

**Affiliations:** 1 Institute for Marine and Antarctic Studies, University of Tasmania, Hobart, TAS Australia; 2 Falkland Islands Fisheries Department, Directorate of Natural Resources, Stanley, Falkland Islands; 3 Instituto del Mar del Perú, Callao, Lima, Peru; 4 Laboratorio de Ciencias del Mar, Facultad de Ciencias Y Filosofía, Centro de Investigación Para El Desarrollo Integral Y Sostenible (CIDIS), Universidad Peruana Cayetano Heredia, Lima, Peru; 5 Facultad de Ciencias Biológicas, Universidad Nacional Mayor de San Marcos, Lima, Peru; 6 Laboratorio de Ecofisiología de Crustáceos, Instituto de Acuicultura, Universidad Austral de Chile, Puerto Montt, Chile; 7 Sociedad Peruana de Derecho Ambiental, San Isidro, Lima, Peru; 8 ECS, in Support of NOAA Fisheries, Office of Science and Technology, Silver Spring, MD USA; 9 Centre for Marine Socioecology, University of Tasmania, Hobart, TAS Australia

Correction to: *Scientific Reports* 10.1038/s41598-022-08818-5, published online 21 March 2022

The Acknowledgements section in the original version of this Article was incomplete.

“Carlos Romero and Lucero Achaya provided logistic support during the workshops and during the assessment, respectively. Kelly Ortega-Cisneros provided suggestions on data analyses. GTP was supported by an Australian Research Council Future Fellowship. This study was supported with funds from the Inter-American Development Bank through the IMARPE-PRODUCE-MINAM Project “Adaptation to climate change of the fishery sector and marine-coastal ecosystem of Peru” (PE-G1001/PE-T1297) and from the Adaptation Fund through PROFONANPE (Fondo de Promoción de las Áreas Naturales Protegidas del Perú).”

now reads:

“Carlos Romero and Lucero Achaya provided logistic support during the workshops and during the assessment, respectively. Kelly Ortega-Cisneros provided suggestions on data analyses. GTP was supported by an Australian Research Council Future Fellowship. This study was financed by the Inter-American Development Bank through the Project “Adaptation of the fishery sector and coastal marine ecosystem of Peru to climate change” (PE-G1001/PE-T1297) executed by the Ministry of Environment and the Ministry of Production, in collaboration with the Peruvian Marine Research Institute. This study was also financed by the Adaptation Fund Board through the project "Adaptation to the impacts of climate change on Peru's coastal marine ecosystem and fisheries" via the Fund for the Promotion of Peruvian Natural Protected Areas, executed by the Ministry of Production in collaboration with the Peruvian Marine Research Institute.”

The original Article has been corrected.

