# Supplementary material for: Climate vulnerability assessment of key fishery resources in the Northern Humboldt Current System
Source: Sci Rep. 2022 Mar 21;12:4800. doi: 10.1038/s41598-022-08818-5 (PMC8938481; doi:10.1038/s41598-022-08818-5)
Supplement: Supplementary file 1 — Supplementary Information. [file 41598_2022_8818_MOESM1_ESM.pdf]

Climate Vulnerability Assessment of key fishery resources in the Northern Humboldt Current System

(Supplementary information)

Jorge E. Ramos[1,2*], Jorge Tam[3,4], Víctor Aramayo[3,5], Felipe A. Briceño[6], Ricardo Bandin[7], Betsy Buitron[3], Antonio Cuba[3], Ernesto Fernandez[3], Jorge Flores-Valiente[4], Emperatriz Gomez[3], Hans J. Jara[3], Miguel Ñiquen[3], Jesús Rujel[3], Carlos M. Salazar[3], Maria Sanjinez[3], Rafael I. León[1], Mark Nelson[8], Dimitri Gutiérrez[3,4], Gretta T. Pecl[1,9]

[1] Institute for Marine and Antarctic Studies, University of Tasmania, Hobart, Tasmania, Australia

[2] Falkland Islands Fisheries Department, Directorate of Natural Resources, Stanley, Falkland Islands

[3] Instituto del Mar del Perú, Callao, Lima, Peru

[4] Laboratorio de Ciencias del Mar, Facultad de Ciencias y Filosofía, Centro de Investigación para el Desarrollo Integral y Sostenible (CIDIS), Universidad Peruana Cayetano Heredia, Lima, Peru

[5] Facultad de Ciencias Biológicas, Universidad Nacional Mayor de San Marcos, Lima, Peru

[6] Laboratorio de Ecofisiología de Crustáceos, Instituto de Acuicultura, Universidad Austral de Chile, Puerto Montt, Chile

[7] Sociedad Peruana de Derecho Ambiental, San Isidro, Lima, Peru

[8] ECS, in support of NOAA Fisheries, Office of Science and Technology, Silver Spring, Maryland, United States of America

[9] Centre for Marine Socioecology, University of Tasmania, Hobart, Tasmania, Australia

* Correspondence and requests for materials should be addressed to J.E.R. (jeramos@utas.edu.au)

Table of Contents

Methods S1. Species profiles 1

Benthic 1

1.1 Changos octopus – *Octopus mimus* 1

1.2 Chocolate rock shell – *Thaisella chocolata* 3

1.3 Peruvian calico scallop – *Argopecten purpuratus* 7

1.4 Purplish crab – *Platyxanthus orbigny* 10

1.5 Ribbed mussel – *Aulacomya atra* 14

Demersal 17

1.6 Corvina drum – *Cilus gilberti* 17

1.7 Fine flounder – *Paralichtys adspersus* 19

1.8 Flathead grey mullet – *Mugil cephalus* 22

1.9 Humpback smooth-hound – *Mustelus whitneyi* 25

1.10 Lorna drum – *Sciaena deliciosa* 29

1.11 Lumptail searobin – *Prionotus stephanophrys* 33

1.12 Patagonian squid – *Doryteuthis gahi* 36

1.13 Peruvian banded croaker – *Paralonchurus peruanus* 39

1.14 Peruvian hake – *Merluccius gayi peruanus* 43

1.15 Peruvian rock seabass – *Paralabrax humeralis* 46

1.16 Peruvian sea catfish – *Galeichthys peruvianus* 49

1.17 Peruvian weakfish – *Cynoscion analis* 53

Pelagic 57

1.18 Blue shark – *Prionace glauca* 57

1.19 Chilean Jack mackerel – *Trachurus murphyi* 59

1.20 Common dolphinfish – *Coryphaena hippurus* 62

1.21 Eastern Pacific bonito – *Sarda chiliensis chiliensis* 64

1.22 Jumbo flying squid – *Dosidicus gigas* 67

1.23 Mote sculpin – *Normanichthys crockeri* 72

1.24 Pacific chub mackerel – *Scomber japonicus* 74

1.25 Pacific sardine – *Sardinops sagax* 77
1.26 Peruvian anchovy – *Engraulis ringens* 80
1.27 Peruvian silverside – *Odontesthes regia* 82
1.28 Yellowfin tuna – *Thunnus albacares* 85
Methods S2. Climate exposure factors 89
Sea surface temperature 90
Sea Surface salinity 92
pH 94
Chlorophyll 96
Primary productivity 98
Precipitation 100
Air temperature 102
Sea bottom temperature 104
Sea bottom salinity 106
Methods S3. Climate exposure factor: Sea level rise 108
Figure S1 111
Figure S2 112
Figure S3 113
Table S1 114
Table S2 116

Methods S1. Species profiles

Benthic

**1.1 Changos octopus - *Octopus mimus***	
Sensitivity	
Abundance	
Fecundity – egg production (total fecundity)	Up to 20,000 eggs (Cortez *et al.* 1995); 432,000 eggs (Baltazar *et al.* 2000).
Recruitment period – successful recruitment event that sustains the abundance of the fishery	Due to its annual life cycle, recruitment to the fishery must occur before one year of life.
Average age at maturity	First maturity occurs at 12.5 cm of body dorsal length (Ishiyama *et al.* 1999). Octopuses generally have an annual life cycle, therefore it is assumed that this species reaches sexual maturity in maximum one year.
Generalist vs. specialist – food and habitat	Inhabits rocky and intertidal reefs at 0–30 m depth (Norman *et al.* 2013). In Pucusana, Peru it feeds on crustaceans, brachyurians, barnacles, gastropods and bivalve molluscs, echinoderms and fishes; canibalism also occurs (Cardoso *et al.* 2004).
Biomass	Not available.
Distribution	
Capacity for larval dispersal or larval duration – hatching to settlement (benthic species), hatching to yolk sac re-adsorption (pelagic species).	It is assumed that paralarvae may spend 35–60 days in the plankton, as per *O. vulgaris* (Villanueva 1995; Carrasco *et al.* 2006).
Capacity for adult/juvenile movement – lifetime range post-larval stage.	Not available.
Physiological tolerance – latitudinal coverage of adult species as a proxy of environmental tolerance.	Distributed from Tumbes, Peru to San Vicente, Chile. The distribution is also suggested from northern Peru to Valparaiso, Chile (Norman *et al.* 2013). Approximately 35° of latitudinal coverage.

	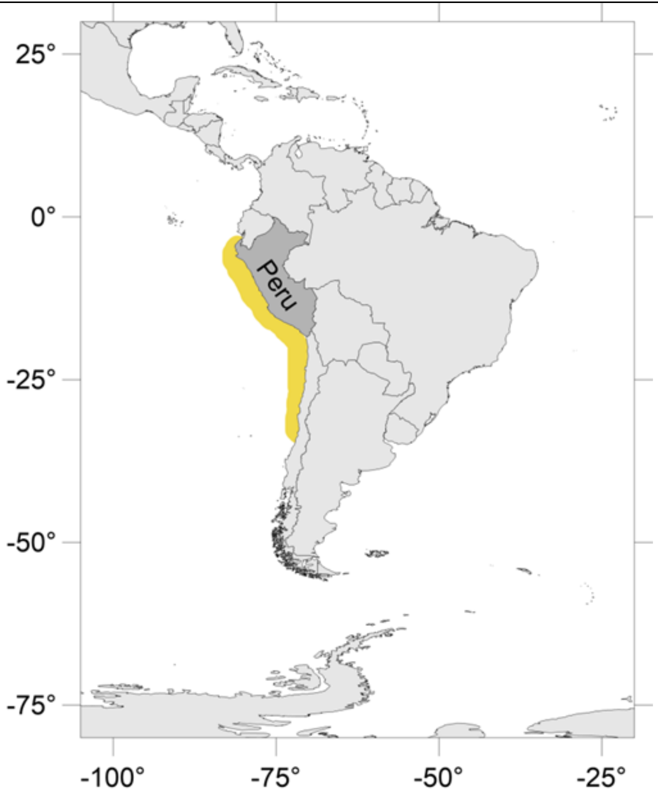
Spatial availability of unoccupied habitat for most critical life stage – ability to shift distributional range.	Approximately 17° of latitude may be available to the south of Peru.
Phenology	
Environmental variable as a phenological cue for spawning or breeding – cues include salinity, temperature, currents, and freshwater flows.	Spawning peaks appear to occur at higher temperatures (Cortez *et al.* 1995). Highest values of gonadosomatic index, from September to December, show that the period of sexual maturity and spawning coincides with the increase in sea surface temperature (Ishiyama *et al.* 1999).
Environmental variable as a phenological cue for settlement or metamorphosis	Cephalopods tend to have marked growth responses due to temperature changes (Forsythe & Van Heukelem 1987), with growth rates increasing at higher temperatures.
Temporal mismatches of life-cycle events – duration of spawning, breeding or moulting season.	Highest values of gonadosomatic index are from September to December (Ishiyama *et al.* 1999), about 4 months.
Migration (seasonal and spawning)	Not available.
Exposure	
Response to environmental variability	Low temperatures do not favor the sexual maturity of *O. mimus*, whereas the higher spawning peaks appear to occur

	at higher temperatures (Cortez *et al.* 1995). The highest values of the gonadosomatic index from September to December show that the period of sexual maturity and spawning of *O. mimus* coincides with the gradual increase in sea surface temperature (Ishiyama *et al.* 1999). In addition, cephalopods have marked growth responses due to temperature changes (Forsythe & Van Heukelem 1987). During warm events, i.e., during El Niño, catches of *O. mimus* increased in Peru (Cardoso *et al.* 2004).

References

Baltazar, P., Rodríguez, P., Rivera, W., & Valdivieso, V. Cultivo experimental de *Octopus mimus*, Gould 1852 en el Perú. *Rev. peru. biol.* **7**, 151–160 (2000).

Cardoso, F., Villegas, P., & Estrella, C. Observaciones sobre la biología de *Octopus mimus* (Cephalopoda: Octopoda) en la costa peruana. *Rev. peru. biol.* **11**, 45–50 (2004).

Carrasco, J.F., Arronte, J.C., & Rodríguez, C. Paralarval rearing of the common octopus, *Octopus vulgaris* (Cuvier). *Aquac. Res.* **37**, 1601–1605 (2006).

Cortez, T., Castro, B.G., & Guerra, A. Reproduction and condition of female *Octopus mimus* (Mollusca, Cephalopoda). *Mar. Biol.* **123**, 505–510 (1995).

Forsythe, J.W., & Van Heukelem, W.F. Growth. In: *Cephalopod life cycles, comparative reviews*, Vol 2. (ed. Boyle, P.R.) 135–156 (Academic Press, 1987).

Ishiyama, V., Shiga, B., & Talledo, C. Biología reproductiva del pulpo *Octopus mimus* (Mollusca: Cephalopoda) de la región de Matarani, Arequipa, Perú. *Rev. peru. biol.* **6**, 110–122 (1999).

Norman, M.D., Finn, J.K., & Hochberg, F.G. *Octopus mimus*. In: *Cephalopods of the World. An annotated and illustrated catalogue of cephalopod species known to date. Vol. 3. Octopods and vampire squids* (eds. Jereb, P., Roper, C.F.E., Norman, & M.D., Finn, J.K.) 54–55 (FAO, 2013).

Villanueva, R. Experimental rearing and growth of planktonic *Octopus vulgaris* from hatching to settlement. *Can. J. Fish. Aquat. Sci.* **52**, 2639–2650 (1995).

1.2 Chocolate rock shell – *Thaisella chocolata*

Sensitivity

Abundance	
Fecundity – egg production (total fecundity)	Deposits clusters of 100–150 egg capsules each, with an average of 2,600 eggs per capsule (Soledad-Romero *et al.* 2004), which is an approximate average of 325,000 eggs.

Recruitment period – successful recruitment event that sustains the abundance of the fishery	Likely consistent every 1–2 years.
Average age at maturity	The average size of first sexual maturity in females is 5.9–6.5 cm (Quiroz & Barriga 1997; Galindo *et al.* 1999). The maximum age estimated for this species is 2.5 years (Argüelles 2004), therefore it is assumed that it reaches sexual maturity before 2 years of age.
Generalist vs. specialist – food and habitat	It is a carnivorous and scavenger species (Barriga & Quiroz 1998) that feeds on clams, among other species (Avendaño *et al.* 1997). Inhabits the intertidal and subtidal zone on rocky bottoms (Galindo *et al.* 1999).
Biomass	The biomass has been estimated in some areas of Peru, e.g., the biomass was estimated at 61 tons around Mazorca Island (IMARPE 2015).
Distribution	
Capacity for larval dispersal or larval duration – hatching to settlement (benthic species), hatching to yolk sac re-adsorption (pelagic species).	Has a planktonic larval stage that is estimated to last up to 4 months (Soledad-Romero *et al.* 2004).
Capacity for adult/juvenile movement – lifetime range post-larval stage.	Not available but most likely limited.
Physiological tolerance – latitudinal coverage of adult species as a proxy of environmental tolerance.	Occurs from Ecuador to Valparaiso, Chile (Alamo & Valdivieso 1997); approximately 35° of latitudinal coverage.

	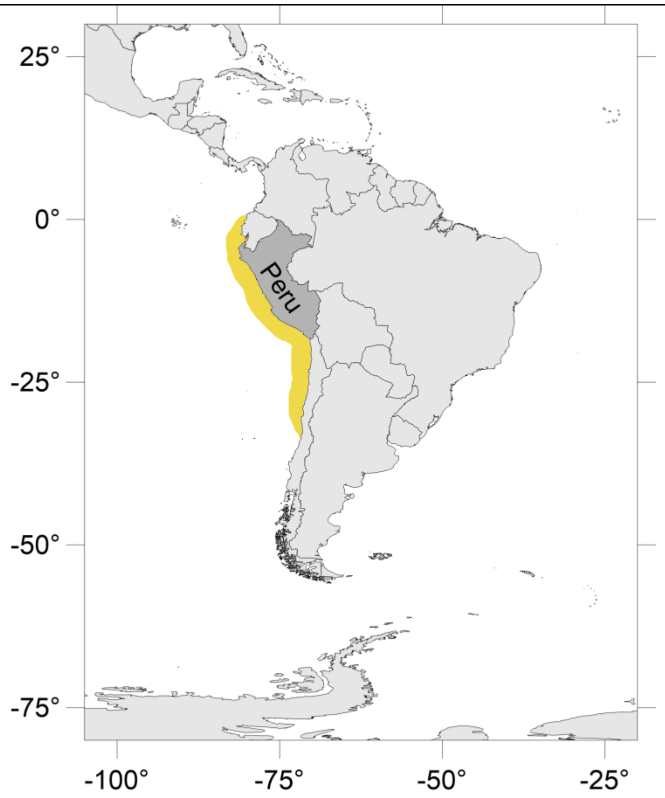
Spatial availability of unoccupied habitat for most critical life stage – ability to shift distributional range.	Approximately 15° of latitude may be available to the south of Peru.
Phenology	
Environmental variable as a phenological cue for spawning or breeding – cues include salinity, temperature, currents, and freshwater flows.	Gonadosomatic index and sea surface temperature in Moquegua and Tacna, Peru were found to be associated (Quiroz *et al.* 1996), where increasing sea surface temperature initiates the process of sexual maturation. Increase in temperature during El Niño 1997–1998 likely caused the extension of spawning season and recruitment during 1998 (Argüelles 2004).
Environmental variable as a phenological cue for settlement or metamorphosis	Not available.
Temporal mismatches of life-cycle events – duration of spawning, breeding or moulting season.	Two important annual spawning peaks, one peak is from May to June and the other from November to December in Moquegua and Tacna, Peru (Quiroz *et al.* 1996) although spawning individuals have also been observed throughout the year (Argüelles 2004). Each spawning peak lasts approximately 2 months.

Migration (seasonal and spawning)	In Moquegua and Tacna, Peru mature individuals carry out movements to aggregate during the reproductive season (Quiroz *et al.* 1996). In Antofagasta, Chile individuals aggregate for feeding (Avendaño *et al.* 1997).
Exposure	
Response to environmental variability	Quiroz *et al.* (1996) found a direct relationship between gonadosomatic index and sea surface temperature in Moquegua and Tacna, Peru, where increasing sea surface temperature initiates the process of sexual maturation. In Ica and northern Arequipa, El Niño 1997–1998 resulted in favorable conditions for the development of the snail (Galindo *et al.* 1999). Temperature changes during El Niño 1997–1998 likely caused the extension of spawning season and recruitment of *T. chocolata* during 1998 (Argüelles 2004). High densities of *T. chocolata* observed in the areas of Mal Nombre and Alfajes appear to be closely related to food availability (Argüelles 2004).
References	

Alamo, V., & Valdivieso, V. Lista sistemática de moluscos marinos del Perú (1997). In: Galindo, O., Segura, M., Flores, D. Prospección del caracol *Thais chocolata* en el litoral de Ica y norte de Arequipa, Mayo 1998. *Inf. Prog. Inst. Mar Perú* **111**, pp 13 (1999).

Argüelles, J.P. Cambios en la estructura y dinámica poblacional del caracol *Stramonita chocolata* (Duclos, 1832) asociados al evento El Niño 1997–98 en la zona del Callao, Perú. Dissertation, Universidad Nacional Mayor de San Marcos (2004).

Avendaño, M., Cantillanez, M., Olivares, A., & Oliva, M. Conducta reproductiva de *Thais chocolata* (Duclos, 1832) (Gastropoda: Thaididae) en La Rinconada, Antofagasta, Chile: causal de vulnerabilidad a la especie. *Rev. Biol. Mar. Oceanogr.* **32**, 177–187 (1997).

Barriga, E., & Quiroz, M. Prospección del recurso “caracol” (*Thais chocolata*) en el litoral de Moquegua y Tacna. Julio 1997. *Inf. Prog. Inst. Mar Perú* **90**, pp 16 (1998).

Galindo, O., Segura, M., & Flores, D. Prospección del caracol *Thais chocolata* en el litoral de Ica y norte de Arequipa, Mayo 1998. *Inf. Prog. Inst. Mar Perú* **111**, pp 13 (1999).

IMARPE. Anuario Científico Tecnológico IMARPE **15**. PRODUCE-IMARPE, pp 255 (2015).

Quiroz, M., & Barriga, E. Prospección del caracol *Thais chocolata* en el litoral de Moquegua y Tacna, Julio 1996. *Inf. Prog. Inst. Mar Perú* **58**, pp 16 (1997).

Quiroz, M., Barriga, E., & Rabí, M. Estado actual de la pesquería de los recursos tolina *Concholepas concholepas* y caracol *Thais chocolata* en el litoral de Moquegua y Tacna. *Inf. Prog. Inst. Mar Perú* **25**, pp 18 (1996).

Soledad-Romero, M., Gallardo, C.S., & Bellolio, G. Egg laying and embryonic-larval development in the snail *Thais* (*Stramonita*) *chocolata* (Duclos, 1832) with observations on its evolutionary relationships within the Muricidae. *Mar. Biol.* **145**, 681–692 (2004).

1.3 Peruvian calico scallop – *Argopecten purpuratus*

**Sensitivity**	
Abundance	
Fecundity – egg production (total fecundity)	1–40 million eggs (Bermudez-Corcuera *et al.* 2004).
Recruitment period – successful recruitment event that sustains the abundance of the fishery	Spawning occurs throughout the year with the highest spawning peaks in spring and summer (IMARPE 2014). Takes from 1 to 1.5 years to reach the commercial size (65 mm) (Mendo *et al.* 2008); hence it is assumed that recruitment events occur every 1–2 years.
Average age at maturity	Reaches sexual maturity at 10–12 months of age (Bermudez-Corcuera *et al.* 2004; IMARPE 2008)
Generalist vs. specialist – food and habitat	Filters phytoplankton (Bermudez-Corcuera *et al.* 2004). Inhabits protected areas with shells, rocky, sandy, sandy-muddy, and silty bottoms, or with algae. It is distributed from 3 to 60 m depth; the natural banks occur mainly between 10 and 20 m depth (Bermudez-Corcuera *et al.* 2004).
Biomass	The biomasses were estimated in < 10,000 t in each of the most productive scallop banks of Peru (Independencia Bay, Sechura Bay and Lobos de Tierra Island) (Mendo *et al.* 2008). The biomass was estimated at 1.5 t in Lobos de Tierra Island during September 2006 (Carbajal *et al.* 2006), which has increased in recent years. Sechura Bay had the greatest biomass from 2008 to 2012, with nearly 60,000 t in 2009 (Mendo *et al.* 2016).
Distribution	
Capacity for larval dispersal or larval duration – hatching to settlement (benthic species), hatching to yolk sac re-adsorption (pelagic species).	The planktonic larval stage lasts approximately 15 days (IMARPE 2008).

Capacity for adult/juvenile movement – lifetime range post-larval stage.	Adult bivalves do not appear to move great distances, for instance adult *Pecten novaezealandiae* in New Zealand move approximately 2 meters per month (Twist *et al.* 2016).
Physiological tolerance – latitudinal coverage of adult species as a proxy of environmental tolerance.	Occurs from Paita, Peru to Valparaiso, Chile (IMARPE 2008); approximately 28° of latitudinal coverage. 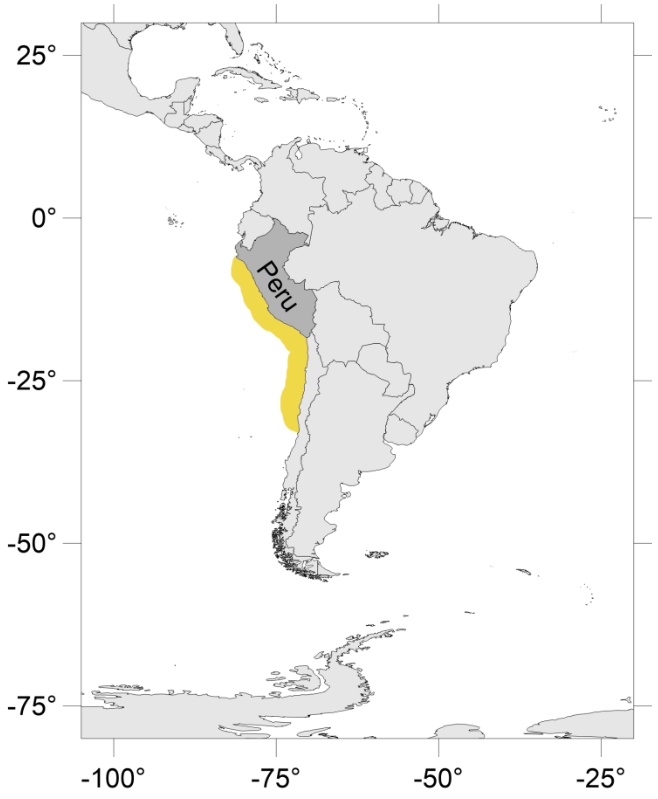
Spatial availability of unoccupied habitat for most critical life stage – ability to shift distributional range.	Approximately 15° of latitude may be available to the south of Peru.
Phenology	
Environmental variable as a phenological cue for spawning or breeding – cues include salinity, temperature, currents, and freshwater flows.	Low temperatures have negative effects on spawning and recruitment (Mendo *et al.* 2008). Mature and spawning individuals appear to be more vulnerable than juveniles to thermal stress and hypoxia (Brokordt *et al.* 2015). The decline in salinity due to river discharges may have caused massive mortality in the scallop bank at Tortugas Bay in 1998 (Mendo *et al.* 2008). Temperature, dissolved oxygen, currents, and turbidity were found to affect the gonadosomatic index (Cueto *et al.* 2014). Temperature and current velocity were highly associated with gonad weight variability (Cabrera & Mendo 2011).

Environmental variable as a phenological cue for settlement or metamorphosis	The peruvian calico scallop takes 1 to 1.5 years to reach commercial size; however, during El Niño events it only takes 6 to 8 months to reach commercial size (Mendo *et al.* 2008).
Temporal mismatches of life-cycle events – duration of spawning, breeding or moulting season.	Spawning occurs throughout the year with the highest spawning peaks in spring and summer (IMARPE 2014), between 2 and 6 months of duration.
Migration (seasonal and spawning)	Not available.
Exposure	
Response to environmental variability	The abundance of *A. purpuratus* in Peru has increased in some sites after El Niño events (Mendo *et al.* 2008). Oceanic warming and improvement of oxygen conditions near the bottom result in increasing growth rates and recruitment of this species in Pisco, as well as in the increase of carrying capacity of the bays. Low temperatures have negative effects on spawning and recruitment (Mendo *et al.* 2008). In Chile, this species showed a better physiological condition (based on fecundity, egg size, biochemical composition, and larval survival) at 15°C, and at decreasing temperatures starting at 19°C down to 15°C under controlled conditions (Martínez & Pérez 2003). Mature and spawning individuals appear to be more vulnerable than juveniles to thermal stress and hypoxia (Brokordt *et al.* 2015). The decline in salinity due to river discharges may have caused massive mortality in the scallop bank at Bahía de Tortugas in 1998 (Mendo *et al.* 2008). Temperature, dissolved oxygen, currents, and turbidity were found to affect the gonadosomatic index (Cueto *et al.* 2014), whereas temperature and current velocity were highly associated to gonad weight variability (Cabrera & Mendo 2011). Scallops take 1–1.5 years to reach commercial size; however, during El Niño events it only takes 6–8 months to reach commercial size (Mendo *et al.* 2008).
References	

Bermudez-Corcuera, P.I., Maidana-Cuadros, J.C., Aquino-Bravo, H., & Palomino-Ramos, A.R. Manual de cultivo suspendido de concha de abanico. PRODUCE-FONDEPES-AECI-PADESPA, pp 100 (2004).

Brokordt, K., Pérez, H., Herrera, C., & Gallardo, A. Reproduction reduces HSP70 expression capacity in *Argopecten purpuratus* scallops subject to hypoxia and heat stress. *Aquat. Biol.* **23**, 265–274 (2015).

Cabrera, P., & Mendo, J. Condición somática y reproductiva de la concha de abanico (*Argopecten purpuratus*) y su relación con variables ambientales, en la Bahía de Sechura, Piura. In: *Libro de Resúmenes del Seminario "Bases científicas y tecnológicas para incrementar la productividad del cultivo de concha de abanico en áreas de repoblamiento en la bahía de Sechura"*, Piura 11 noviembre 2010. Proyecto FINCYT – Contrato Nro.01-2009 (ed. Mendo, J.) 23–28 (2011).

Carbajal, W., de la Cruz, J., Ramírez, P., Taipe, A., & Bances, S. Evaluación poblacional del recurso *Argopecten purpuratus* "concha de abanico" en la isla Lobos de Tierra (18–23 Setiembre 2006). Informe técnico IMARPE. Callao, Perú, pp 13 (2006).

Cueto, R., Mendo, J., Argüelles, J., Flye-Sainte-Marie, J., Jean, F., & Aguirre, A. Environmental effects on the gonadal condition in the Peruvian scallop *Argopecten purpuratus* (L. 1819) Paracas Bay, Peru. Oral communications. In: *Book of Abstracts submitted to the IV Congress of Marine Sciences.* Las Palmas de Gran Canaria. Universidad de Las Palmas de Gran Canaria, 2014, pp 176. Congress of Marine Sciences, 4, Las Palmas de Gran Canaria (SPA), 2014/06/11-13.ISBN978-84-697-0471-4 (2014).

IMARPE. Acondicionamiento de reproductores y obtención de semillas de concha de abanico *Argopecten purpuratus* (Lamarck 1819), en un sistema controlado experimental en el puerto de Ilo. Laboratorio de Investigación de moluscos. Informe anual. Ilo, Perú, pp 37 (2008).

IMARPE. Anuario Científico Tecnológico IMARPE **14**. PRODUCE-IMARPE, pp 241 (2014).

Martínez, G., & Perez, H. Effect of different temperature regimes on reproductive conditioning in the scallop *Argopecten purpuratus*. *Aquaculture* **228**, 153–167 (2003).

Mendo, J., Wolff, M., Carbajal, W., Gonzáles, I., & Badjeck, M. Manejo y explotación de los principales bancos naturales de concha de abanico (*Argopecten purpuratus*) en la costa Peruana. In: *Estado actual del cultivo y manejo de moluscos bivalvos y su proyección futura: factores que afectan su sustentabilidad en América Latina.* Taller Técnico Regional de la FAO. 20–24 de agosto de 2007, Puerto Montt, Chile. FAO Actas de Pesca y Acuicultura. No. 12. (eds. Lovatelli, E., Farías, A., & Uriarte, I.) 101–114 (FAO, 2008).

Mendo, J., Wolff, M., Mendo, T., & Ysla, L. Chapter 28. Scallop fishery and culture in Peru. In: *Scallops: Biology, Ecology, Aquaculture, and Fisheries*, 3rd edn. (eds. Shumway, S.E., & Parsons, G.J.) 1089–1110 (Elsevier Science, 2016).

Twist, B.A., Rayment, W.J., & Hepburn, C.D. Movement patterns of adult scallops (*Pecten novaezealandiae*) within a customary fisheries reserve: Implications for fine scale spatial management. *Fish. Res.* **174**, 160–166 (2016).

1.4 Purplish crab – *Platyxanthus orbigny*

Sensitivity	
Abundance	

Fecundity – egg production (total fecundity)	Average fecundity was estimated at 105,462 eggs (Martínez-Segura 2016).
Recruitment period – successful recruitment event that sustains the abundance of the fishery	Recruitment is assumed to occur every 1–2 years.
Average age at maturity	Size at first maturity in females occurs at approximately 37 mm, and mean size at maturity was estimated at 61 mm (Martínez-Segura 2016). The average age of first sexual maturity is at 2 years (Mendoza 1992).
Generalist vs. specialist – food and habitat	It is an omnivorous species; juveniles feed on diatoms and adults prey upon barnacles and small mussels (Abarca 1967). Inhabits the eulittoral zone down to 50 m depth (Martínez-Segura 2016) in fine sand bottoms with sedimentary rocks (IMARPE 2015; Morales-Montañez & Prieto-Dueñas 2015).
Biomass	Using different models, biomass estimations ranged from 301 t to 739 t, and between 333 t to 758 t, with a maximum in 2001 and a minimum in 2006 in the area of Lambayeque (Torrejón-Magallanes 2011).
Distribution	
Capacity for larval dispersal or larval duration – hatching to settlement (benthic species), hatching to yolk sac re-adsorption (pelagic species).	There is a planktonic phase for this genus, as identified in *P. patagonicus* (Dellatorre *et al.* 2013). Metamorphosis to the crab stage usually occurs 25 days after hatching (Morales-Montañez & Prieto-Dueñas 2015).
Capacity for adult/juvenile movement – lifetime range post-larval stage.	Individuals move to deeper areas after they take the adult form (Morales-Montañez & Prieto-Dueñas 2015).
Physiological tolerance – latitudinal coverage of adult species as a proxy of environmental tolerance.	Occurs from Ecuador to San Antonio, Chile (Martínez-Segura 2016); approximately 33° of latitudinal coverage.

	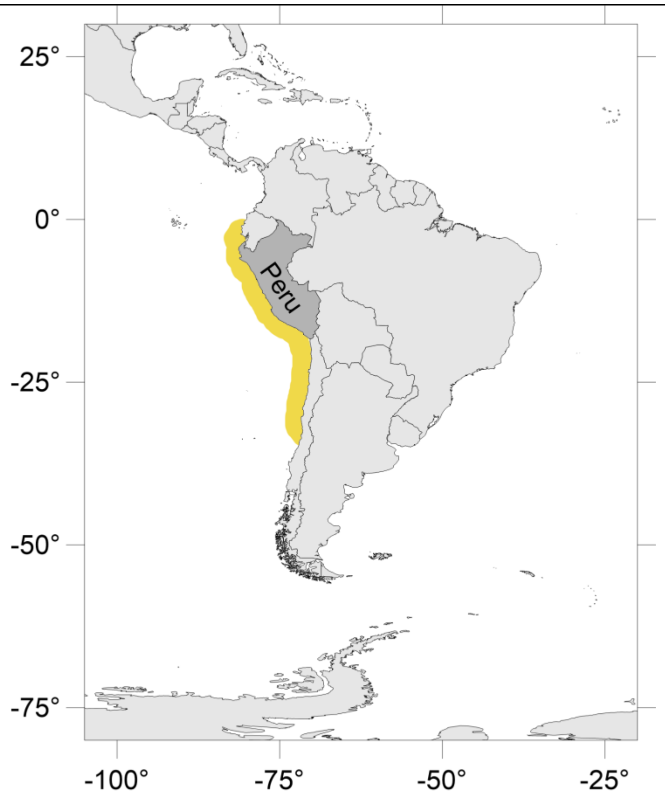
Spatial availability of unoccupied habitat for most critical life stage – ability to shift distributional range.	Approximately 15° of latitude may be available to the south of Peru.
Phenology	
Environmental variable as a phenological cue for spawning or breeding – cues include salinity, temperature, currents, and freshwater flows.	Not available.
Environmental variable as a phenological cue for settlement or metamorphosis	Young crabs molt during spring and summer and then take the adult form (Morales-Montañez & Prieto-Dueñas 2015).
Temporal mismatches of life-cycle events – duration of spawning, breeding or moulting season.	Mature females are present all year round and most frequently in February, June and November. Reproductive females were more common in March off Huanchaco, Peru and the highest gonadosomatic index occurred in May (Martínez-Segura 2016). The frequency of mature individuals was higher in December (79%), spawning individuals in February (54%) and post spawning in August (50%) 2009 off Lambayeque. Continuous reproductive activity is likely through the year and with peaks in some months (Llanos *et*

	al. 2009); spawning peaks have been detected in autumn and spring (IMARPE 2014). The duration of spawning is thus assumed to last > 4 months.
Migration (seasonal and spawning)	Individuals move to deeper areas after they take the adult form (Morales-Montañez & Prieto-Dueñas 2015).
Exposure	
Response to environmental variability	El Niño has negative effects on the purplish crab, which geographic distribution was reduced along the entire coast during El Niño 1997–1998 (Arntz & Valdivia 1985; Ñiquen & Bouchon 2004).
References	

Abarca, J. Contribución al conocimiento del cangrejo violáceo *Platyxanthus orbignyi.* Milne Edwards y Lucas 1843 en el Departamento de la Libertad (Perú). Dissertation, Universidad Nacional de Trujillo (1967).

Arntz, W.E., & Valdivia, E.D. Incidencia del fenómeno "El Niño" sobre los mariscos en el litoral Peruano. *Bol. Inst. Mar Perú,* 91–101 (1985).

Dellatorre, F.G. *et al.* Seasonal abundance and vertical distribution of crab larvae from northern Patagonia. *Mar. Biol. Res.* **10**, 37–50 (2013).

IMARPE. Anuario Científico Tecnológico IMARPE **14**. PRODUCE-IMARPE. pp 241 (2014).

IMARPE. Anuario Científico Tecnológico IMARPE **15**. PRODUCE-IMARPE. pp 255 (2015).

Llanos, J. *et al.* Investigaciones de IMARPE-Sede Lambayeque durante 2009. pp 89 (2009).

Martínez-Segura, L.N. Biología reproductiva de *Platyxanthus orbigny* "cangrejo violáceo" procedente de la caleta de Huanchaco – La Libertad durante el 2015. Dissertation, Universidad Nacional de Trujillo (2016).

Mendoza, A. Biología reproductiva del "cangrejo violáceo" *Platyxanthus orbigny* (Crustacea, Decapoda, Platyxanthidae) en el puerto de Pimentel, Lambayeque – Perú. Dissertation, Universidad Nacional Pedro Ruiz Gallo (1992).

Morales-Montañez, D., & Prieto-Dueñas, C.W. Eclosión de ovas del cangrejo violáceo *Platyxanthus orbigny* bajo condiciones de laboratorio, fecundada en su ambiente natural. Dissertation, Universidad Nacional José Faustino Sánchez Carrión (2015).

Ñiquen, M., & Bouchon, M. Impact of El Niño events on pelagic fisheries in Peruvian waters. *Deep Sea Res. Part II Top. Stud. Oceanogr.* **51**, 563–574 (2004).

Torrejón-Magallanes, E.J. Evaluación del estado poblacional del cangrejo violáceo *Platyxanthus orbigny* (Milne Edwards y Lucas, 1843) del área de Lambayeque por medio de un modelo dinámico de biomasa. Dissertation, Universidad Nacional Mayor de San Marcos (2011).

1.5 Ribbed mussel – *Aulacomya atra*

Sensitivity	
Abundance	
Fecundity – egg production (total fecundity)	Fecundity is high (García-Talledo 2015); in Africa, *A. atra* has three annual spawning events, where groups of individuals can produce 10,000,000,000 eggs/m^2/year (van Erkom Schurink & Griffiths 1991).
Recruitment period – successful recruitment event that sustains the abundance of the fishery	Spawning may occur every year but size at spawning may be reached in up to four years (IMARPE 2014). In addition, recruitment is favoured during La Niña (Tarazona *et al.* 2003). Threfore, recruitment may be occasional and variable.
Average age at maturity	In Chile, the minimum size of spawning individuals is 65 mm (Lozada 1968), a size that is reached at four years.
Generalist vs. specialist – food and habitat	Filters plankton, and it is also a detritivorous species (Osorio 1979; Garcia-Talledo 2015); sessile organism that is found in intertidal rocky areas down to 10 m depth (Uriarte 2008; García-Talledo 2015; Subsecretaría de pesca de Chile).
Biomass	The biomass has been estimated in 40 kg/m^2; during El Niño events the biomass has been estimated at 25 individuals/m^2 (Valle *et al.* 2002). The average monthly biomass in 2014 was 12,548 kg (García-Talledo 2015).
Distribution	
Capacity for larval dispersal or larval duration – hatching to settlement (benthic species), hatching to yolk sac re-adsorption (pelagic species).	It is inferred that the larval stage can last a few weeks; for example, the larval stage of *Choromytilus meridionalis* lasts 31–60 days, with a settlement peak at 35–50 days, while the larval stage of *Mytilus edulis* is 21–35 days (Bayne 1976; Kautsky 1982).
Capacity for adult/juvenile movement – lifetime range post-larval stage.	Not available; most likely limited.
Physiological tolerance – latitudinal coverage of adult species as a proxy of environmental tolerance.	Occurs in Peru and Chile, from Callao to the Beagle Channel, as well as in the Juan Fernández Archipelago (García-Talledo 2015); approximately 43° of latitudinal coverage.

	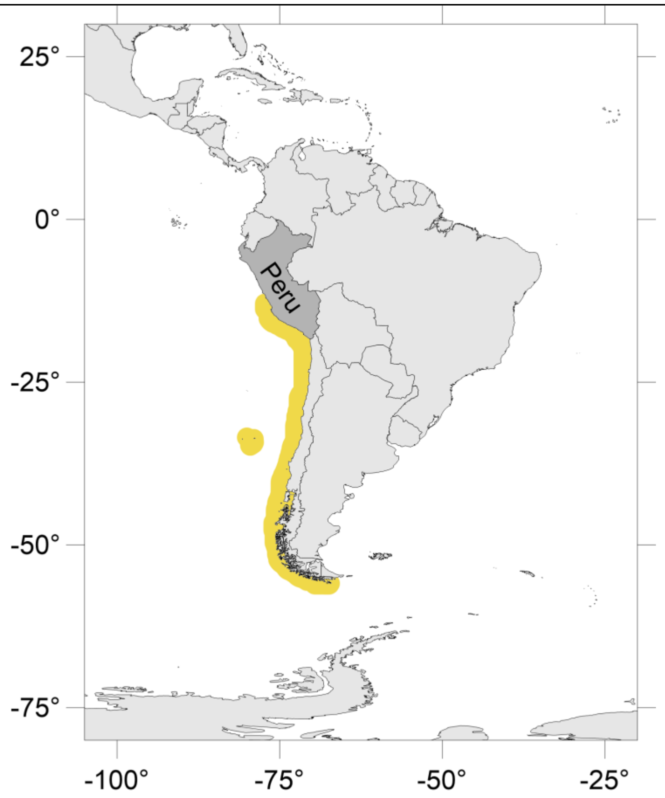
Spatial availability of unoccupied habitat for most critical life stage – ability to shift distributional range.	Approximately 37° of latitude may be available to the south of Peru.
Phenology	
Environmental variable as a phenological cue for spawning or breeding – cues include salinity, temperature, currents, and freshwater flows.	The recruitment of *A. atra* is favoured during La Niña (Tarazona *et al.* 2003).
Environmental variable as a phenological cue for settlement or metamorphosis	Pediveliger larvae of *Mytilus edulis* are able to delay their metamorphosis for up to 40 days at 10°C or for 2 days at 20°C if they do not find a favourable substrate for settlement (Bayne 1975).
Temporal mismatches of life-cycle events – duration of spawning, breeding or moulting season.	Spawning peaks in autumn and winter (IMARPE 2014); between 2 and 6 months of duration.
Migration (seasonal and spawning)	Not available.
Exposure	

Response to environmental variability	The recruitment of *A. atra* is favored during La Niña (Tarazona *et al.* 2003). The landing of this species has decreased during El Niño events. Pediveliger larvae of *Mytilus edulis* are able to delay their metamorphosis for up to 40 days at 10°C or for 2 days at 20°C if they do not find a favorable substrate for settlement (Bayne 1975).

References

Bayne, B.L. Reproduction in bivalve molluscs under environmental stress. In: Physiological ecology of estuarine organisms (ed. Vernberg, J.F.) 259–277 (University of South Carolina Press, 1975).

Bayne, B.L. The biology of mussel larvae (1976) In: van Erkom Schurink, C., Griffiths, C.L. A comparison of reproductive cycles and reproductive output in four southern African mussel species. *Mar. Ecol. Prog. Ser.* **76**, 123–134 (1991).

García-Talledo, E.G. Determinación de la mortalidad total del choro (*Aulacomya ater*) en la región Lima Callao. Informe final. Universidad Nacional del Callao. Callao, Perú. pp 34 (2015).

IMARPE. Anuario Científico Tecnológico IMARPE **14**. PRODUCE-IMARPE. pp 241 (2014).

Kautsky, N. Quantitative studies on gonad cycle, fecundity, reproductive output and recruitment in a Baltic *Mytilus edulis* population. *Mar. Biol.* **68**, 143–160 (1982).

Lozada, E. Contribución al estudio de la cholga (*Aulacomya ater*) en Putemún. *Biol. pesq. Chile* **3**, 3–38 (1968).

Osorio, C. Moluscos marinos de importancia económica en Chile. *Biol. pesq. Chile* **11**, 3–47 (1979).

Subsecretaría de pesca de Chile. Informe técnico (R.PESQ.) Nº 10. Cierre de Registro Pesquero Artesanal de Recursos Bentónicos en la II Región. Subsecretaría de pesca. Gobierno de Chile. pp 28.

Tarazona, J., Gutiérrez, D., Paredes, C., & Indacochea, A. Overview and challenges of marine biodiversity research in Perú. *Gayana* **67**, 206–231 (2003).

Uriarte, I. Estado actual del cultivo de moluscos bivalvos en Chile. In: *Estado actual del cultivo y manejo de moluscos bivalvos y su proyección futura: factores que afectan su sustentabilidad en América Latina*. Taller Técnico Regional de la FAO. 20–24 de agosto de 2007, Puerto Montt, Chile. *FAO Actas de Pesca y Acuicultura* 12. Roma. FAO (eds. Lovatelli, A., Farías, A., & Uriarte, I.) 61–75 (FAO, 2008).

Valle, S., Tarazona, J., Indacochea, A., Ramos, E., & Serrano, W. Variabilidad inducida por el ciclo del ENOS en la Densidad Poblacional de algunos invertebrados bentónicos de bahía Independencia, Pisco-Perú. In: *Bases Ecológicas y Socioeconómicas para el Manejo de los Recursos Vivos de la Reserva Nacional de Paracas*. *Memorias I Jornada Científica* (eds. Mendo, J., & Wolff, M.) 68–76 (2002).

van Erkom Schurink, C., & Griffiths, C.L. A comparison of reproductive cycles and reproductive output in four southern African mussel species. *Mar. Ecol. Prog. Ser.* **76**, 123–134 (1991).

Demersal

1.6 Corvina drum – *Cilus gilberti*	
Sensitivity	
Abundance	
Fecundity – egg production (total fecundity)	In controlled environments, corvina species produce between 30,000 and 350,000 eggs/kg (Cárdenas 2012).
Recruitment period – successful recruitment event that sustains the abundance of the fishery	Not available.
Average age at maturity	The population turnover is every 2–5 years (Chao & Robertson 2010), therefore it is estimated that the age at maturity is reached at about two years of age.
Generalist vs. specialist – food and habitat	It's a demersal species that feeds on crustaceans and small fishes; inhabits sandy bottoms near beaches at approximately 50 m depth in temperate waters (Mejía *et al.* 1970).
Biomass	No biomass estimates were found for the area of study. According to the IUCN Red List this species is in the category UNCERTAIN (Chao & Robertson 2010), which means that there are not enough data.
Distribution	
Capacity for larval dispersal or larval duration – hatching to settlement (benthic species), hatching to yolk sac re-adsorption (pelagic species).	The larval stage is likely to be part of the plankton for about 30–50 days, as per *Micropogonias furnieri* (Sciaenidae) in the estuary of the Rio de la Plata, Argentina (Braverman 2011).
Capacity for adult/juvenile movement – lifetime range post-larval stage.	Not available.
Physiological tolerance – latitudinal coverage of adult species as a proxy of environmental tolerance.	It's endemic to the eastern Pacific and is distributed from northern Peru to Chile, including the Galapagos Islands (Chao & Robertson 2010); approximately 60° of latitudinal coverage.

	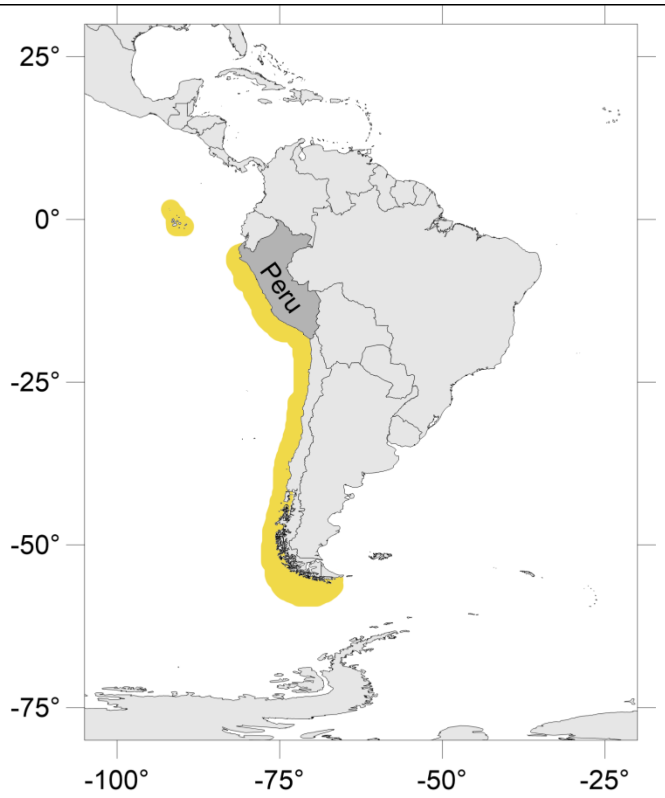
Spatial availability of unoccupied habitat for most critical life stage – ability to shift distributional range.	Approximately 41° of latitude may be available to the south of Peru.
Phenology	
Environmental variable as a phenological cue for spawning or breeding – cues include salinity, temperature, currents, and freshwater flows.	Not available.
Environmental variable as a phenological cue for settlement or metamorphosis	Not available.
Temporal mismatches of life-cycle events – duration of spawning, breeding or moulting season.	Not available.
Migration (seasonal and spawning)	Not available.
Exposure	
Response to environmental variability	Demersal fish inhabit the continental shelf and are associated with the Cronwell Subsurface Countercurrent,

	which oscillates intra and interannually. As a consequence, the habitat of these species also varies in size intra and interannually. In summer the habitat expands and in winter it is reduced, which results in lower and higher densities respectively. During El Niño events, the demersal system would behave as in summer conditions, depending on the intensity and duration of El Niño event. With El Niño, the diversity of the demersal system increases due to the incorporation of a larger number of species that move from north to south, from the coast to greater depths and from the pelagic system to the bottom (Velez *et al.* 1988). The corvina drum is a species that inhabits temperate waters; therefore, it is expected to be affected by oceanic warming associated with climate change. During El Niño events it has been affected in the northern area of its range of distribution (Chao & Robertson 2010).

References

Braverman, M.S. Life history temprana de la corvina rubia (*Micropogonias furnieri*, Scianidae) en el estuario del Río de la Plata. Dissertation, Universidad de Buenos Aires (2011).

Cárdenas, S. Biología y acuicultura de corvinas en el mundo. *Rev. AquaTIC* ***37***, 1–13 (2012).

Chao, L., & Robertson, R. *Cilus gilberti*. The IUCN Red List of Threatened Species 2010: e.T183478A8120318. Available at: http://dx.doi.org/10.2305/IUCN.UK.2010-3.RLTS.T183478A8120318.en. (2010).

Mejía, J., Samamé, M., & Pastor, A. Información básica de los principales peces de consumo. *Instituto del Mar, Serie de Informes Especiales IM–62*. pp 29 (1970).

Velez, J., Espino, M., Zeballos, J. Variación de la ictiofauna demersal frente al Perú entre 1981 y 1987. *Memorias del 2do Congreso Latinoeamericano sobre Ciencias del Mar* (COLACMAR), 17–21 Agosto de 1987, Lima, Perú. Tomo I: 203–212 (1988).

1.7 Fine flounder – *Paralichtys adspersus*

Sensitiivity

Abundance	
Fecundity – egg production (total fecundity)	This species has a total fecundity of 2,125,000 eggs/individual; the relative fecundity is 1,500 eggs/gram of fish (Angeles & Mendo 2005).

Recruitment period – successful recruitment event that sustains the abundance of the fishery	Recruitment is likely consistent every 1–2 years but may also be occasional and variable.
Average age at maturity	Size at 50% sexual maturity in females is 60.4 cm in length, which corresponds to approximately 6 years of age (Samamé & Castañeda 1999).
Generalist vs. specialist – food and habitat	Feeds on fishes, usually teleosts, as well as on cephalopods and other prey (Samamé & Castañeda 1999). Inhabits sandy-muddy or muddy bottoms (Samamé *et al.* 1985) in estuaries and mangroves at maximum depths of 35 m (Nielsen *et al.* 2010).
Biomass	According to the IUCN Red List this species falls in the category LEAST CONCERN (LC) (Nielsen *et al.* 2010), which means that it's abundant and widely distributed.
Distribution	
Capacity for larval dispersal or larval duration – hatching to settlement (benthic species), hatching to yolk sac re-adsorption (pelagic species).	Has a relatively short larval period (Herzka *et al.* 2009); the duration is not specified.
Capacity for adult/juvenile movement – lifetime range post-larval stage.	Flatfishes have limited movement capacity; for instance juveniles of *P. californicus* in Mexico are displaced from hundreds of meters to several kilometers as part of the estuarine migration process (Herzka *et al.* 2009).
Physiological tolerance – latitudinal coverage of adult species as a proxy of environmental tolerance.	Occurs in the South-east Pacific from Paita, Peru to Lota, Chile and Isla Juan Fernández (Samamé & Castañeda 1999), although its distribution has also been suggested to Ecuador (Nielsen *et al.* 2010); approximately 59° of latitudinal coverage.

	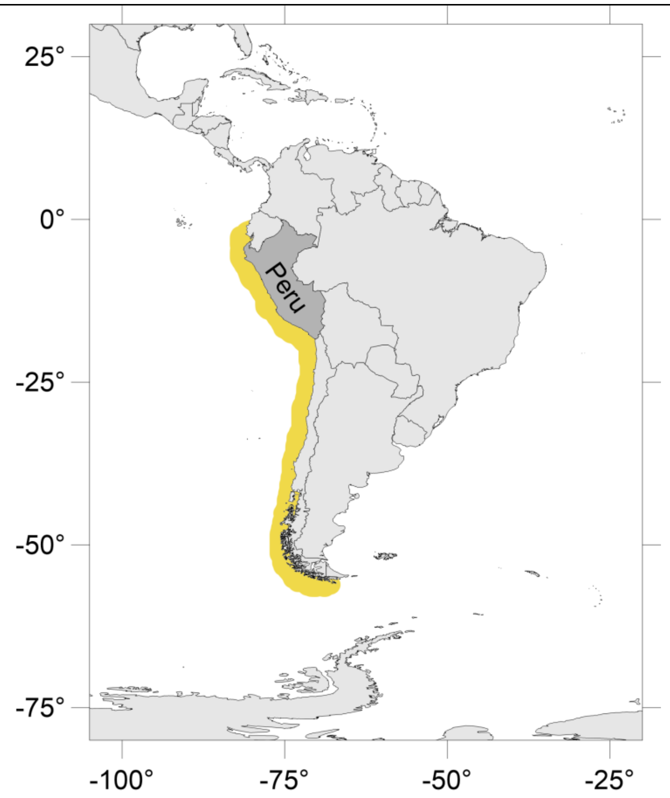
Spatial availability of unoccupied habitat for most critical life stage – ability to shift distributional range.	Approximately 41° of latitude may be available to the south of Peru.
Phenology	
Environmental variable as a phenological cue for spawning or breeding – cues include salinity, temperature, currents, and freshwater flows.	Temperature seems to play an important role in the spawning season. Spawning usually occurs from October to February (spring-summer) but during warm events such as El Niño 1997–1998 spawning peaks were recorded months after the usual season, i.e., during July 1997 (Samamé & Castañeda 1999).
Environmental variable as a phenological cue for settlement or metamorphosis	Not available.
Temporal mismatches of life-cycle events – duration of spawning, breeding or moulting season.	Spawns from October to February, during spring and summer (Samamé & Castañeda 1999); the duration of spawning is therefore assumed to be > 4 months.
Migration (seasonal and spawning)	Migrates to 200 m depth with changes in water masses and mainly with El Niño events (Samamé & Castañeda 1999), possibly due to the increase in temperature.

Exposure	
Response to environmental variability	The flatfish migrates to 200 m depth with changes in water masses and mainly with El Niño events (Sasamé & Castañeda 1999), possibly due to the increase in temperature. Its change in vertical distribution may also result in changes in abundance or availability to the fishery, therefore the landing of this species has decreased during El Niño events. Temperature also seems to play an important role in the spawning season. Spawning usually occurs from October to February (spring-summer) but during warm events such as El Niño 1997–1998 spawning peaks were recorded months after the usual season, i.e. during July 1997 (Samamé & Castañeda 1999).

References

Angeles, B., & Mendo, J. Crecimiento, fecundidad y diferenciación sexual del lenguado *Paralichthys adspersus* (Steindachner) de la costa central del Perú. *Ecol. Apl.* **4**, 115–112 (2005).

Herzka, S.Z., Griffiths, R., Fodrie, F.J., & McCarthy, I.D. Short-term size-specific distribution and movement patterns of juvenile flatfish in a Pacific estuary derived through length-frequency and mark-recapture data. *Cienc. Mar.* **35**, 41–57 (2009).

Nielsen, J.G., Munroe, T., Tyler, J., & Bussing, W. *Paralichthys adspersus*. The IUCN Red List of Threatened Species 2010: e.T183528A8129027. Available at: http://dx.doi.org/10.2305/IUCN.UK.2010-3.RLTS.T183528A8129027.en (2010).

Samamé, M., Castillo, J., & Mendieta, A. Situación de las pesquerías demersales y los cambios durante El Niño. In: Arntz, W., Landa, A., & Tarazona, J. (eds.) El Niño, su impacto en la fauna marina. *Bol. Extr. Inst. Mar Perú*, 153–158 (1985).

Sasamé, M., & Castañeda, J. Biología y pesquería del lenguado *Paralichthys adspersus*, con especial referencia al área norte del litoral peruano, departamento de Lambayeque. *Bol. Inst. Mar Perú* **18**, 15–48 (1999).

1.8 Flathead grey mullet - *Mugil cephalus*

Sensitivity	
Abundance	
Fecundity – egg production (total fecundity)	0.8 to 2.6 million eggs (Froese & Pauly 2021).

Recruitment period – successful recruitment event that sustains the abundance of the fishery	With spawning occurring every year (Llanos *et al.* 2009) and sexual maturity reached at 2–4 years (Culquichicón *et al.* 2011), recruitment may be consistent every 1–2 years although it may be affected and therefore also occasional and variable.
Average age at maturity	Sexual maturity between 2 and 4 years of life (Culquichicón *et al.* 2011; Froese & Pauly 2021). Length at first maturity is 25 cm approximately (Llanos *et al.* 2009).
Generalist vs. specialist – food and habitat	Juveniles feed on zooplankton; in general the flathead grey mullet feeds on detritus, microalgae, small invertebrates and other benthic organisms (Kottelat & Freyhof 2012). In Callao, central Peru, it feeds on diatoms, dinoflagellates, tintinids, silicoflagellates, copepods, among other species of zooplankton (Blaskovic' *et al.* 2008). Pelagic, occurs in coastal waters, in estuaries and rivers, occasionally upstream, in hypersaline lagoons and environments on sandy or muddy bottoms, between 0 and 10 m depth in tropical, subtropical and temperate waters (Froese & Pauly 2021).
Biomass	According to the IUCN Red List this species is in the category LEAST CONCERN (LC) (Kottelat & Freyhof 2012), which means that it is abundant and widely distributed.
Distribution	
Capacity for larval dispersal or larval duration – hatching to settlement (benthic species), hatching to yolk sac re-adsorption (pelagic species).	Not available.
Capacity for adult/juvenile movement – lifetime range post-larval stage.	Not available.
Physiological tolerance – latitudinal coverage of adult species as a proxy of environmental tolerance.	Cosmopolitan species that is distributed in California, the Mexican Pacific, Central America, the coasts of Peru and northern Chile (Kottelat & Freyhof 2012); approximately 35° of latitudinal coverage to the south of Peru and also to the north of Peru.

	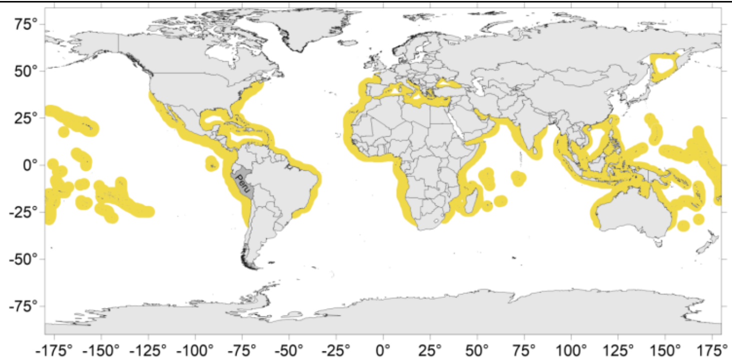
Spatial availability of unoccupied habitat for most critical life stage – ability to shift distributional range.	Approximately 17° of latitude may be available to the south of Peru.
Phenology	
Environmental variable as a phenological cue for spawning or breeding – cues include salinity, temperature, currents, and freshwater flows.	Positive thermal anomalies appear to have a negative effect in the main spawning peak. For instance, in 2009 the main spawning peak was less intense and it was surpassed by the secondary spawning peak (Llanos *et al.* 2009).
Environmental variable as a phenological cue for settlement or metamorphosis	Not available.
Temporal mismatches of life-cycle events – duration of spawning, breeding or moulting season.	Main spawning peak lasts approximately 3 months, between December and February. A secondary spawning peak lasts 2 months, between May and June (Llanos *et al.* 2009).
Migration (seasonal and spawning)	Spawning occurs at sea, juveniles approach the coast in December and remain in estuaries and coastal marine waters until they are approximately three years old (Oliver 1943).
Exposure	
Response to environmental variability	The largest catches of the flathead gray mullet were reported after the warm episode El Niño 1997–1998, whereas the presence of La Niña appears to be associated with the significant decline in landings (González-Ynope 2001). Warm waters during El Niño events along the Peruvian coast causes reduction of phytoplankton blooms and primary production. As a consequence, the typical species of the area move to other areas due to the lack of food, which may explain the landing fluctuations of coastal

	species such as the flathead gray mullet in the period 1996–1999 (González-Ynope 2001).<br><br>In 2009 when positive thermal anomalies occurred, the main spawning peak was less intense and it was surpassed by the secondary spawnings peaks (Llanos *et al.* 2009).

References

Blaskovic´, V., Espinoza, P., Fernández, C., Castillo, D., & Navarro, I. Espectro alimentario de las principales especies demersales de importancia comercial y sus relaciones intraespecíficas en el 2007. Informe técnico anual. Dirección de Investigaciones en peces demersales y litorales. Inst. Mar Perú. . pp 38 (2008).

Culquichicón, Z., Tresierra, A., Solano, A., & Atoche, D. Juvenación de *Paralonchurus peruanus*, *Mugil cephalus*, *Sciaena deliciosa* y *Ethmidium maculatum* en la Región La Libertad, durante el 2011. *Rebiol* **31**, pp 9 (2011).

Froese, R., & Pauly, D. (eds.) FishBase. World Wide Web electronic publication. Available at: https://www.fishbase.se/summary/Mugil-cephalus.html (2021).

González-Ynope, A. Contribución al conocimiento pesquero y biológico de cinco peces costeros de importancia comercial en el Perú: Cabinza, lisa, lorna, machete y pejerrey. Periodo 1996–2000. *Inf. Prog. Inst. Mar Perú* **195**, pp 46 (2001).

Kottelat, M., & Freyhof, J. *Mugil cephalus*. The IUCN Red List of Threatened Species 2012: e.T135567A515308. Available at: http://dx.doi.org/10.2305/IUCN.UK.2012.RLTS.T135567A515308.en (2012).

Llanos, J., *et al.* Investigaciones de IMARPE-Sede Lambayeque durante 2009. pp 89 (2009).

Oliver, S.C. Catálogo de los Peces Marinos del Litoral de Concepción y Arauco. *Bol. Soc. Biol. Concepc.* **17**, 75–126 (1943).

1.9 Humpback smooth-hound - *Mustelus whitneyi*

Sensitivity	
Abundance	
Fecundity – egg production (total fecundity)	Produces on average 9.5 offsprings (Samamé *et al.* 1985).
Recruitment period – successful recruitment event that sustains the abundance of the fishery	The recruitment to the fishery is assumed to be annual as for *M. antarcticus* (Walker 2010).

Average age at maturity	Sexual maturity is reached at approximately 74–87 cm (González-Pestana 2016), possibly at 9–10 years as in the case of *M. henlei* in the Gulf of California, Mexico (Méndez-Loeza 2008).
Generalist vs. specialist – food and habitat	Feeds on fish, crustaceans and molluscs (Llanos *et al.* 2009). Inhabits rocky, sandy and sandy-muddy bottoms around islands and is generally found at depths between 15 m and 200 m (Chirichigno & Cornejo 2001; Romero 2007).
Biomass	According to the IUCN Red List this species is in the category VULNERABLE (VU) (Romero 2007), which means that it is in danger of extinction.
Distribution	
Capacity for larval dispersal or larval duration – hatching to settlement (benthic species), hatching to yolk sac re-adsorption (pelagic species).	*Mustelus whitneyi* has no pelagic larval stage (EOL 2021).
Capacity for adult/juvenile movement – lifetime range post-larval stage.	The migratory capacity of this species is unknown; however juvenile *M. lenticulatus* in New Zealand were found to remain in areas of 2 to 7 km^2 (Francis 2013).
Physiological tolerance – latitudinal coverage of adult species as a proxy of environmental tolerance.	Distributed from Costa Rica, along the Peruvian coast and further south to Corral, Chile (Romero 2007); approximately 45° of latitudinal coverage to the south of Peru.

	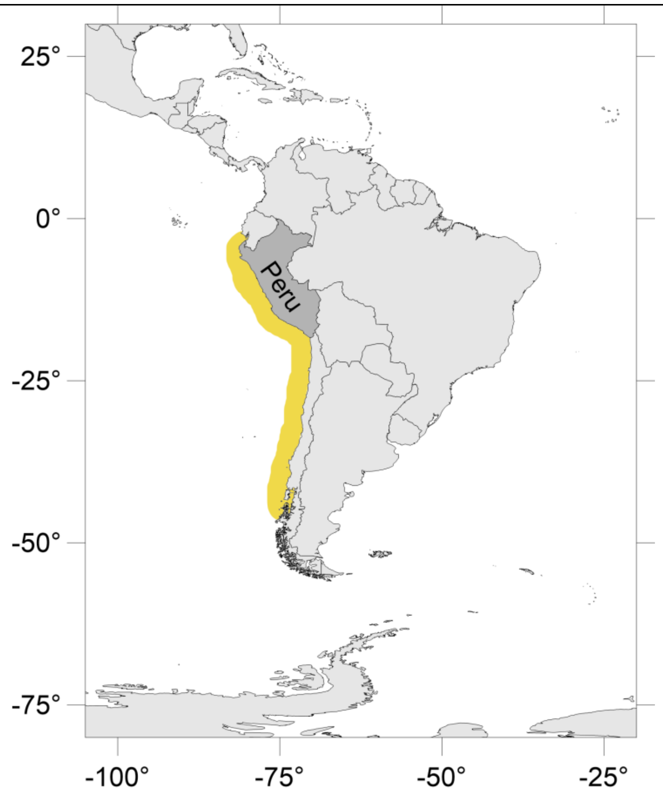
Spatial availability of unoccupied habitat for most critical life stage – ability to shift distributional range.	Approximately 30° of latitude may be available to the south of Peru.
Phenology	
Environmental variable as a phenological cue for spawning or breeding – cues include salinity, temperature, currents, and freshwater flows.	The spawning of the humpback smooth-hound decreases during El Niño events (Samamé *et al.* 1985).
Environmental variable as a phenological cue for settlement or metamorphosis	Not available.
Temporal mismatches of life-cycle events – duration of spawning, breeding or moulting season.	In the Northeast Atlantic, *M. asterias*, has a gestation period of 12 months and rests 12 months (Farrell *et al.* 2010).
Migration (seasonal and spawning)	Not available.
Exposure	
Response to environmental variability	Demersal fish inhabit the continental shelf and are associated with the Cronwell Subsurface Countercurrent,

	which oscillates intra and interannually. As a consequence, the habitat of these species also varies in size intra and interannually. In summer the habitat expands and in winter it is reduced, which results in lower and higher densities respectively. During El Niño events, the demersal system would behave as in summer conditions, depending on the intensity and duration of El Niño event. With El Niño, the diversity of the demersal system increases due to the incorporation of a larger number of species that move from north to south, from the coast to greater depths and from the pelagic system to the bottom (Velez *et al.* 1988). Changes in dissolved oxygen concentration in the water can result in changes in distribution and abundance (Espino 1990). During El Niño 1983, the largest sizes were present south of the main distribution area with the mode and average length being greater than in previous years (i.e., 1981) (Samamé *et al.* 1985). The spawning of the humpback smooth-hound also decreases during El Niño events (Samamé *et al.* 1985).

References

Chirichigno, N., Cornejo, R.M. Catálogo comentado de los peces marinos del Perú. *Inst. Mar Perú*. Callao, Perú (2001).

EOL - Encyclopedia of Life. Available at: https://eol.org/pages/46560004. (2021).

Espino, M. El Niño: Su impacto sobre los peces demersales del Perú. *Bol. Inst. Mar Perú* **14**, pp 28 (1990).

Farrell, E.D., Mariani, S., & Clarke, M.W. Reproductive biology of the starry smooth-hound shark *Mustelus asterias*: geographic variation and implications for sustainable exploitation. *J. Fish Biol.* **77**, 1505–1525 (2010).

Francis MP. Temporal and spatial patterns of habitat use by juveniles of a small coastal shark (*Mustelus lenticulatus*) in an estuarine nursery. *PLoS ONE* **8**, e57021 (2013).

González-Pestana, A. Preliminary results of the reproductive biology of the smooth-hound shark (*Mustelus whitneyi*). *V Encuentro Colombiano sobre Condrictios*. Colombia (2016).

Llanos, J. *et al.* Investigaciones de IMARPE-Sede Lambayeque durante 2009. pp 89 (2009).

Méndez-Loeza, I. Edad y crecimiento del cazón pardo, *Mustelus henlei* (Gill, 1863) en la región norte del Golfo de California. Dissertation, IPN-CICIMAR (2008).

Romero, M. *Mustelus whitneyi*. The IUCN Red List of Threatened Species 2007: e.T63129A12619394. http://dx.doi.org/10.2305/IUCN.UK.2007.RLTS.T63129A12619394.en (2007).

Samamé, M., Castillo, J., & Mendieta, A. Situación de las pesquerías demersales y los cambios durante El Niño. In: Arntz, W., Landa, A., & Tarazona, J. (eds.) El Niño, su impacto en la fauna marina. *Bol. Extr. Inst. Mar Perú*, 153–158 (1985).

Velez, J., Espino, M., & Zeballos, J. Variación de la ictiofauna demersal frente al Perú entre 1981 y 1987. *Memorias del 2do Congreso Latinoeamericano sobre Ciencias del Mar* (COLACMAR), 17–21 Agosto de 1987, Lima, Perú. Tomo I: 203–212 (1988).

Walker, T.I. Population biology and dynamics of the gummy shark (*Mustelus antarcticus*) harvested off southern Australia. Dissertation, The University of Melbourne (2010).

1.10 Lorna drum - *Sciaena deliciosa*

Sensitivity	
Abundance	
Fecundity – egg production (total fecundity)	Species of the family Sciaenidae produce 30,000–350,000 eggs/kg of body weight in controlled environments (Cárdenas 2012).
Recruitment period – successful recruitment event that sustains the abundance of the fishery	Likely to be consistent every 1–2 years.
Average age at maturity	The first maturity was estimated at 25.4 cm in length (Llanos *et al.* 2009), at the age of one year (Pérez-Huaripata 2013).
Generalist vs. specialist – food and habitat	This demersal species feeds on fish, crustaceans, polychaetes, molluscs and echinoderms (Llanos *et al.* 2009). It is found on the continental shelf on soft sandy or sandy-rocky bottoms down to 50 m depth (Chao & Espinosa 2010).
Biomass	According to the IUCN Red List this species is in the category LEAST CONCERN (LC) (Chao & Espinosa 2010), which means that it is abundant and widely distributed. However, Pérez-Huaripata (2013) indicates there was overfishing off Huacho from 2000 to 2011.
Distribution	
Capacity for larval dispersal or larval duration – hatching to settlement (benthic species), hatching to yolk sac re-adsorption (pelagic species).	It is assumed that the larval stage lasts 30–50 days in the plankton, as per *Micropogonias furnieri* (Scianidae) of the Río de la Plata estuary, Argentina (Braverman 2011).

Capacity for adult/juvenile movement – lifetime range post-larval stage.	Not available.
Physiological tolerance – latitudinal coverage of adult species as a proxy of environmental tolerance.	This species occurs in Ecuador, and from Puerto Pizarro, Peru to Corral, Chile (Chirichigno & Cornejo 2001); approximately 59° of latitudinal coverage. 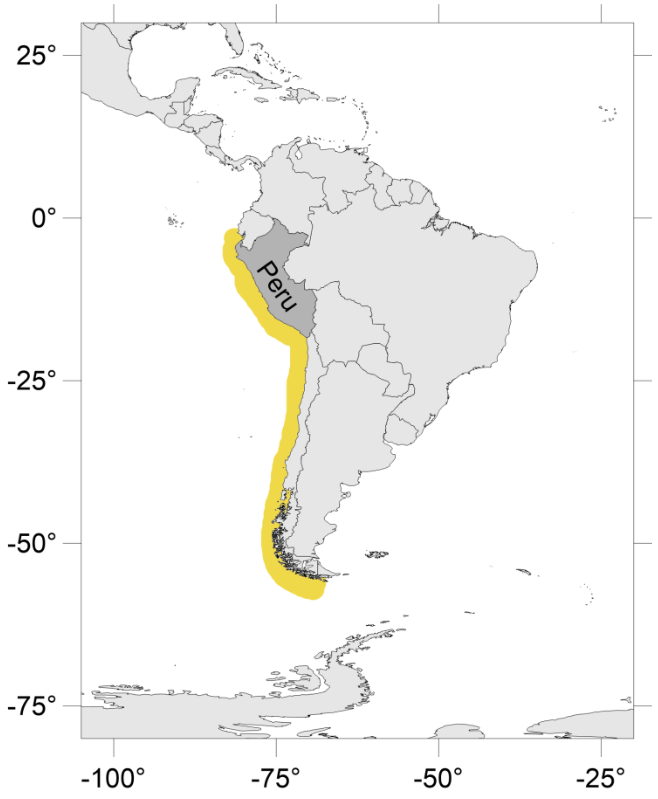
Spatial availability of unoccupied habitat for most critical life stage – ability to shift distributional range.	Approximately 41° of latitude may be available to the south of Peru.
Phenology	
Environmental variable as a phenological cue for spawning or breeding – cues include salinity, temperature, currents, and freshwater flows.	Spawning peaks are variable depending on the area and temperature (Wasiw 2000).
Environmental variable as a phenological cue for settlement or metamorphosis	Not available.

Temporal mismatches of life-cycle events – duration of spawning, breeding or moulting season.	In Peru, the spawning peaks vary depending on the area and can extend through 9 months. In Huacho the spawning peaks occur in winter and summer (González-Ynope 2001), although they have also been recorded in spring (Wasiw 2000). In Callao the spawning peaks occurred in winter and autumn, while in Chimbote and Pisco there were prolonged spawnings with the number of reproductive individuals decreasing from summer to winter, which is typical in tropical species (González-Ynope 2001). Reproductive peaks have also been detected in autumn, winter and spring in other studies (Estrella 1994; Estrella *et al.* 1998).
Migration (seasonal and spawning)	Not available.
Exposure	
Response to environmental variability	In general, demersal fish inhabit the continental shelf and are associated with the Cronwell Subsurface Countercurrent, which oscillates intra and interannually. As a consequence, the habitat of these species also varies in size intra and interannually. In summer the habitat expands and in winter it is reduced, which results in lower and higher densities respectively. During El Niño events, the demersal system would behave as in summer conditions, depending on the intensity and duration of El Niño event. With El Niño, the diversity of the demersal system increases due to the incorporation of a larger number of species that move from north to south, from the coast to greater depths and from the pelagic system to the bottom (Velez *et al.* 1988).<br><br>Temperature in particular seems to have a significant impact on the abundance of the lorna drum; for instance, the largest landing of this species in the period 1970–1999 was recorded in 1973 after El Niño 1972–1973. Significant landings have also been recorded during the development of cold episodes such as 1973–1974 (Estrella *et al.* 1998; González-Ynope 2001; Adams & Flores 2016). However, abundance peaks may be affected by the lower thermal limits, such as the cold event of 1999 in which catches declined after they had increased with El Niño 1997–1998 (Estrella *et al.* 1998; González-Ynope 2001).<br><br>The incursion of warm waters (e.g., during El Niño events) to the Peruvian coast causes the reduction of nutrients and primary production. As a consequence, the typical species of the area move due to the lack of food, which could explain

	in part the fluctuations in landings of coastal species such as lorna in the period 1996–1999 (González-Ynope 2001).<br><br>Spawning peaks also vary depending on the area and the cold and warm periods. The average size of sexual maturity also varies between one and another climatic period; in the cold period the average maturity size was 25.5 cm and in the warm period it was 26 cm (Wasiw 2000).

References

Adams, G.D., & Flores, D. Influencia de El Niño Oscilación del Sur en la disponibilidad y abundancia de recursos hidrobiológicos de la pesca artesanal en Ica, Perú. *Rev. Biol. Mar. Oceanogr.* **51**, 265–272 (2016).

Braverman, M.S. Life history temprana de la corvina rubia (*Micropogonias furnieri*, Scianidae) en el estuario del Río de la Plata. Dissertation. Universidad de Buenos Aires (2011).

Cárdenas, S. Biología y acuicultura de corvinas en el mundo. *Rev. AquaTIC **37***, 1–13 (2012).

Chao, L., & Espinosa, H. *Sciaena deliciosa*. The IUCN Red List of Threatened Species 2010: e.T183565A8135771. Available at: http://dx.doi.org/10.2305/IUCN.UK.2010-3.RLTS.T183565A8135771.en (2010).

Chirichigno, N., & Cornejo, R.M. Catálogo comentado de los peces marinos del Perú. *Inst. Mar Perú*. Callao, Perú (2001).

Estrella, C. Análisis poblacional de *Sciaena deliciosa* (Tschudii) "lorna" en el litoral peruano entre 1984–1992. Dissertation, Universidad Ricardo Palma (1994).

Estrella, C., Guevara-Carrasco, R., Palacios, J., Guardia, A., & Galán, J. Áreas de pesca de la flota artesanal de la caleta Santa Rosa, Chiclayo, Perú. 1996–1998. *Inf. Inst. Mar Perú* **142** (1998).

González-Ynope, A. Contribución al conocimiento pesquero y biológico de cinco peces costeros de importancia comercial en el Perú: Cabinza, lisa, lorna, machete y pejerrey. Periodo 1996–2000. *Inf. Prog. Inst. Mar Perú* **195**, pp 46 (2001).

Llanos, J. *et al.* Investigaciones de IMARPE-Sede Lambayeque durante 2009. pp 89 (2009).

Pérez-Huaripata, M.A. Análisis biológico-pesquero del recurso lorna (*Sciaena deliciosa*) en el puerto de Huacho, período 2000–2011. Dissertation, Universidad Nacional Agraria La Molina (2013).

Velez, J., Espino, M., Zeballos, J. Variación de la ictiofauna demersal frente al Perú entre 1981 y 1987. *Memorias del 2do Congreso Latinoeamericano sobre Ciencias del Mar* (COLACMAR), 17–21 Agosto de 1987, Lima, Perú. Tomo I: 203–212 (1988).

Wasiw, J. Aspectos biológico pesquero de la lorna (*Sciaena deliciosa*) y el machete (*Ethmidium maculatum*) en el área de Huacho durante un periodo frio y otro cálido. *Inf. Prog. Inst. Mar Perú* **128**, pp 19 (2000).

1.11 Lumptail searobin - *Prionotus stephanophrys*	
Sensitivity	
Abundance	
Fecundity – egg production (total fecundity)	There is no information on fecundity for this species but the partial fecundity of *P. ruscarius* in the Mexican Pacific is 10,400–118,200 eggs (Lucano-Ramírez *et al.* 2005).
Recruitment period – successful recruitment event that sustains the abundance of the fishery	Likely to be consistent every 1–2 years although it may be occasional and variable.
Average age at maturity	Size of maturity is reached at 20 cm in length, approximately at 3 years of age (Samamé & Fernández 2000).
Generalist vs. specialist – food and habitat	Feeds mainly on crustaceans, fish and cephalopods (Blaskovic' *et al.* 2008). Inhabits sandy or sandy-muddy bottoms down to 225 m depth (van der Heiden *et al.* 2010).
Biomass	In 2003, the cruises BIC Olaya, SNP2 and LIC IMARPE V 0303-04 carried out along Peru (from Tacna to Tumbes) estimated the biomass at 1,099 t (Castillo *et al.* 2009). According to the IUCN Red List this species is in the category LEAST CONCERN (LC) (van der Heiden *et al.* 2010), which means that it is abundant and widely distributed.
Distribution	
Capacity for larval dispersal or larval duration – hatching to settlement (benthic species), hatching to yolk sac re-adsorption (pelagic species).	The duration of the pelagic phase is unknown (EOL 2021).
Capacity for adult/juvenile movement – lifetime range post-larval stage.	Not available.
Physiological tolerance – latitudinal coverage of adult species as a proxy of environmental tolerance.	Distributed in the eastern Pacific from southern Baja California to southern Peru, including Malpelo and Galapagos Island (van der Heiden *et al.* 2010). There are records in northern Chile too (Samamé & Fernández 2000); approximately 45° of latitudinal coverage.

	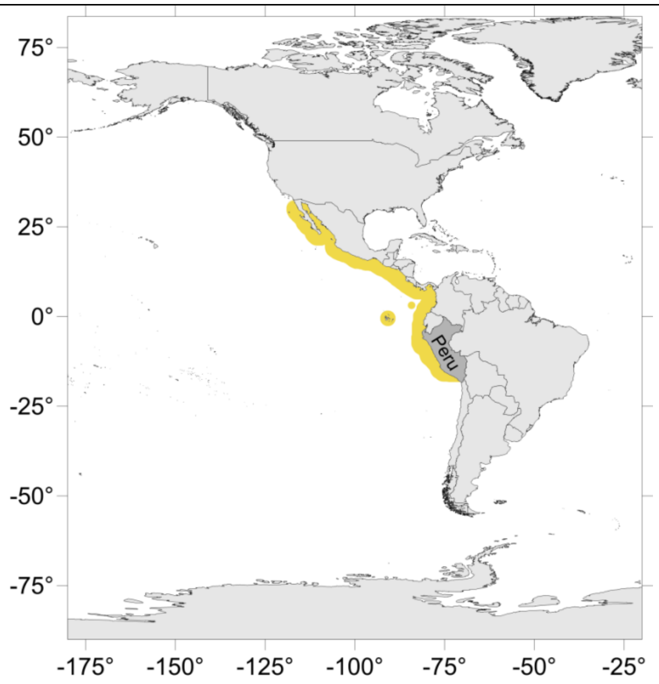
Spatial availability of unoccupied habitat for most critical life stage – ability to shift distributional range.	No unoccupied habitat availability to the south of Peru. Approximately 30° of latitude may be available to the north of Peru.
Phenology	
Environmental variable as a phenological cue for spawning or breeding – cues include salinity, temperature, currents, and freshwater flows.	With El Niño 1983, the spawning of *P. stephanophrys* occurred earlier in the year (Samamé *et al.* 1985).
Environmental variable as a phenological cue for settlement or metamorphosis	Not available.
Temporal mismatches of life-cycle events – duration of spawning, breeding or moulting season.	Mature individuals occur mainly in spring and summer; however, sexually mature individuals occur throughout the year except for winter (Samamé & Fernández 2000); the duration of spawning is thus assumed to last between 2 and 6 months.
Migration (seasonal and spawning)	Not available.
Exposure	
Response to environmental variability	Demersal species off Peru move latitudinally and longitudinally in response to environmental and biological conditions. In latitudinal movements, oceanographic factors

	such as the presence of surface sub-tropical waters, oxygen and salinity, as well as feeding and reproduction are crucial (Samamé & Fernández 2000). The lumptail searobin can be considered an indicator of warm waters, associated with movement of the Equatorial Front that occurs regularly every year or irregularly with the presence of El Niño events (Samamé & Fernández 2000). This species frequently occupies the area from the northern border of Peru to 10°S and down to the 220 m isobath. However, its distribution extends to 17°30'S during El Niño, where mainly juvenile and pelagic individuals are found (Samamé & Fernández 2000). Like other demersal species, the lumptail searobin tends to deepen during El Niño events (Espino 1990). The greatest concentrations of this species have been recorded at 14–16°C (Samamé & Fernández 2000). In contrast, it occurs at 16–18°C during El Niño events (Espino 1990). This species seems to be persistent to changes in salinity and oxygen (Samamé & Fernández 2000). However, as temperature influences the movement of demersal resources, dissolved oxygen is determinant in the vertical and horizontal distribution, and in the abundance of this species (Samamé & Fernández 2000). *Prionotus stephanophrys* is generally present between 0.5 mL/L and 1.5 mL/L of dissolved oxygen (Espino 1990; Samamé & Fernández 2000), whereas during El Niño it occurs between 1.25 mL/L and 2.5 mL/L of dissolved oxygen (Espino 1990). The deepening of the food from the coast may influence the distribution of the lumptail searobin as well (Samamé & Fernández 2000). The negative effects that changes in temperature have on the Peruvian hake seem to favor the increase in abundance of the lumptail searobin (Samamé *et al.* 1985).<br><br>Changes in size have been recorded probably due to El Niño events and the fishing pressure caused by trawlers (Samamé & Fernández 2000). With El Niño 1983, the spawning of *P. stephanophrys* occurred earlier in the year (Samamé *et al.* 1985).

References

Blaskovic´, V., Espinoza, P., Fernández, C., Castillo, D., & Navarro, I. Espectro alimentario de las principales especies demersales de importancia comercial y sus relaciones intraespecíficas en el 2007. Informe técnico anual. Dirección de Investigaciones en peces demersales y litorales. *Inst. Mar Perú*. pp 38 (2008).

Castillo, R., Segura, M., Gutiérrez, M., Ganoza, F., & Peraltilla, S. Distribución y biomasa de algunos recursos pelágicos en el mar peruano en verano 2003. *Inf. Inst. Mar Perú* **36** (2009).

EOL – Encyclopedia of Life. Available at: https://eol.org/pages/46568750 (2021).

Espino, M. El Niño: Su impacto sobre los peces demersales del Perú. *Bol. Inst. Mar Perú* **14**, pp 28 (1990).

Lucano-Ramírez, M.C., Ruiz-Ramírez, S., & Rojo-Vázquez, J.A. Biología reproductiva de *Prionotus ruscarius* (Pisces: Triglidae) en las costas de Jalisco y Colima, México. *RDU* **6**. pp 13 (2005).

Samamé, M., Castillo, J., & Mendieta, A. Situación de las pesquerías demersales y los cambios durante El Niño. In: Arntz, W., Landa, A., & Tarazona, J. (eds.) El Niño, su impacto en la fauna marina. *Bol. Extr. Inst. Mar Perú*, 153–158 (1985).

Samamé, M., & Fernández, F. Evaluación biológico pesquera del "falso volador" *Prionotus stephanophrys* Lockington, componente de la ictiofauna demersal del Perú. *Inf. Prog. Inst. Mar Perú* **126**, pp 28 (2000).

van der Heiden, A., Cotto, A., Rojas, P., Bearez, P., & Collette, B. *Prionotus stephanophrys*. The IUCN Red List of Threatened Species 2010: e.T183642A8150206. Available at: http://dx.doi.org/10.2305/IUCN.UK.2010-3.RLTS.T183642A8150206.en (2010).

1.12 Patagonian squid - *Doryteuthis gahi*

Sensitivity	
Abundance	
Fecundity – egg production (total fecundity)	Lays between 14 and 16 capsules with around 85 eggs each, which is a total of 1,300 eggs approximately (Cardoso *et al.* 2005).
Recruitment period – successful recruitment event that sustains the abundance of the fishery	Consistent recruitment events every 1–2 years but this may fail due to environmental variability and thus become variable.
Average age at maturity	Mature females were recorded at 13.6 ± 0.9 cm of mantle length (Cardoso *et al.* 1998). Because this species lives one year approximately, it is estimated that it reaches sexual maturity before 12 months of life (Villegas 2001).
Generalist vs. specialist – food and habitat	Paralarvae feed on zooplankton, whereas adults feed on fish, algae and polychaetes in rocky areas (Cardoso *et al.* 1998, 2005; Villegas 2001). This is a neritic species that frequents areas with sandy substrate or with shells to spawn (Cardoso *et al.* 2005), as well as rocky areas for feeding (Villegas 2001).

Biomass	Not available.
Distribution	
Capacity for larval dispersal or larval duration – hatching to settlement (benthic species), hatching to yolk sac re-adsorption (pelagic species).	Paralarvae may have considerable dispersal capacity with approximately 45 days subjected to the oceanic currents. However, spawning occurs at the bottom and it has been suggested that the dispersion may be limited (Ibáñez *et al.* 2016).
Capacity for adult/juvenile movement – lifetime range post-larval stage.	Not available.
Physiological tolerance – latitudinal coverage of adult species as a proxy of environmental tolerance.	Distributed from southern Peru (Puerto Pizarro, 3°30´S) to southern Chile (56°30´S), and is also found in the South Atlantic (Cardoso *et al.* 2005; Roper *et al.* 1984); approximately 56° of latitudinal coverage.<br>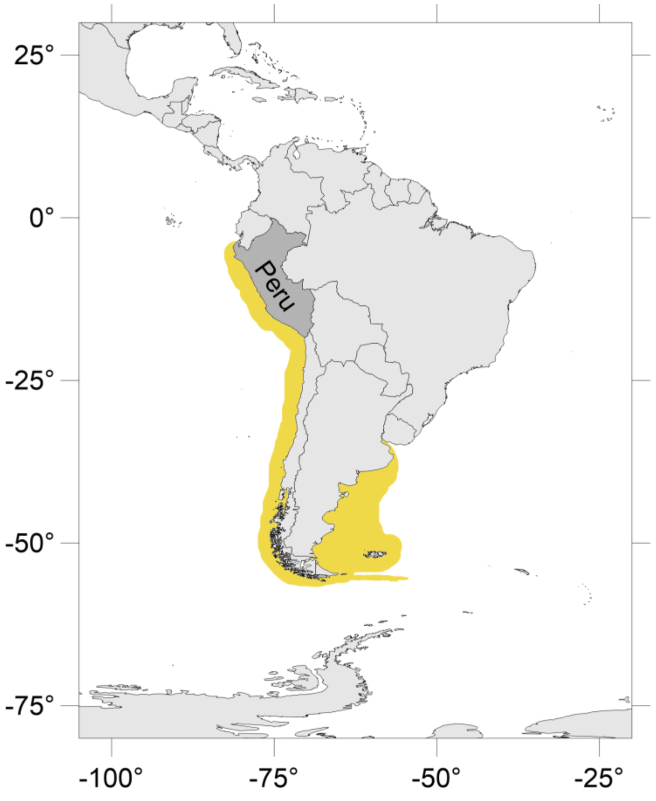
Spatial availability of unoccupied habitat for most critical life stage – ability to shift distributional range.	Approximately 41° of latitude may be available to the south of Peru.
Phenology	

Environmental variable as a phenological cue for spawning or breeding – cues include salinity, temperature, currents, and freshwater flows.	About 89% of embryos spawn at 18.5–19°C (Cardoso *et al.* 2005); with oceanic warming, embryonic development and spawning are likely to be affected.
Environmental variable as a phenological cue for settlement or metamorphosis	Although the relationship between temperature and growth is not clear, the highest growth rates were found in periods of high temperatures (Villegas 2001).
Temporal mismatches of life-cycle events – duration of spawning, breeding or moulting season.	The spawning seasons occur in spring, summer and autumn, but there are two main peaks in April and December, and a secondary peak in September/October (Villegas 2001). The presence of spermatophores in the buccal receptacle of immature females confirms that *D. gahi* is an intermittent spawner and therefore has several spawning periods (Cardoso *et al.* 1998). The duration of spawning is therefore thought to last over several months, i.e., > 4 months.
Migration (seasonal and spawning)	Adults occur between 400 and 600 m depth in winter, but in summer they migrate to surface waters to mate, lay eggs in the bottom at 8–70 m depth, and die (Arkhipkin *et al.* 2000; Villegas 2001; Laptikhovsky 2008).
Exposure	
Response to environmental variability	About 89% of embryos spawn at 18.5–19°C (Cardoso *et al.* 2005); with oceanic warming, embryonic development and spawning are likely to be affected. The increase in temperature during El Niño had a negative effect on catches of this species, while the decrease in temperature during La Niña had a positive effect (Villegas 2001). However, Ibáñez *et al.* (2016) indicate that thermal anomalies of El Niño did not have a significant effect on catches.<br><br>Although the relationship between temperature and growth is not clear, the highest growth rates were found in the period of high temperatures (Villegas 2001). High temperatures tend to accelerate growth rates and therefore the life span would decrease, which would be beneficial as the rate of population turnover would occur faster. However, also there would be negative effects, e.g., the size of the eggs would decrease, size at sexual maturity would be reached at smaller sizes, and therefore the population structure may change. Individuals would require more oxygen and food because of increased metabolic demand.

	Oceanic warming may also cause the mismatch of the reproductive cycle and of recruitment (Pecl & Jackson 2008). Ocean acidification and hypoxia may have negative effects on *D. gahi*, as observed in *D. gigas* (Rosa & Seibel 2008).

References

Arkhipkin, A.I., Laptikhovsky, V.V., & Middleton, D.A.J. Adaptations for cold water spawning in loliginid squid: *Loligo gahi* in Falkland waters. *J. Moll. Stud.* **66**, 551–564 (2000).

Cardoso, F., Baltazar, P., & Bautista, J. The early development of the Patagonian squid *Loligo gahi* D'Orbigny, 1835 in Peruvian waters (Cephalopoda: Loliginidae). *Rev. peru. biol.* **12**, 369–376 (2005).

Cardoso, F., Tarazona, J., & Paredes, C. Aspectos biológicos del calamar patagónico *Loligo gahi* (Cephalopoda: Loliginidae) en Huarmey, Perú. *Rev. peru. biol.* **5**, 9–14 (1998).

Roper, C.F.E., Sweeney, M.J., & Nauen, C.E. FAO Species catalogue Vol. 3. Cephalopods of the world. An Annotated and Illustrated Catalogue of Species of Interest to Fisheries. FAO Fisheries Synopsis No. 125. Available at: http://www.fao.org/fishery/species/2713/en (1984).

Ibáñez, C.M., Argüelles, J., Yamashiro, C., Sepúlveda, R.D., Pardo-Gandarillas, M.C., & Keyl, F. Population dynamics of the squids *Dosidicus gigas* (Oegopsida: Ommastrephidae) and *Doryteuthis gahi* (Myopsida: Loliginidae) in northern Peru. *Fish. Res.* **173**, 151–158 (2016)

Laptikhovsky, V. New data on spawning and bathymetric distribution of the Patagonian squid, *Loligo gahi*. *Mar. Biodivers. Rec.* **1**, e50 (2008).

Pecl, G.T., & Jackson, G.D. The potential impacts of climate change on inshore squid: biology, ecology and fisheries. *Rev. Fish Biol. Fisheries* **18**, 373–385 (2008).

Rosa, R., & Seibel, B.A. Synergistic effects of climate-related variables suggest future physiological impairment in a top oceanic predator. *Proc. Natl. Acad. Sci. USA* **105**, 20776–20780 (2008)

Villegas, P. Growth, life cycle and fishery biology of *Loligo gahi* (d'Orbigny, 1835) off the Peruvian coast. *Fish. Res.* **54**, 123–131 (2001).

1.13 Peruvian banded croaker - *Paralonchurus peruanus*

Sensitivity

Abundance	
Fecundity – egg production (total fecundity)	There is no information on fecundity but the partial fecundity of a species of the same genus, *P. brasiliensis*, in Brazilian coasts is 18,900 ± 9,500 oocytes (Costa *et al.* 2015).

Recruitment period – successful recruitment event that sustains the abundance of the fishery	Recruitment period is consistent every 1–2 years.
Average age at maturity	Sexual maturity is reached at 24 cm in length Froese & Pauly 2021); considering the size-age relationship it is estimated that maturity is reached at 3 years of age (Bringas 2012).
Generalist vs. specialist – food and habitat	It feeds on polychaetes, crustaceans, gastropods, teleosts and ophiuroids (Blaskovic' *et al.* 2008). Inhabits warm and temperate waters, sandy and muddy bottoms, and estuaries (Chirichigno & Cornejo 2001).
Biomass	According to the IUCN Red List this species is in the category LEAST CONCERN (LC) (Chao 2010), which means that it is abundant and widely distributed.
Distribution	
Capacity for larval dispersal or larval duration – hatching to settlement (benthic species), hatching to yolk sac re-adsorption (pelagic species).	Has a pelagic larval phase (EOL 2021). Unknown duration of the pelagic larval phase.
Capacity for adult/juvenile movement – lifetime range post-larval stage.	Not available.
Physiological tolerance – latitudinal coverage of adult species as a proxy of environmental tolerance.	It is distributed from Puerto Pizarro, Peru to Arica, Chile (Goicochea *et al.* 2012), although also there are records in Panama (Chao 2010); approximately 26° of latitudinal coverage.

	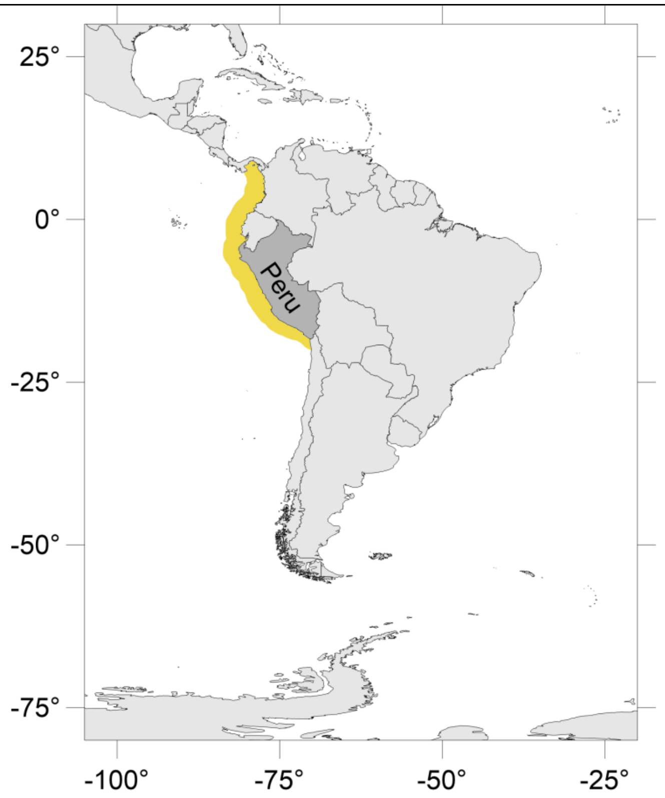
Spatial availability of unoccupied habitat for most critical life stage – ability to shift distributional range.	Approximately < 1° of latitude may be available to the south of Peru; nearly 13° of latitude may be available to the north of Peru.
Phenology	
Environmental variable as a phenological cue for spawning or breeding – cues include salinity, temperature, currents, and freshwater flows.	Positive thermal anomalies appear to result in the extension of spawning events (Llanos *et al.* 2009).
Environmental variable as a phenological cue for settlement or metamorphosis	Not available.
Temporal mismatches of life-cycle events – duration of spawning, breeding or moulting season.	Continuous reproductive activity throughout the year with a late summer-autumn spawning peak (Llanos *et al.* 2009; Bringas *et al.* 2014); duration of spawning is thus assumed to last > 4 months.
Migration (seasonal and spawning)	Not available.
Exposure	

Response to environmental variability	Demersal fish that inhabits the continental shelf and are associated with the Cronwell Subsurface Countercurrent, which oscillates intra and interannually. As a consequence, the habitat of these species also varies in size intra and interannually. In summer the habitat expands and in winter it is reduced, which results in lower and higher densities respectively. During El Niño events, the demersal system would behave as in summer conditions, depending on the intensity and duration of El Niño event. With El Niño, the diversity of the demersal system increases due to the incorporation of a greater number of species that move from north to south, from the coast to greater depths and from the pelagic system to the bottom (Velez *et al.* 1988; Espino 1990).<br><br>Changes in temperature, salinity and oxygen may have effects on growth (Bringas *et al.* 2014) and on the distribution of this species (Espino 1990). Positive thermal anomalies appear to result in the extension of spawning events (Llanos *et al.* 2009).

References

Blaskovic´, V., Espinoza, P., Fernández, C., Castillo, D., & Navarro, I. Espectro alimentario de las principales especies demersales de importancia comercial y sus relaciones intraespecíficas en el 2007. Informe técnico anual. Dirección de Investigaciones en peces demersales y litorales. *Inst. Mar Perú*. pp 38 (2008).

Bringas, A.F. Biología y pesquería de *Paralonchurus peruanus* "suco" desembarcado en la región La Libertad de Mayo 2011 – Abril 2012. Dissertation, Universidad Nacional de Trujillo. Trujillo, Perú. pp 50 (2012).

Bringas, A., Culquichicón, Z., & Atoche, D. Biología y pesquería de *Paralonchurus peruanus* "Suco" en la región La Libertad, Mayo 2011-Abril 2012. *Sciendo* **17**, 120–135 (2014).

Chao, L. *Paralonchurus peruanus*. The IUCN Red List of Threatened Species 2010: e.T183581A8139098. Available at: http://dx.doi.org/10.2305/IUCN.UK.2010-3.RLTS.T183581A8139098.en (2010).

Chirichigno, N., & Cornejo, R.M. Catálogo comentado de los peces marinos del Perú. *Inst. Mar Perú*. Callao, Perú (2001).

Costa, E.F.S., Días, J.F., & Murua, H. Reproductive strategy and fecundity of the keystone species *Paralonchurus brasiliensis* (Teleostei, Sciaenidae): an image processing techniques application. *Environ. Biol. Fish.* **98**, 2093–2108 (2015).

EOL – Encyclopedia of Life. Available at: https://eol.org/pages/46579206 (2021).

Espino, M. El Niño: Su impacto sobre los peces demersales del Perú. *Bol. Inst. Mar Perú* **14**, pp 28 (1990).

Froese, R., & Pauly, D. (eds.) FishBase. World Wide Web electronic publication. Available at: http://www.fishbase.org/summary/424 (2021).

Goicochea, C., Arrieta, S.B., Moquillaza, P., & Mostacero, J. Edad y crecimiento de *Paralonchurus peruanus* (Steindachner) en aguas de la costa central del Perú, 1999. *Inf. Inst. Mar Perú* **39**, 26–33 (2012).

Llanos, J. *et al.* Investigaciones de IMARPE-Sede Lambayeque durante 2009. pp 89 (2009).

Velez, J., Espino, M., & Zeballos, J. Variación de la ictiofauna demersal frente al Perú entre 1981 y 1987. *Memorias del 2do Congreso Latinoeamericano sobre Ciencias del Mar* (COLACMAR), 17–21 Agosto de 1987, Lima, Perú. Tomo I: 203–212 (1988).

1.14 Peruvian hake - *Merluccius gayi peruanus*

Sensitivity	
Abundance	
Fecundity – egg production (total fecundity)	The partial fecundity *M. gayi peruanus* is 50,856 oocytes (Perea de la Matta *et al.* 2005).
Recruitment period – successful recruitment event that sustains the abundance of the fishery	Recruitment period is usually consistent every 1–2 years.
Average age at maturity	The size and age at maturity are unknown for this species; however *M. gayi gayi* reaches the age of 50% maturity at 3.5 years in Chile (http://www.subpesca.cl/institucional/602/articles-9175_documento.pdf).
Generalist vs. specialist – food and habitat	It feeds mainly on crustaceans, fish and cephalopods (Blaskovic' *et al.* 2008). This is a batidemersal species that inhabits from the coastal zone to a depth range of 50–500 m depth on sandstone or non-muddy clay bottoms (Chirichigno & Cornejo 2001; Iwamoto *et al.* 2008).
Biomass	In 2010, the spawning biomass was close to 100,000 t, while in autumn 2012 a total biomass of 189,772 ± 56,327 t was estimated (IMARPE 2012, unpublished). According to the IUCN Red List this species is in the category UNCERTAIN (Iwamoto *et al.* 2010), i.e., there are not enough data.
Distribution	

Capacity for larval dispersal or larval duration – hatching to settlement (benthic species), hatching to yolk sac re-adsorption (pelagic species).	The yolk sac is likely to be rapidly absorbed, as per *M. australis* at 9 days (Bustos & Landaeta 2005).
Capacity for adult/juvenile movement – lifetime range post-larval stage.	Performs migrations that are determined by seasonal (summer-autumn) and interannual changes associated with El Niño events in the Cromwell Current (Wosnitza-Mendo *et al.* 2009).
Physiological tolerance – latitudinal coverage of adult species as a proxy of environmental tolerance.	Distributed from the border between Ecuador and Peru to Huarmey, and occasionally it occurs further south to Ilo (Chirichigno & Cornejo 2001); approximately 35° of latitudinal coverage. 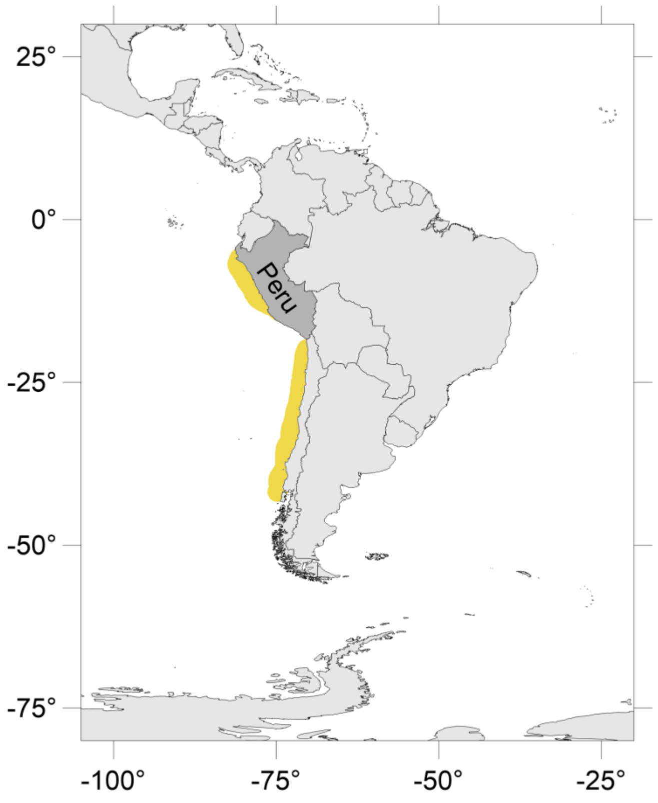
Spatial availability of unoccupied habitat for most critical life stage – ability to shift distributional range.	Approximately 22° of latitude may be available to the south of Peru.
Phenology	
Environmental variable as a phenological cue for spawning or breeding – cues include salinity,	Not available.

temperature, currents, and freshwater flows.	
Environmental variable as a phenological cue for settlement or metamorphosis	Not available.
Temporal mismatches of life-cycle events – duration of spawning, breeding or moulting season.	The main spawning season is from August to March, with a peak in spring (Iwamoto *et al.* 2010); duration of spawning is thus assumed to last > 4 months.
Migration (seasonal and spawning)	Performs migrations that are determined by seasonal (summer-autumn) and interannual changes associated with El Niño events in the Cromwell Current (Wosnitza-Mendo *et al.* 2009).
Exposure	
Response to environmental variability	With the intrusion of oceanic waters, the Peruvian hake tends to become pelagic and coastal mainly in the area of Chimbote-Huarmey. If the species is not able to return to its spawning area then an increase in population size occurs (Samamé *et al.* 1985). During El Niño events, demersal fishes tend to deepen (Espino 1990). The Peruvian hake usually occurs between 0.75 mL/L and 1.75 mL/L of dissolved oxygen, but during El Niño events it can occur between 1 mL/L and 2 mL/L of dissolved oxygen (Espino 1990).

References

Blaskovic´, V., Espinoza, P., Fernández, C., Castillo, D., & Navarro, I. Espectro alimentario de las principales especies demersales de importancia comercial y sus relaciones intraespecíficas en el 2007. Informe técnico anual. Dirección de Investigaciones en peces demersales y litorales. *Inst. Mar Perú.* pp 38 (2008).

Bustos, C.A., & Landaeta, M.F. Desarrollo de huevos y larvas tempranas de la merluza del sur, *Merluccius australis*, cultivados bajo condiciones de laboratorio. *Gayana* **69**, 402–408 (2005).

Chirichigno, N., & Cornejo, R.M. Catálogo comentado de los peces marinos del Perú. *Inst. Mar Perú*. Callao, Perú (2001).

Espino, M. El Niño: Su impacto sobre los peces demersales del Perú. *Bol. Inst. Mar Perú* **14**, pp 28 (1990).

IMARPE. Ciclo de charlas científicas. (2012).

Iwamoto, T., Eschmeyer, W., Alvarado, J., & Bussing, W. *Merluccius gayi*. The IUCN Red List of Threatened Species 2010: e.T183527A8128809. Available at: http://dx.doi.org/10.2305/IUCN.UK.2010-3.RLTS.T183527A8128809.en (2010).

Perea de la Matta, A., Roque-García, C., & Buitrón-Díaz, B. Actividad reproductiva de merluza, *Merluccius gayi peruanus* en verano 2005. Crucero BIC Olaya 0501–02. *Inf. Inst. Mar Perú* **39**, 275–279 (2005).

Samamé, M., Castillo, J., & Mendieta, A. Situación de las pesquerías demersales y los cambios durante El Niño. In: Arntz, W., Landa, A., & Tarazona, J. (eds.) El Niño, su impacto en la fauna marina. *Bol. Extr. Inst. Mar Perú*, 153–158 (1985).

Wosnitza-Mendo, C., Ballón, M., Benites, C., & Guevara-Carrasco, R. Cambios en el área de distribución de la merluza peruana: efecto de la pesquería y El Niño. *Bol. Inst. Mar Perú* **24**, 29–38 (2009).

1.15 Peruvian rock seabass - *Paralabrax humeralis*

Sensitivity	
Abundance	
Fecundity – egg production (total fecundity)	There is no information on the fecundity of this species, however the fecundity of *P. maculatofasciatus* is 284,000 eggs/kg of female (Avilés 2005).
Recruitment period – successful recruitment event that sustains the abundance of the fishery	Recruitment period is consistent every 1–2 years.
Average age at maturity	The size at 50% sexual maturity in females is 24.5 cm of total length (Miñano & Castillo 1971), a size that is reached between 2 years and 3 years of age (Goicochea *et al.* 2012).
Generalist vs. specialist – food and habitat	Adults feed on crustaceans, fish, cephalopods, polychaetes, euphausiids (Blaskovic' *et al.* 2008). Generally inhabits sandy and rocky coastal areas with algae down to 180 m depth (Miñano & Castillo 1971; Smith-Vaniz *et al.* 2010); juveniles are located near the coast (Chirichigno & Cornejo 2001).
Biomass	The estimated biomass between 1981 and 1988 was 30,000 t, which fluctuates depending on environmental conditions, e.g., the biomass of *P. humeralis* increases as the density of hake decreases during El Niño (Espino 1990). According to the IUCN Red List this species is in the category UNCERTAIN (Smith-Vaniz *et al.* 2010), i.e., there are insufficient data.
Distribution	

Capacity for larval dispersal or larval duration – hatching to settlement (benthic species), hatching to yolk sac re-adsorption (pelagic species).	Under controlled conditions, larvae of species of the family Serranidae take 2–3 days to consume their yolk sack (Tucker 1998).
Capacity for adult/juvenile movement – lifetime range post-larval stage.	Not available.
Physiological tolerance – latitudinal coverage of adult species as a proxy of environmental tolerance.	Distributed from Ecuador to the southern part of Chile and the islands Juan Fernández and Galápagos (Goicochea *et al.* 2012); approximately 37° of latitudinal coverage. 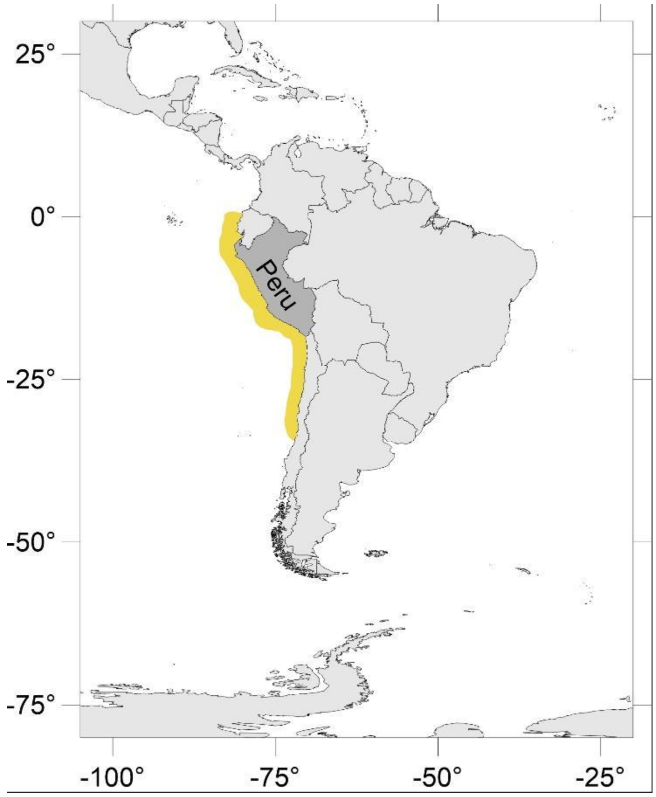
Spatial availability of unoccupied habitat for most critical life stage – ability to shift distributional range.	Approximately 19° of latitude may be available to the south of Peru.
Phenology	
Environmental variable as a phenological cue for spawning or breeding – cues include salinity, temperature, currents, and freshwater flows.	Not available.

Environmental variable as a phenological cue for settlement or metamorphosis	Not available.
Temporal mismatches of life-cycle events – duration of spawning, breeding or moulting season.	The Peruvian rock seabass reproduces from November to August with the main spawning peak in March on the north coast of Peru, i.e., in Chimbote (Mejía *et al.* 1970; Miñano & Castillo 1971); duration of spawning is thus assumed to last > 4 months.
Migration (seasonal and spawning)	Not available.
Exposure	
Response to environmental variability	The Peruvian rock seabass usually occurs at 14–16°C and 0.75–1.75 mL/L of dissolved oxygen but during El Niño it occurs between 17–20°C and 2.25–3.25 mL/L of dissolved oxygen (Espino 1990). In general, demersal fishes tend to deepen during El Niño events (Espino 1990). With El Niño 1983 the larger fish migrated south of the main fishing area, and small fishes were accessible to the fishery (Samamé *et al.* 1985). The abundance of the Peruvian rock seabass decreased during El Niño (Espino 1990). In contrast, its immediate availability increased during La Niña and CPUE decreased between six months and two years after that event (Adams & Flores 2016). Demersal fish inhabit the continental shelf and are associated with the Cronwell Subsurface Countercurrent, which oscillates intra and interannually. As a consequence, the habitat of these species also varies in size intra and interannually. In summer the habitat expands and in winter it is reduced, which results in lower and higher densities respectively. During El Niño events, the demersal system would behave as in summer conditions, depending on the intensity and duration of El Niño event. With El Niño, the diversity of the demersal system increases due to the incorporation of a greater number of species that move from north to south, from the coast to greater depths and from the pelagic system to the bottom (Velez *et al.* 1988; Espino 1990). Biomass fluctuates depending on environmental conditions, e.g., the biomass of *P. humeralis* increases as the density of hake decreases during El Niño (Espino 1990).
References	

Adams, G.D., & Flores, D. Influencia de El Niño Oscilación del Sur en la disponibilidad y abundancia de recursos hidrobiológicos de la pesca artesanal en Ica, Perú. *Rev. Biol. Mar. Oceanogr.* **51**, 265–272 (2016).

Avilés, M.A. Calidad de huevos y larvas según el manejo de los reproductores de la cabrilla (*Paralabrax maculatofasciatus*, Pisces: Serranidae). Dissertation, Universitat de Barcelona (2005).

Blaskovic´, V., Espinoza, P., Fernández, C., Castillo, D., & Navarro, I. Espectro alimentario de las principales especies demersales de importancia comercial y sus relaciones intraespecíficas en el 2007. Informe técnico anual. Dirección de Investigaciones en peces demersales y litorales. *Inst. Mar Perú.* pp 38 (2008).

Chirichigno, N., & Cornejo, R.M. Catálogo comentado de los peces marinos del Perú. *Inst. Mar Perú*. Callao, Perú (2001).

Espino, M. El Niño: Su impacto sobre los peces demersales del Perú. *Bol. Inst. Mar Perú* **14**, pp 28 (1990).

Goicochea, C., Moquillaza, P., & Mostacero, J. Edad y crecimiento de *Paralabrax humeralis* (Valenciennes) en el mar del Callao, 1996. *Inf. Inst. Mar Perú* **39**, 23–25 (2012)

Mejía, J., Samamé, M., & Pastor, A. Información básica de los principales peces de consumo. *Instituto del Mar, Serie de Informes Especiales IM–62*. pp 29 (1970).

Miñano, J.B., & Castillo, J. Investigación biológica preliminar de la "cabrilla" *Paralabrax humeralis* (Valenciennes) en Chimbote. *Instituto del Mar. Serie de Informes Especiales IM–83*. pp 8 (1971).

Samamé, M., Castillo, J., & Mendieta, A. Situación de las pesquerías demersales y los cambios durante El Niño. In: Arntz, W., Landa, A., & Tarazona, J. (eds.) El Niño, su impacto en la fauna marina. *Bol. Extr. Inst. Mar Perú*, 153–158 (1985).

Smith-Vaniz, B., *et al*. *Paralabrax humeralis*. The IUCN Red List of Threatened Species 2010: e.T183746A8169144. Available at: http://dx.doi.org/10.2305/IUCN.UK.2010-3.RLTS.T183746A8169144.en (2010).

Tucker, J. Marine Fish Culture. Kluwer Academic Publishers, USA (1998).

Velez, J., Espino, M., & Zeballos, J. Variación de la ictiofauna demersal frente al Perú entre 1981 y 1987. *Memorias del 2do Congreso Latinoeamericano sobre Ciencias del Mar* (COLACMAR), 17–21 Agosto de 1987, Lima, Perú. Vol I: 203–212 (1988).

1.16 Peruvian sea catfish - *Galeichthys peruvianus*

Sensitivity	
Abundance	
Fecundity – egg production (total fecundity)	Its fecundity is 27 eggs during the spawning season in summer (Castañeda *et al.* 2007; Bearez *et al.* 2010).

Recruitment period – successful recruitment event that sustains the abundance of the fishery	Likely consistent recruitment events every 1–2 years.
Average age at maturity	Reaches the length of first maturity at 21.7 cm (Llanos *et al.* 2009), approximately at one year of life.
Generalist vs. specialist – food and habitat	Catfishes prey upon fish, crustaceans, polychaetes, molluscs and algae (Llanos *et al.* 2009). The anchovy is a common prey mainly in summer and autumn (Castañeda *et al.* 2007). Inhabits shallow waters with soft bottoms, generally within 50 nautical miles from the coast (Castañeda *et al.* 2007).
Biomass	The total biomass of *G. peruvianus* in summer 2006 in northern Peru off Pimentel-Chicama and Pascamayo was 236,632 t (Castillo *et al.* 2009). According to the International Union for the Conservation of Nature (IUCN) Red List this species is in the category LEAST CONCERN (LC) (Bearez *et al.* 2010), which means that it is abundant and widely distributed.
Distribution	
Capacity for larval dispersal or larval duration – hatching to settlement (benthic species), hatching to yolk sac re-adsorption (pelagic species).	Not available.
Capacity for adult/juvenile movement – lifetime range post-larval stage.	Performs seasonal migrations; during winter the main densities occur in the coastal zone at 7–9°S and during spring it moves towards 8–10°S (Castañeda *et al.* 2007; Bearez *et al.* 2010), which is approximately a 100–300 km migration.
Physiological tolerance – latitudinal coverage of adult species as a proxy of environmental tolerance.	Distributed from northern Peru to northern Chile (Bearez *et al.* 2010); approximately 15° of latitudinal coverage.

	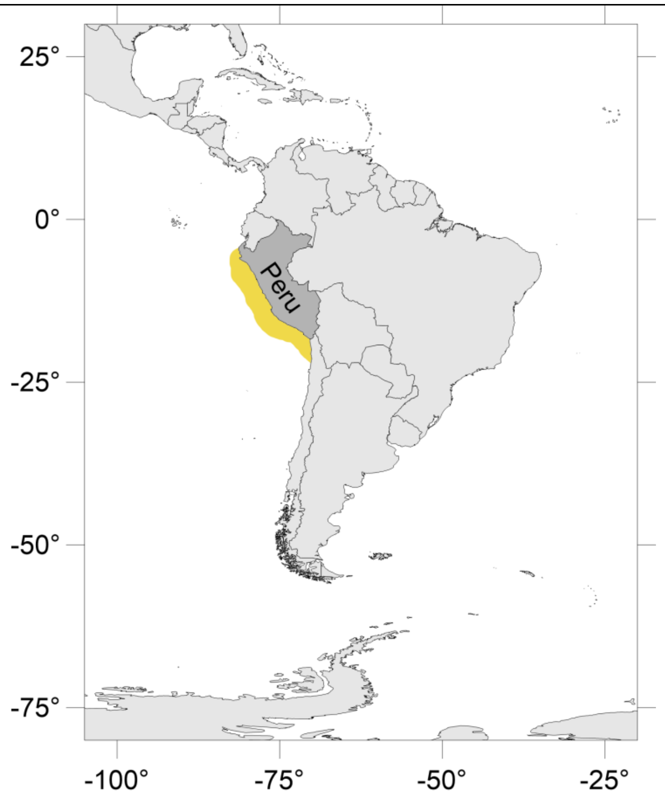
Spatial availability of unoccupied habitat for most critical life stage – ability to shift distributional range.	Approximately 2° of latitude may be available to the south of Peru.
Phenology	
Environmental variable as a phenological cue for spawning or breeding – cues include salinity, temperature, currents, and freshwater flows.	During 2009, the reproductive pattern of this species was altered due to negative anomalies occurring during the first three months of the year, so spawning was delayed until May and secondary peaks were more intense due to high temperatures in the second half of the year (Llanos *et al.* 2009). A species of the same genus, *G. caerulescens*, requires low salinities to spawn (Yáñez-Arancibia *et al.* 1976); therefore, changes in the frequency and intensity of rainfall could have consequences on the spawning events near the coast.
Environmental variable as a phenological cue for settlement or metamorphosis	Not available.
Temporal mismatches of life-cycle events – duration of spawning, breeding or moulting season.	Has a major spawning peak that occurs in late summer (Llanos *et al.* 2009); duration of spawning is thus assumed to last 2–4 months.

Migration (seasonal and spawning)	Performs seasonal migrations; during winter the main densities occur in the coastal zone at 7–9°S and during spring it moves towards 8–10°S (Castañeda *et al.* 2007; Bearez *et al.* 2010), which is approximately a 100–300 km migration. After spawning, males bring the fertilized eggs to shallow areas of the coast while the females return to their usual areas of distribution. A species of the same genus, *G. caerulescens*, performs migrations into lagoons during the breeding season (Yáñez-Arancibia *et al.* 1976). Juveniles become pelagic and eventually move to greater depths (Castañeda *et al.* 2007; Bearez *et al.* 2010).
Exposure	
Response to environmental variability	Demersal fish inhabit the continental shelf and are associated with the Cronwell Subsurface Countercurrent, which oscillates intra and interannually. As a consequence, the habitat of these species also varies in size intra and interannually. In summer the habitat expands and in winter it is reduced, which results in lower and higher densities respectively. During El Niño events, the demersal system would behave as in summer conditions, depending on the intensity and duration of El Niño event. With El Niño, the diversity of the demersal system increases due to the incorporation of a larger number of species that move from north to south, from the coast to greater depths and from the pelagic system to the bottom (Velez *et al.* 1988). In fact, the dispersal of the *G. peruvianus* population along the coast during warm events, such as summer or El Niño has been recorded (Bearez *et al.* 2010).<br><br>During 2009, the reproductive pattern of this species was altered due to negative anomalies occurring during the first three months of the year, so spawning was delayed until May and secondary peaks were more intense due to the high temperatures recorded in the second half of the year (Llanos *et al.* 2009). A species of the same genus, *G. caerulescens*, requires low salinities to spawn (Yáñez-Arancibia *et al.* 1976); therefore, changes in the frequency and intensity of rainfall could have consequences on the spawning events near the coast.
References	

Bearez, P., Acero, A., & Betancur, R. *Galeichthys peruvianus*. The IUCN Red List of Threatened Species 2010: e.T183404A8107700. Available at: http://dx.doi.org/10.2305/IUCN.UK.2010-3.RLTS.T183404A8107700.en (2010).

Castañeda, J., Carbajal, W., Galán, J., & Gutiérrez, M. Bioecología del bagre *Galeichthys peruvianus* en el mar del Perú. Periodo 1998–2004. *Inf. Inst. Mar Perú* **34**, 295–307 (2007).

Castillo, R., Gutiérrez, M., Peraltilla, S., & Escudero, L. Distribución y biomasa de los principales recursos pelágicos del mar peruano. Verano 2006. *Inf. Inst. Mar Perú* **36**, 121–130 (2009).

Llanos, J. *et al.* Investigaciones de IMARPE-Sede Lambayeque durante 2009. pp 89 (2009).

Velez, J., Espino, M., & Zeballos, J. Variación de la ictiofauna demersal frente al Perú entre 1981 y 1987. *Memorias del 2do Congreso Latinoeamericano sobre Ciencias del Mar* (COLACMAR), 17–21 Agosto de 1987, Lima, Perú. Tomo I: 203–212 (1988).

Yáñez-Arancibia, A., Curiel-Gómez, J., & De Yáñez, V.L. Prospección biológica y ecológica del bagre marino *Galeichthys caerulescens* (Gunther) en el sistema lagunar costero de Guerrero, México (Pisces: Ariidae). *Anales del Centro de Ciencias del Mar y Limnología.* pp 67 (1976).

1.17 Peruvian weakfish - *Cynoscion analis*

Sensitivity	
Abundance	
Fecundity – egg production (total fecundity)	The fecundity of *C. analis* is unknown; however, *C. leiarchus* off Brazilian coasts has a partial fecundity of 100,000–866,000 oocytes (Carmo-Silva *et al.* 2016).
Recruitment period – successful recruitment event that sustains the abundance of the fishery	Two pulses of recruitment to the fishery have been identified, the main one between June and July and the pulse of lower intensity between January and February (Farroñay *et al.* 2010). Hence, consistent recruitment events every 1–2 years.
Average age at maturity	The length at first maturity is about 24 cm and the length at 50% of sexual maturity is at 27 cm (Mejía *et al.* 1970; Llanos *et al.* 2009).
Generalist vs. specialist – food and habitat	The Peruvian weakfish feeds mainly on crustaceans, fish and cephalopods (Blaskovic' *et al.* 2008). This is a species of warm and temperate waters that inhabits sandy, muddy and rocky bottoms (Mejía *et al.* 1970), until approximately 50 m depth (Chao & Espinosa 2010).

Biomass	In 1983, only in the area of Paita, Peru, the biomass of this species was estimated at approximately 23,000 tons (Mendo *et al.* 1988). According to the IUCN Red List this species is in the category LEAST CONCERN (LC) (Chao & Espinosa 2010), which means that it is abundant and widely distributed.
Distribution	
Capacity for larval dispersal or larval duration – hatching to settlement (benthic species), hatching to yolk sac re-adsorption (pelagic species).	The larvae may consume the yolk sac in only a few days, as observed in larvae of *C. nebulosus*, whose yolk sac is absorbed within 48 h post-hatching (Ibarra-Castro *et al.* 2015).
Capacity for adult/juvenile movement – lifetime range post-larval stage.	Not available.
Physiological tolerance – latitudinal coverage of adult species as a proxy of environmental tolerance.	Endemic to the Eastern Pacific and is distributed from Santa Elena, Ecuador to Coquimbo, Chile (Froese & Pauly 2021). A reduced range of distribution also has been described and comprises from Colombia to the north of Peru (Chao & Espinosa 2010); approximately 28° of latitudinal coverage considering both ranges of distribution. 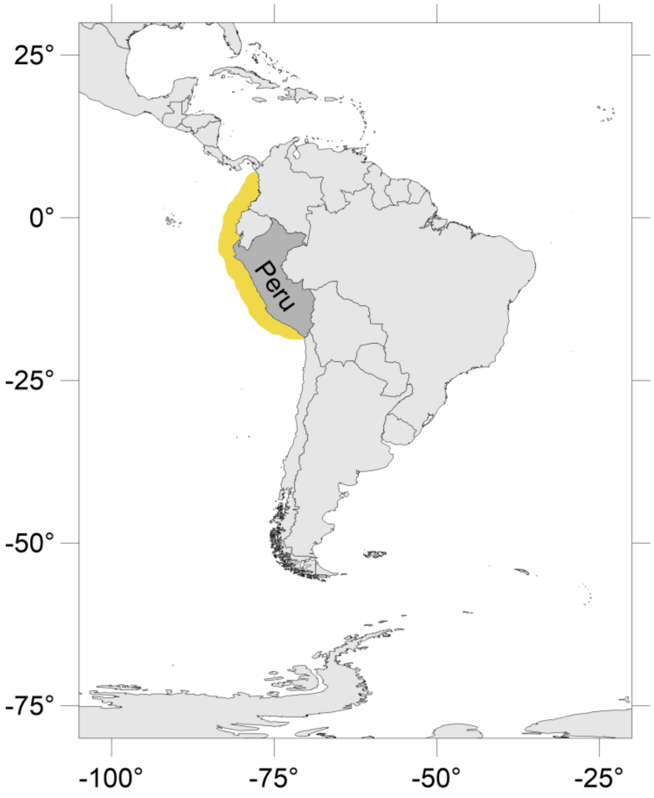

Spatial availability of unoccupied habitat for most critical life stage – ability to shift distributional range.	Approximately 11° of latitude may be available to the south of Peru.
Phenology	
Environmental variable as a phenological cue for spawning or breeding – cues include salinity, temperature, currents, and freshwater flows.	Salinity may have an effect on the spawnings of *C. analis*; for instance, although *C. nubelosus* tolerates a wide range of salinity, spawning is only carried out between salinities of 20 to 40 (Ibarra-Castro *et al.* 2015).
Environmental variable as a phenological cue for settlement or metamorphosis	Not available.
Temporal mismatches of life-cycle events – duration of spawning, breeding or moulting season.	The main spawning peak is in summer, and secondary peaks occur throughout the year (Llanos *et al.* 2009); duration of spawning is thus assumed to last > 4 months.
Migration (seasonal and spawning)	Not available.
Exposure	
Response to environmental variability	In general, demersal fishes tend to deepen during El Niño (Espino 1990). During normal years the Peruvian weakfish occurs in temperate waters but during El Niño it occurs at higher temperatures (Espino 1990). Demersal fish inhabit the continental shelf and are associated with the Cronwell Subsurface Countercurrent, which oscillates intra and interannually. As a consequence, the habitat of these species also varies in size intra and interannually. In summer the habitat expands and in winter it is reduced, which results in lower and higher densities respectively. During El Niño events, the demersal system would behave as in summer conditions, depending on the intensity and duration of El Niño event. With El Niño, the diversity of the demersal system increases due to the incorporation of a greater number of species that move from north to south, from the coast to greater depths and from the pelagic system to the bottom (Velez *et al.* 1988; Espino 1990). Changes in distribution due to El Niño events also have resulted in changes in the size structure (Samamé *et al.* 1985). With oceanic warming, the main spawning peak is delayed and the secondary peaks become more intense (Llanos *et al.* 2009).

	Salinity may have an effect on the spawnings of *C. analis*; for instance, although *C. nubelosus* tolerates a wide range of salinity, spawning is only carried out between salinities of 20 to 40 (Ibarra-Castro *et al.* 2015). The Peruvian weakfish generally occurs between 1 mL/L and 1.75 mL/L of dissolved oxygen, whereas during El Niño it occurs between 2 mL/L and 2.25 mL/L of dissolved oxygen (Espino 1990).

References

Blaskovic´, V., Espinoza, P., Fernández, C., Castillo, D., Navarro, I. Espectro alimentario de las principales especies demersales de importancia comercial y sus relaciones intraespecíficas en el 2007. Informe técnico anual. Dirección de Investigaciones en peces demersales y litorales. *Inst. Mar Perú.* pp 38 (2008).

Carmo-Silva, J.P., da Costa, M.R., Gomes, I.D., & Araújo, F.G. Gonadal development and fecundity of the smooth weakfish *Cynoscion leiarchus* (Teleostei: Perciformes: Sciaenidae) in a tropical Brazilian bay. *Zoologia* **33**, e20160032 (2016).

Chao, L., & Espinosa, H. *Cynoscion analis*. The IUCN Red List of Threatened Species 2010: e.T183954A8205810. Available at: http://dx.doi.org/10.2305/IUCN.UK.2010-3.RLTS.T183954A8205810.en (2010).

Espino, M. El Niño: Su impacto sobre los peces demersales del Perú. *Bol. Inst. Mar Perú* **14**, pp 28 (1990).

Farroñay, L., Oliva, J., & Castañeda, J. Dinámica poblacional de *Cynoscion analis* “cachema” en el litoral de Lambayeque, 2006. *Cienc. tecnol. humanid.* **1**, 81–93 (2010).

Froese, R., & Pauly, D. (eds.) FishBase. World Wide Web electronic publication. Available at: http://www.fishbase.org/summary/Cynoscion-analis.html (2021).

Ibarra-Castro, L., Gutiérrez-Sigeros, I., Alvarez-Lajonchere, L., Durruty-Lagunes, C.V., & Sánchez-Zamora, A. Desempeño reproductivo y primeros estadios de vida en corvina pinta *Cynoscion nebulosus* en cautiverio. *Rev. Biol. Mar. Oceanogr.* **50**, 439–451 (2015).

Llanos, J. *et al.* Investigaciones de IMARPE-Sede Lambayeque durante 2009. pp 89 (2009).

Mejía, J., Samamé, M., & Pastor, A. Información básica de los principales peces de consumo. *Instituto del Mar, Serie de Informes Especiales IM–62*. pp 29 (1970)

Mendo, J., Samamé, M., Wosnitza-Mendo, C., Mendieta, A., & Castillo, J. Análisis biológico-pesquero y poblacional de la cachema (*Cynoscion analis*) del área de Paita, Perú. *Bol. Inst. Mar Perú* **12**, 23–57 (1988).

Samamé, M., Castillo, J., & Mendieta, A. Situación de las pesquerías demersales y los cambios durante El Niño. In: Arntz, W., Landa, A., & Tarazona, J. (eds.) El Niño, su impacto en la fauna marina. *Bol. Extr. Inst. Mar Perú*, 153–158 (1985).

Velez, J., Espino, M., Zeballos, J. Variación de la ictiofauna demersal frente al Perú entre 1981 y 1987. *Memorias del 2do Congreso Latinoeamericano sobre Ciencias del Mar* (COLACMAR), 17–21 Agosto de 1987, Lima, Perú. Vol I: 203–212 (1988).

Pelagic

**1.18 Blue shark - *Prionace glauca***	
Sensitivity	
Abundance	
Fecundity – egg production (total fecundity)	In South African waters this species produces between 43 and 55 embryos (Jolly *et al.* 2013)
Recruitment period – successful recruitment event that sustains the abundance of the fishery	Likely consistent recruitment events every 1–2 years, although these may be variable.
Average age at maturity	Size of sexual maturity is reached at 170–190 cm (Gonzalez-Pestana *et al.* 2016), approximately at 6 years of age (Jolly *et al.* 2013).
Generalist vs. specialist – food and habitat	Feeds on teleosts, squids, and other invertebrates, it also has scavenger habits and occasionally feeds on birds. Many of their prey are pelagic, although they also feed on fish and bottom invertebrates (Compagno 1984). This is an epipelagic and neritic oceanic species that can be found from the surface to 350 m depth (Campana *et al.* 2011).
Biomass	According to the IUCN Red List, this species is in the category NEAR THREATENED (NT) (Stevens 2009), which means that it is not currently vulnerable or endangered but is likely to be in the near future.
Distribution	
Capacity for larval dispersal or larval duration – hatching to settlement (benthic species), hatching to yolk sac re-adsorption (pelagic species).	Not available.
Capacity for adult/juvenile movement – lifetime range post-larval stage.	In the Northwest Atlantic it is able to carry out movements up to 2,500 km in 210 days (Campana *et al.* 2011).

Physiological tolerance – latitudinal coverage of adult species as a proxy of environmental tolerance.	Occurs in all oceans; in the eastern Pacific this species occurs from the Gulf of Alaska to the southern region of Chile (Compagno 1984); approximately 116° of latitudinal coverage. 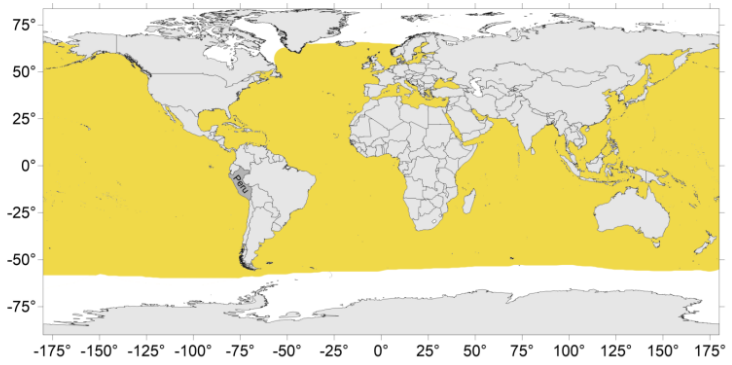
Spatial availability of unoccupied habitat for most critical life stage – ability to shift distributional range.	Approximately 41° of latitude may be available to the south of Peru; approximately 62° of latitude may be available to the north of Peru.
Phenology	
Environmental variable as a phenological cue for spawning or breeding – cues include salinity, temperature, currents, and freshwater flows.	Not available.
Environmental variable as a phenological cue for settlement or metamorphosis	Not available.
Temporal mismatches of life-cycle events – duration of spawning, breeding or moulting season.	In the eastern Pacific, pregnant females are more frequent in February, March, May, July and August (Zhu *et al.* 2011). The gestation period lasts 9 to 12 months (Pratt 1979).
Migration (seasonal and spawning)	In the North Atlantic, the blue shark performs seasonal migrations influenced by water temperature, coupled with the availability of prey and its reproductive status (Kohler *et al.* 2002).
Exposure	
Response to environmental variability	This species prefers relatively temperate waters (7–16°C) but can tolerate temperatures higher than 21°C (Compagno 1984). In the North Atlantic, the blue shark performs seasonal migrations influenced by water temperature, coupled with the availability of prey and its reproductive status (Kohler *et al.* 2002). The landing of this species has

	decreased during El Niño events. As a consequence, with the oceanic warming the species is expected to change in distribution. Therefore, its abundance is likely to change in different areas and affect positively or negatively its capture in certain areas.

References

Campana, S.E., *et al.* Migration pathways, behavioural thermoregulation and overwintering grounds of blue sharks in the Northwest Atlantic. *PLoS ONE* **6**, e16854 (2011).

Compagno, L.J.V. FAO species catalogue Vol. 4. Sharks of the world. An Annotated and Illustrated Catalogue of Shark Species Known to Date Part 2 - Carcharhiniformes. FAO Fish. Synop. 125. Available at: http://www.fao.org/fishery/species/2018/en (1984).

Gonzalez-Pestana, A., Kouri, C., & Velez-Zuazo, X. Shark fisheries in the Southeast Pacific: A 61-year analysis from Peru. *F1000Res.* **3**, 164 (2016).

Jolly, K.A., da Silva, C., & Attwood, C.G. Age, growth and reproductive biology of the blue shark *Prionace glauca* in South African waters. *Afr. J. Mar. Sci.* **35**, 99–109 (2013).

Kohler, N.E., Turner, P.A., Hoey, J.J., Natanson, L.J., & Briggs, R. Tag and recapture data for three pelagic shark species; blue shark (*Prionace glauca*), shortfin mako (*Isurus xyrinchus*), and porbeagle (*Lamna nasus*) in the North Atlantic Ocean. *ICCAT Collective Volume of Scientific Papers* **54**, 1231–1260 (2002).

Pratt, H.L. Reproduction in the blue shark, *Prionace glauca*. *Fish. Bull.* **77**, 445–470 (1979).

Stevens, J. *Prionace glauca*. The IUCN Red List of Threatened Species 2009: e.T39381A10222811. Available at: http://dx.doi.org/10.2305/IUCN.UK.2009-2.RLTS.T39381A10222811.en (2009).

Zhu, J., Dai, X., Xu, L., Chen, X., & Chen, Y. Reproductive biology of female blue shark *Prionace glauca* in the southeastern Pacific Ocean. *Environ. Biol. Fish.* **91**, 95–102 (2011).

1.19 Chilean jack mackerel - *Trachurus murphyi*

Sensitivity

Abundance	
Fecundity – egg production (total fecundity)	Approximately 78,789 eggs per spawning batch; females larger than 35 cm total length can produce 162,590 eggs per spawning batch (Dioses *et al.* 1988).
Recruitment period – successful recruitment event that sustains the abundance of the fishery	It is assumed that the recruitment is annual (PRODUCE-IMARPE 2009) with 2–3 years old individuals being recruited (Vásquez *et al.* 2013).

Average age at maturity	This species reaches sexual maturity at 3 years (Marzloff *et al.* 2009; Vásquez *et al.* 2013).
Generalist vs. specialist – food and habitat	Feeds on fish, and more commonly on anchovy, as well as on crustaceans (e.g., euphausiids); it also feeds on copepods, isopods and cephalopods (Mejía *et al.* 1970). Found along the coast and in oceanic waters from 10 to 300 m depth (Smith-Vaniz *et al.* 2010).
Biomass	The total biomass of *T. murphyi* off Parachique, south of Punta La Negra, Trujullo, Huarmey, Callao and between Chala and Mollendo was 724,912 t in summer 2006 (Castillo *et al.* 2009). In the Tumbes-Tacna zone the biomass was 70,074 t in 2009 (PRODUCE-IMARPE 2009). On a global scale, according to the IUCN Red List this species is in the category UNCERTAIN (Smith-Vaniz *et al.* 2010), i.e., there are not enough data.
Distribution	
Capacity for larval dispersal or larval duration – hatching to settlement (benthic species), hatching to yolk sac re-adsorption (pelagic species).	In central Chile, it is estimated that the eggs and larvae are transported up to 1,800 km offshore, between spawning and nursery areas (Smith-Vaniz *et al.* 2010; Vásquez *et al.* 2013).
Capacity for adult/juvenile movement – lifetime range post-larval stage.	
Physiological tolerance – latitudinal coverage of adult species as a proxy of environmental tolerance.	Distributed from Ecuador to the south of Chile (Smith-Vaniz *et al.* 2010); approximately 45° of latitudinal coverage. 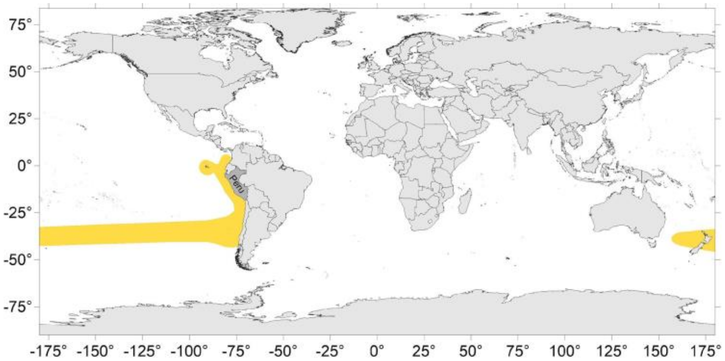
Spatial availability of unoccupied habitat for most critical life stage – ability to shift distributional range.	Approximately 27° of latitude may be available to the south of Peru.
Phenology	

Environmental variable as a phenological cue for spawning or breeding – cues include salinity, temperature, currents, and freshwater flows.	Not available.
Environmental variable as a phenological cue for settlement or metamorphosis	Not available.
Temporal mismatches of life-cycle events – duration of spawning, breeding or moulting season.	The reproductive season is from August to November and in February, but its greatest reproductive peak occurs at the end of winter (Mejía *et al.* 1970); duration of spawning is thus assumed to last > 4 months.
Migration (seasonal and spawning)	Not available.
Exposure	
Response to environmental variability	The landing of this species has increased during El Niño events. Possible distributional changes are also anticipated; for example, the horse mackerel was distributed throughout the coast until El Niño 1997–1998, and then it moved to the south (Gutiérrez *et al.* 2012).
References	

Castillo, R., Gutiérrez, M., Peraltilla, S., & Escudero, L. Distribución y biomasa de los principales recursos pelágicos del mar peruano. Verano 2006. *Inf. Inst. Mar Perú* **36**, 121–130 (2009).

Dioses, T., Alarcón, V.H., Nakama, M.E., & Echevarría, A. Desarrollo ovocitario, fecundidad parcial y distribución vertical de los cardúmenes en desove del jurel *Trachurus murphyi* (N). In: Memorias del Simposio Internacional de los Recursos Vivos y las Pesquerías en el Pacífico Sudeste: 287 – 294 (1988).

Gutiérrez, M., Castillo, R., Segura, M., Peraltilla, S., & Flores, M. Trends in spatio-temporal distribution of Peruvian anchovy and other small pelagic fish biomass from 1966-2009. *Lat. Am. J. Aquat. Res.* **40**, 633–648 (2012).

Marzloff, M., Shin, Y.J., Tam, J., Travers, M., & Bertrand, A. Trophic structure of the Peruvian marine ecosystem in 2000–2006: Insights on the effects of management scenarios for the hake fishery using the IBM trophic model Osmose. *J. Mar. Syst.* **75**, 290–304 (2009).

Mejía, J., Samamé, M., & Pastor, A. Información básica de los principales peces de consumo. *Instituto del Mar, Serie de Informes Especiales IM–62*. pp 29 (1970).

PRODUCE-IMARPE. Prospección bioceanográfica de los recursos jurel y caballa – 2009. Informe PRODUCE-IMARPE. pp 45 (2009).

Smith-Vaniz, B., Robertson, R., & Dominici-Arosemena, A. *Trachurus murphyi*. The IUCN Red List of Threatened Species 2010: e.T183965A8207652. Available at: http://dx.doi.org/10.2305/IUCN.UK.2010-3.RLTS.T183965A8207652.en (2010).

Vásquez, S., Correa-Ramírez, M., Parada, C., & Sepúlveda, A. The influence of oceanographic processes on jack mackerel (*Trachurus murphyi*) larval distribution and population structure in the southeastern Pacific Ocean. *ICES J. Mar. Sci.* **70**, 1097–1107 (2013).

1.20 Common dolphinfish - *Coryphaena hippurus*

Sensitivity	
Abundance	
Fecundity – egg production (total fecundity)	Fecundity is estimated between 180,000 and 800,000 hydrated oocytes (Solano-Sare *et al.* 2008).
Recruitment period – successful recruitment event that sustains the abundance of the fishery	The recruitment to the fishery is annual, however good recruitments occurred in 2004, 2007, 2011, i.e., about every 3 years depending on the environmental conditions (Ñiquen 2015).
Average age at maturity	This species reaches sexual maturity at 50–90 cm in length; in Peru it reaches maturity at 90 cm, in the first 2 years of life (Solano-Sare *et al.* 2008; Florida Museum 2021).
Generalist vs. specialist – food and habitat	Feeds on various fishes, as well as on zooplankton, crustaceans and cephalopods (Solano-Sare *et al.* 2008). Inhabits open waters and coastal areas from the surface to 85 m depth (Collette *et al.* 2011).
Biomass	The biomass was estimated between 10,000 and 40,000 t from 2007 to 2014, with projections for continuous increase in biomass to 2019 (Valero *et al.* 2016). According to the IUCN Red List this species is in the category LEAST CONCERN (LC) (Collette *et al.* 2011), which means that it is abundant and widely distributed.
Distribution	
Capacity for larval dispersal or larval duration – hatching to settlement (benthic species), hatching to yolk sac re-adsorption (pelagic species).	Not available.

Capacity for adult/juvenile movement – lifetime range post-larval stage.	This species migrates to the coast due to movements of warm oceanic waters towards the continent and during El Niño (Solano-Sare *et al.* 2008).
Physiological tolerance – latitudinal coverage of adult species as a proxy of environmental tolerance.	Cosmopolitan species distributed in the United States, Mexico, throughout Peru (Zorritos, Mancora, Cabo Blanco, Paita, Huacho, Callao, Vila Vila) and as far south as Antofagasta, Chile, as well as in the Galapagos Islands (Collette *et al.* 2011); approximately 48° of latitudinal coverage. 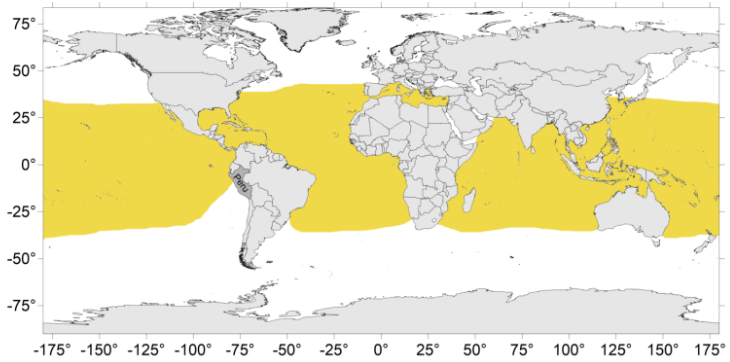
Spatial availability of unoccupied habitat for most critical life stage – ability to shift distributional range.	Approximately 5° of latitude may be available to the south of Peru; approximately 35° of latitude may be available to the north of Peru.
Phenology	
Environmental variable as a phenological cue for spawning or breeding – cues include salinity, temperature, currents, and freshwater flows.	The eggs seem to have a thermal preference around 25°C (Solano-Sare *et al.* 2008).
Environmental variable as a phenological cue for settlement or metamorphosis	Not available.
Temporal mismatches of life-cycle events – duration of spawning, breeding or moulting season.	In Peru mature individuals are found throughout the year, and more frequently in the austral summer from December to March (Solano-Sare *et al.* 2008; Florida Museum 2021); duration of spawning is thus assumed to last > 4 months.
Migration (seasonal and spawning)	This species migrates to the coast due to movements of warm oceanic waters towards the continent and during El Niño (Solano-Sare *et al.* 2008).
Exposure	

Response to environmental variability	In Peru this species is associated with the intrusion of surface subtropical waters and it is mainly found in waters at 21–28°C (Ñiquen 2014). The eggs seem to have a thermal preference around 25°C. The catches of this species have increased during El Niño years; after El Niño 1997–1998, catches increased due to the intrusion of warm waters to the Peruvian coast (Solano-Sare *et al.* 2008). This species has high metabolic rates and requires high concentrations of oxygen, therefore, with the shoaling of the minimal oxygen zone its habitat is likely to be reduced vertically (Solano-Sare *et al.* 2008).

References

Collette, B. *et al.* *Coryphaena hippurus*. The IUCN Red List of Threatened Species 2011: e.T154712A4614989. Available at: http://dx.doi.org/10.2305/IUCN.UK.2011-2.RLTS.T154712A4614989.en (2011).

Florida Museum. Available at: https://www.flmnh.ufl.edu/fish/discover/species-profiles/coryphaena-hippurus/ (2021).

Ñiquen, M. Panorama general de las investigaciones del perico (*Coryphaena hippurus*) en Perú. Available at: http://docplayer.es/29419892-Panorama-general-de-las-investigaciones-del-perico-coryphaena-hippurus-en-peru.html (2014).

Ñiquen, M. Panorama general de las investigaciones del perico (*Coryphaena hippurus*) en Perú con énfasis en el periodo 2014–2015. Available at: https://www.iattc.org/Meetings/Meetings2015/OctDorado/pdfs/presentations/DOR-2-Investigaciones-del-Perico-en-Peru.pdf (2015).

Solano-Sare, A., *et al*. Biología y pesquería del perico. *IMARPE*. Callao, Perú. pp 23 (2008).

Valero, J.L. *et al.* Exploratory Management Strategy Evaluation (MSE) of Dorado (*Coryphaena hippurus*) in the Southeastern Pacific Ocean. *7th Meeting of the IATTC Scientific Advisory Meeting*. La Jolla, California. 9–15 May 2016. Available at: https://www.iattc.org/Meetings/Meetings2016/SAC7/PDFfiles/presentations/SAC-07-06a(ii)-MSE-for-dorado.pdf (2016).

1.21 Eastern Pacific bonito - *Sarda chiliensis chiliensis*

Sensitivity

Abundance	
Fecundity – egg production (total fecundity)	Average partial fecundity is around 499,550 oocytes per spawning batch (Yoshida 1980).

Recruitment period – successful recruitment event that sustains the abundance of the fishery	Given that major spawning peaks occur annually and sexual maturity is reached at one year of age, it is assumed that recruitment also occurs annually, i.e., recruitment is consistent every 1–2 years.
Average age at maturity	This species reaches maturity at one year of age (Yoshida 1980).
Generalist vs. specialist – food and habitat	In the area of Lambayeque, Peru it feeds mainly on anchovy (Llanos *et al.* 2009).
Biomass	According to the IUCN Red List this species is in the category LEAST CONCERN (LC) (Collette *et al.* 2011), which means that it is abundant and widely distributed.
Distribution	
Capacity for larval dispersal or larval duration – hatching to settlement (benthic species), hatching to yolk sac re-adsorption (pelagic species).	Larval growth is slow between 2 and 4 days after hatching, which coincides with the opening of the mouth, the depletion of the yolk sac, and the initiation of external feeding (2.3 days) (Miranda *et al.* 2014).
Capacity for adult/juvenile movement – lifetime range post-larval stage.	Not available.
Physiological tolerance – latitudinal coverage of adult species as a proxy of environmental tolerance.	In South America this species occurs from Puerto Pizarro, Peru to Talcahuano, Chile (Chirichigno & Cornejo 2001); approximately 33° of latitudinal coverage.

	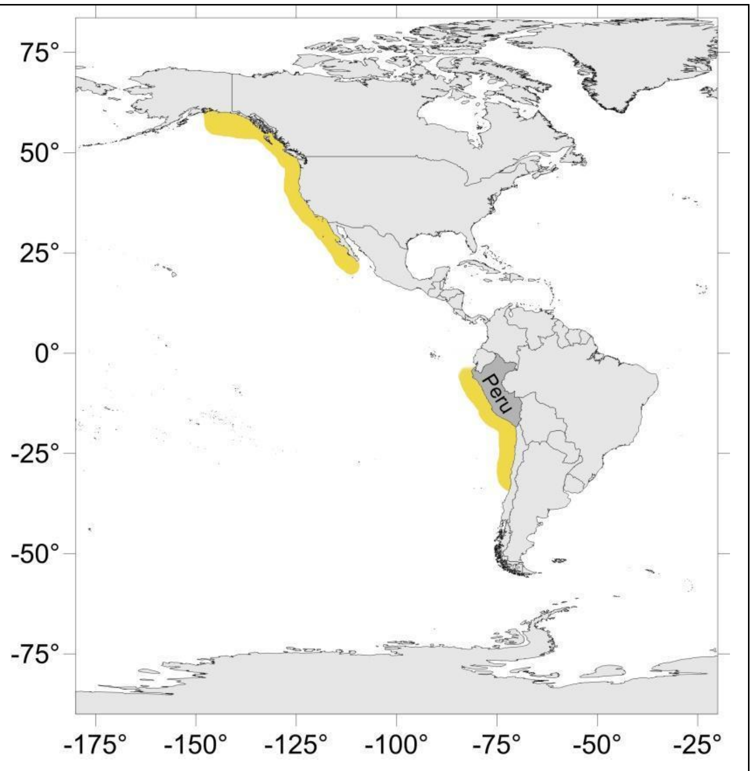
Spatial availability of unoccupied habitat for most critical life stage – ability to shift distributional range.	Approximately 19° of latitude may be available to the south of Peru.
Phenology	
Environmental variable as a phenological cue for spawning or breeding – cues include salinity, temperature, currents, and freshwater flows.	The main spawning peak occurs when positive thermal anomalies are expected (Llanos *et al.* 2009). In the Northeast Pacific, spawning occurs between 22°C and 26°C (Miranda *et al.* 2014).
Environmental variable as a phenological cue for settlement or metamorphosis	Not available.
Temporal mismatches of life-cycle events – duration of spawning, breeding or moulting season.	Its main spawning peak is between December and February (Llanos *et al.* 2009); duration of spawning is thus assumed to last 2–4 months.
Migration (seasonal and spawning)	Has seasonal changes in the use of the habitat; it is more common near the coast in summer and at the beginning of autumn than during winter. These changes in distribution are attributed to long migrations in search of prey (Allen *et al.* 2006).

**Exposure**	
Response to environmental variability	The recurrence of warm conditions in the months of February, June, July and December seem to be associated with the presence of bonito (Llanos *et al.* 2009). However, the landing of this species has declined during El Niño events. The main spawning peak occurs when positive thermal anomalies are expected (Llanos *et al.* 2009). In the Northeast Pacific, spawning occurs between 22°C and 26°C (Miranda *et al.* 2014).

References

Allen, L.G., Pondella, D.J., & Horn, M.H. The ecology of marine fishes. California and adjacent waters. University of California Press. pp 670 (2006).

Chirichigno, N., & Cornejo, R.M. Catálogo comentado de los peces marinos del Perú. *Inst. Mar Perú*. Callao, Perú (2001).

Collette, B. *et al. Sarda chiliensis*. The IUCN Red List of Threatened Species 2011: e.T170352A6763952. Available at: http://dx.doi.org/10.2305/IUCN.UK.2011-2.RLTS.T170352A6763952.en (2011).

Llanos, J. *et al.* Investigaciones de IMARPE-Sede Lambayeque durante 2009. pp 89 (2009).

Miranda, L., Vilaxa, A., Ávila, R., & Rodríguez, M. Caracterización del ciclo de desarrollo embrionario y larval de *Sarda chiliensis chiliensis* (Alva, 1987) en un sistema acuícola de recirculación (SAR). *Int. J. Morphol.* **32**, 1492–1501 (2014).

Yoshida, H.O. Synopsis of biological data on bonitos of the genus Sarda (1980). In: Miranda, L., Vilaxa, A., Ávila, R., Rodríguez, M. Caracterización del ciclo de desarrollo embrionario y larval de *Sarda chiliensis chiliensis* (Alva, 1987) en un sistema acuícola de recirculación (SAR). *Int. J. Morphol.* **32**: 1492–1501 (2014).

1.22 Jumbo flying squid - *Dosidicus gigas*

**Sensitivity**	
Abundance	
Fecundity – egg production (total fecundity)	In Peru the potential fecundity has been estimated in up to 25 million eggs in females of 1 m of mantle length (Sanchez 2010, unpublished). Nigmatullin *et al.* (2001) has reported up to 32 million eggs produced by large females.
Recruitment period – successful recruitment event that sustains the abundance of the fishery	Recruitment events are likely consistent every 1–2 years.

Average age at maturity	In the Mexican Pacific this species reaches sexual maturity at 205–350 days of age (Mejía-Rebollo *et al.* 2008).
Generalist vs. specialist – food and habitat	Prey belonging to up to 55 taxa have been found in stomach contents of *D. gigas* but stomachs also can contain only one or two taxa in Peruvian waters (Alegre *et al.* 2014). It feeds mainly on myctophids, squids and crustaceans in the Gulf of California (Markaida *et al.* 2008); in juvenile stage up to 9 types of prey have been detected (Camarillo-Coop *et al.* 2013).
Biomass	The biomass of jumbo squid increased from 200,000 to 900,000 t from 2002 to 2006 (Argüelles & Tafur 2010). However, according to the IUCN Red List this species is in the category UNCERTAIN (Barratt & Allcock 2014), i.e., there are insufficient data.
Distribution	
Capacity for larval dispersal or larval duration – hatching to settlement (benthic species), hatching to yolk sac re-adsorption (pelagic species).	Not available.
Capacity for adult/juvenile movement – lifetime range post-larval stage.	Adults can perform horizontal movements of ~ 35 km/day (Gilly *et al.* 2006; Stewart *et al.* 2012). The jumbo squid is likely to carry out considerable migrations in Peruvian waters (Keyl *et al.* 2008).
Physiological tolerance – latitudinal coverage of adult species as a proxy of environmental tolerance.	Oceanic-neritic species that is distributed from California to southern Chile, although its distribution has increased latitudinally in the northern and southern hemispheres (Nigmatullin *et al.* 2001; Keyl *et al.* 2008; Ramos *et al.* 2017); approximately 107° of latitudinal coverage.

	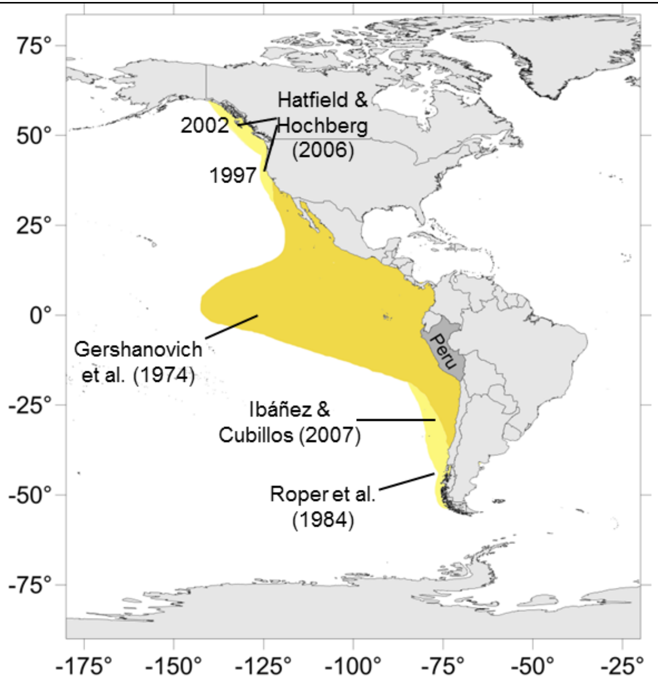
Spatial availability of unoccupied habitat for most critical life stage – ability to shift distributional range.	Approximately 32° of latitude may be available to the south of Peru; approximately 62° of latitude may be available to the north of Peru.
Phenology	
Environmental variable as a phenological cue for spawning or breeding – cues include salinity, temperature, currents, and freshwater flows.	Temperature is a determinant variable for spawning; paralarvae tolerate a limited range of temperatures, probably between 17°C and 23°C, although the embryos and paralarvae may be able to tolerate temperatures as low as 15°C (Staaf *et al.* 2011). The concentration of nutrients also may favor the recently hatched paralarvae.
Environmental variable as a phenological cue for settlement or metamorphosis	Not available.
Temporal mismatches of life-cycle events – duration of spawning, breeding or moulting season.	The main spawning peak of *Dosidicus gigas* is from October to January, and a smaller spawning peak from July to August in Peruvian waters although there are mature females throughout the year (Tafur *et al.* 2001); duration of spawning is thus assumed to last > 4 months.
Migration (seasonal and spawning)	Spawning occurs on both the continental shelf and adjacent oceanic areas (Nigmatullin *et al.* 2001), as well as on the slope of the Nazca submarine mountain (Tafur *et al.* 2001).
Exposure	

Response to environmental variability	The increase in temperature during El Niño has a negative effect on the catches of *D. gigas*, while low temperatures during La Niña have a positive effect (Ibáñez *et al.* 2016). High temperatures tend to accelerate the rate of growth and decrease the life span, which may be positive as the population turnover would occur more often. However, also there would be negative effects; for instance, the size of the eggs would decrease, the size of sexual maturity would be reached at smaller sizes, and therefore the population structure would change. Individuals would require more food because of increased metabolic demand, and thus would require more oxygen. The increase in temperature also would cause the mismatch of the reproductive cycle and of recruitment (Pecl & Jackson 2008; Hoving *et al.* 2013).<br><br>The blood of *D. gigas* has little capacity to carry oxygen; these squids use all the oxygen they carry in their blood, even when resting. The oxygen-blood ratio is highly sensitive to pH, a property that facilitates the release of oxygen to the muscles but presumably interferes with oxygen extraction in hypoxic or CO_2-rich waters. *Dosidicus gigas* is then vulnerable to ocean acidification, oceanic warming and hypoxia (Rosa & Seibel 2008).<br><br>With climate change the oceans are warming and an expansion of the minimal oxygen zone is expected to occur. If *D. gigas* does not adapt or perform horizontal migrations, the synergy between ocean acidification, ocean warming, and the expanding hypoxic conditions will compress the range of habitable depth for this species (Rosa & Seibel 2008).<br><br>Temperature is a determinant variable for spawning; paralarvae tolerate a limited range of temperatures, probably between 17°C and 23°C, although the embryos and paralarvae may be able to tolerate temperatures as low as 15°C (Staaf *et al.* 2011). The concentration of nutrients also may favor the recently hatched paralarvae.

References

Alegre, A. *et al.* Comprehensive model of jumbo squid *Dosidicus gigas* trophic ecology in the Northern Humboldt Current System. *PLoS ONE* **9**, e85919 (2014).

Argüelles, J., & Tafur, R. New insights on the biology of the jumbo squid *Dosidicus gigas* in the Northern Humboldt Current System: Size at maturity, somatic and reproductive investment. *Fish. Res.* **106**, 185–192 (2010).

Barratt, I., & Allcock, L. *Dosidicus gigas*. The IUCN Red List of Threatened Species 2014: e.T162959A958088. Available at: http://dx.doi.org/10.2305/IUCN.UK.2014-1.RLTS.T162959A958088.en (2014).

Camarillo-Coop, S., Salinas-Zavala, C.A., Lavaniegos, B.E., & Markaida, U. Food in early life stages of *Dosidicus gigas* (Cephalopoda: Ommastrephidae) from the Gulf of California, Mexico. *J. Mar. Biol. Assoc. UK* **93**, 1903–1910 (2013).

Gershanovich, D.E., Natarov, V.V., & Cherny, E.I. Oceanologic bases as the forming up of the increased production areas in the Pacific Ocean. *Trudy VNIRO* **98**, 35–42 (1974).

Gilly, W.F. *et al.* Vertical and horizontal migrations by the jumbo squid *Dosidicus gigas* revealed by electronic tagging. *Mar. Ecol. Prog. Ser.* **324**, 1–17 (2006).

Hatfield, E.M.C., & Hochberg, F. *Dosidicus gigas*: northern range extension events. In: Olson, B., Young, J. (eds.) *The Role of Squid in Pelagic Marine Ecosystems*—GLOBEC-CLIOTOP WG3 Workshop, University of Hawaii, Manoa (2006).

Hoving, H.J. *et al.* Extreme plasticity in life-history strategy allows a migratory predator (jumbo squid) to cope with a changing climate. *Glob. Chang. Biol.* **19**, 2089–2103 (2013).

Ibáñez, C., & Cubillos, L.A. Seasonal variation in the length structure and reproductive condition of the jumbo squid *Dosidicus gigas* (d'Orbigny, 1835) off central-south Chile. *Sci. Mar.* **71**, 123–128 (2007).

Ibáñez, C.M. *et al.* Population dynamics of the squids *Dosidicus gigas* (Oegopsida: Ommastrephidae) and *Doryteuthis gahi* (Myopsida: Loliginidae) in northern Peru. *Fish. Res.* **173**, 151–158 (2016).

Keyl, F. *et al.* A Hypothesis on Range Expansion and Spatio-Temporal Shifts in Size-at-Maturity of Jumbo Squid (*Dosidicus gigas*) in the Eastern Pacific Ocean. *CalCOFI Rep.* **49**, 119–128 (2008).

Markaida, U., Gilly, W.F., Salinas-Zavala, C.A., Rosas-Luis, R., & Booth, J.A.T. Food and feeding of jumbo squid *Dosidicus gigas* in the Central Gulf of California During 2005–2007. *CalCOFI Rep.* **49**, 90–103 (2008).

Mejía-Rebollo, A., Quiñonez-Velázquez, C., Salinas-Zavala, C., & Markaida, U. Age, growth and maturity of jumbo squid (*Dosidicus gigas* D'Orbigny, 1835) off the Western Coast of the Baja California peninsula. *CalCOFI Rep.* **49**, 256–262 (2008).

Nigmatullin, C.M., Nesis, K.N., & Arkhipkin, A.I. A review of the biology of the jumbo squid *Dosidicus gigas* (Cephalopoda: Ommastrephidae). *Fish. Res.* **54**, 9–19 (2001).

Pecl, G.T., & Jackson, G.D. The potential impacts of climate change on inshore squid: biology, ecology and fisheries. *Rev. Fish Biol. Fisheries* **18**, 373–385 (2008).

Ramos, J.E. *et al.* Characterization of the northernmost spawning habitat of *Dosidicus gigas* with implications on its northwards range extension. *Mar. Ecol. Prog. Ser.* **572**, 179–192 (2017).

Roper, C.F.E., Sweeney, M.J., & Nauen, C.E. FAO Species catalogue Vol. 3. Cephalopods of the world. An Annotated and Illustrated Catalogue of Species of Interest to Fisheries. FAO Fisheries Synopsis No. 125. (1984).

Rosa, R., & Seibel, B.A. Synergistic effects of climate-related variables suggest future physiological impairment in a top oceanic predator. *Proc. Natl. Acad. Sci. USA* **105**, 20776–20780 (2008).

Staaf, D.J., Zeidberg, L.D., & Gilly, W.F. Effects of temperature on embryonic development of the Humboldt squid *Dosidicus gigas*. *Mar. Ecol. Prog. Ser.* **441**, 165–175 (2011).

Stewart, J.S., Hazen, E.L., Foley, D.G., Bograd, S.J., & Gilly, W.F. Marine predator migration during range expansion: Humboldt squid *Dosidicus gigas* in the northern California Current System. *Mar. Ecol. Prog. Ser.* **471**, 135–150 (2012).

Tafur, R., Villegas, P., RabÍ, M., & Yamashiro, C. Dynamics of maturation, seasonality of reproduction and spawning groulds of the jumbo squid *Dosidicus gigas* (Cephalopoda: Ommastrephidae) in Peruvian waters. *Fish. Res.* **54**, 33–50 (2001).

1.23 Mote sculpin - *Normanichthys crockeri*

Sensitivity	
Abundance	
Fecundity – egg production (total fecundity)	Not available.
Recruitment period – successful recruitment event that sustains the abundance of the fishery	Recruitment events are likely consistent every 1–2 years.
Average age at maturity	Average size of sexual maturity of females is 8.5 cm of total length (Quiroz *et al.* 1996). Given that this species has a short life cycle (Landaeta *et al.* 2010), it is estimated that sexual maturity is reached before two years of life.
Generalist vs. specialist – food and habitat	Adults feed on calanoid copepods, amphipods and crustacean's larvae in equal proportions; inhabits the pelagic-coastal zone in cold and temperate waters (Quiroz *et al.* 1996).
Biomass	The total biomass of this species during summer 2006 off Supe, Pisco and Chala, Peru was 92,741 t (Castillo *et al.* 2009).
Distribution	

Capacity for larval dispersal or larval duration – hatching to settlement (benthic species), hatching to yolk sac re-adsorption (pelagic species).	The larvae feed on the yolk sac for up to 6 days (Landaeta *et al.* 2010).
Capacity for adult/juvenile movement – lifetime range post-larval stage.	Not available.
Physiological tolerance – latitudinal coverage of adult species as a proxy of environmental tolerance.	Occurs from Chimbote, Peru to Moche island, Chile (Quiroz *et al.* 1996); approximately 29° of latitudinal coverage. 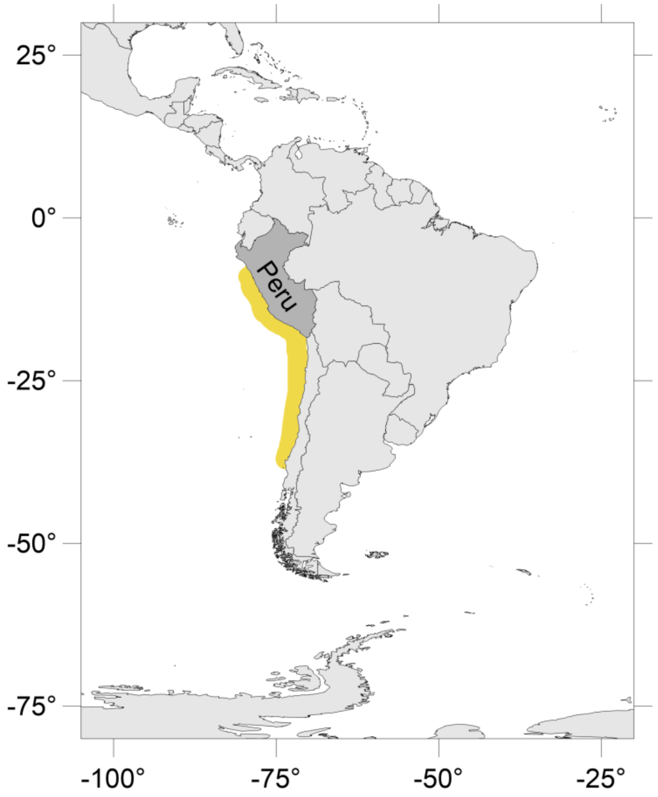
Spatial availability of unoccupied habitat for most critical life stage – ability to shift distributional range.	Approximately 20° of latitude may be available to the south of Peru.
Phenology	
Environmental variable as a phenological cue for spawning or breeding – cues include salinity, temperature, currents, and freshwater flows.	Not available.

Environmental variable as a phenological cue for settlement or metamorphosis	Not available.
Temporal mismatches of life-cycle events – duration of spawning, breeding or moulting season.	Spawning occurs in winter in southern Peru (Quiroz *et al.* 1996). In Chile, the highest spawning peak occurs at the end of summer (February and March) with lower spawning peaks during winter and spring (Landaeta *et al.* 2010); duration of spawning is thus assumed to last 2–4 months.
Migration (seasonal and spawning)	Not available.
Exposure	
Response to environmental variability	The availability and concentration of the mote sculpin along the coast of southern Peru is associated with the intrusion of subantarctic temperate waters and intense phytoplankton blooms, especially off Picata and Ite. At lower temperatures catches increase; for example, temperatures between 14°C and 15°C appear to be favorable (Quiroz *et al.* 1996).
References	

Castillo, R., Gutiérrez, M., Peraltilla, S., & Escudero, L. Distribución y biomasa de los principales recursos pelágicos del mar peruano. Verano 2006. *Inf. Inst. Mar Perú* **36**, 121–130 (2009).

Landaeta, M.F., Inostroza, P.A., Ramirez, A., Soto-Mendoza, S., & Castro, L.R. Distribution patterns, larval growth and hatch dates of early stages of the mote sculpin *Normanichthys crockeri* (Scorpaeniformes, Normanichthyidae) in the upwelling ecosystem off central Chile. *Rev. Biol. Mar. Oceanogr.* **45**, 575–588. (2010).

Quiroz, M., Zambrano, M., & Cárdenas, F. El recurso camotillo (*Normanichthys crockeri*) en la zona sur del Perú. Abril 1995 a Enero 1996. *Inf. Prog. Inst. Mar Perú* **46**, 19–28 (1996).

1.24 Pacific chub mackerel - *Scomber japonicus*

Sensitivity	
Abundance	
Fecundity – egg production (total fecundity)	Its partial fecundity has been estimated in 78,174 oocytes per spawning batch (Peña *et al.* 1986).
Recruitment period – successful recruitment event that sustains the abundance of the fishery	Recruitment events are likely consistent every 1–2 years.

Average age at maturity	This species reaches sexual maturity at 2 years (Marzloff *et al.* 2009).
Generalist vs. specialist – food and habitat	Feeds on copepods, euphausiidae, fish eggs; among the teleosts it feeds mainly on anchovy and also has cannibalistic habits (Mejía *et al.* 1970). This is a pelagic and coastal species that occurs as deep as 300 m depth (Collette *et al.* 2011).
Biomass	The total biomass off Trujillo and Huacho, Chimbote, Huarmey, Supe, Atico and Mollendo in Peru was estimated at 225,645 t (Castillo *et al.* 2009). The estimated biomass in 2009 in Tumbes-Tacna was 131,866 t, in Mancora-Huarmey it was estimated at 125,214 t, and in Salaverry-Atico it was estimated at 65,171 t (PRODUCE-IMARPE 2009). According to the IUCN Red List this species is in the category LEAST CONCERN (LC) (Collette *et al.* 2011), which means that it is abundant and widely distributed.
Distribution	
Capacity for larval dispersal or larval duration – hatching to settlement (benthic species), hatching to yolk sac re-adsorption (pelagic species).	It presents a larval stage that consumes its yolk sac at approximately 46 hours after spawning (Hunter & Kimbrel 1980).
Capacity for adult/juvenile movement – lifetime range post-larval stage.	Not available.
Physiological tolerance – latitudinal coverage of adult species as a proxy of environmental tolerance.	In the Pacific off South America, this species occurs from Costa Rica and along the Peruvian coast to Valparaíso, Chile and the Galapagos Islands (Mejía *et al.* 1970; Collette *et al.* 2011); approximately 45° of latitudinal coverage.<br>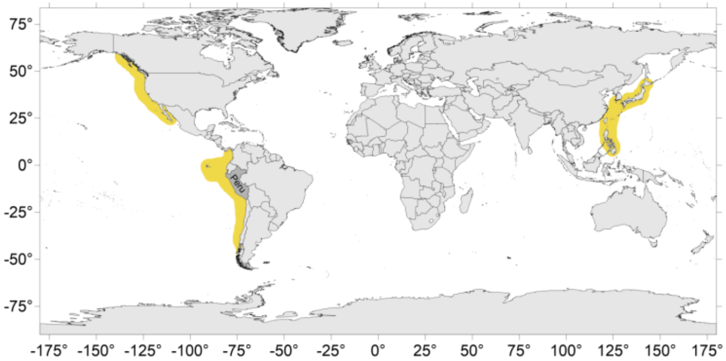
Spatial availability of unoccupied habitat for most critical life stage – ability to shift distributional range.	Approximately 27° of latitude may be available to the south of Peru.

Phenology	
Environmental variable as a phenological cue for spawning or breeding – cues include salinity, temperature, currents, and freshwater flows.	Spawning generally occurs between 15°C and 20°C (Collette *et al.* 2011). The reproductive season appears to occur earlier in the year due to the positive thermal anomalies (Llanos *et al.* 2009).
Environmental variable as a phenological cue for settlement or metamorphosis	Metamorphosis of larvae appears to be stimulated by the effects of temperature, e.g., metamorphosis occurs in 24 days at 16.8°C or in 16 days at higher temperatures (22.1°C) (Hunter & Kimbrell 1980).
Temporal mismatches of life-cycle events – duration of spawning, breeding or moulting season.	The main spawning peaks occur between April and May, and a secondary peak in November (Llanos *et al.* 2009); duration of spawning is thus assumed to last 2–4 months.
Migration (seasonal and spawning)	Not available.
Exposure	
Response to environmental variability	This species changed in distribution to the south where juveniles dominated, and there was an increase in the reproductive events, in biomass and in captures in Peru during El Niño 1997–1998 (Ñiquen & Bouchon 2004; Llanos *et al.* 2009). Spawning generally occurs between 15°C and 20°C (Collette *et al.* 2011). The metamorphosis of the larva appears to be stimulated by the effects of temperature, e.g. metamorphosis occurs in 24 days at 16.8°C or in 16 days at higher temperatures (22.1°C) (Hunter & Kimbrell 1980). The reproductive season appears to occur earlier in the year due to the positive thermal anomalies (Llanos *et al.* 2009).

References

Castillo, R., Gutiérrez, M., Peraltilla, S., & Escudero, L. Distribución y biomasa de los principales recursos pelágicos del mar peruano. Verano 2006. *Inf. Inst. Mar Perú* **36**, 121–130 (2009).

Collette, B. *et al. Scomber japonicus*. The IUCN Red List of Threatened Species 2011: e.T170306A6737373. Available at: http://dx.doi.org/10.2305/IUCN.UK.2011-2.RLTS.T170306A6737373.en (2011).

Hunter, J.R., & Kimbrell, C.A. Early life history of Pacific mackerel, *Scomber japonicus*. *Fish. Bull.* **78**, 89–101 (1980).

Llanos, J. *et al.* Investigaciones de IMARPE-Sede Lambayeque durante 2009. pp 89 (2009).

Marzloff, M., Shin, Y.J., Tam, J., Travers, M., & Bertrand, A. Trophic structure of the Peruvian marine ecosystem in 2000–2006: Insights on the effects of management scenarios for the hake fishery using the IBM trophic model Osmose. *J. Mar. Syst.* **75**, 290–304 (2009).

Mejía, J., Samamé, M., & Pastor, A. Información básica de los principales peces de consumo. *Instituto del Mar, Serie de Informes Especiales IM–62*. pp 29 (1970).

Ñiquen, M., & Bouchon, M. Impact of El Niño events on pelagic fisheries in Peruvian waters. *Deep Sea Res. Part II Top. Stud. Oceanogr.* **51**, 563–574 (2004).

Peña, N., Alheit, J., & Nakama, M.E. Fecundidad partial de la caballa del Peru (*Scomber Japonicus peruanus*). *Bol. Inst. Mar Perú* **10**: 91–104 (1986).

PRODUCE-IMARPE. Prospección bioceanográfica de los recursos jurel y caballa – 2009. Informe PRODUCE-IMARPE. pp 45 (2009).

1.25 Pacific sardine - *Sardinops sagax*

Sensitivity	
Abundance	
Fecundity – egg production (total fecundity)	Its partial fecundity is 55,000 oocytes per spawning batch (Lo *et al.* 1986).
Recruitment period – successful recruitment event that sustains the abundance of the fishery	The recruitment to the fishery is annual (Serra & Tsukuyama 1988).
Average age at maturity	It reaches sexual maturity at 2 years (Marzloff *et al.* 2009).
Generalist vs. specialist – food and habitat	Feeds on diatoms, dinoflagellates, copepods, nauplii and fish eggs, among others (Mejía *et al.* 1970). Inhabits in the neritic zone from 0 to 200 m depth (Froese & Pauly 2021).
Biomass	The biomass has fluctuated drastically in Peru; at the beginning of 1983 the biomass was 5.5 million t and in winter of 1983 it was 2 million t (Serra & Tsukuyama 1988). According to the IUCN Red List, at the global level this species is in the category LEAST CONCERN (LC) (Iwamoto & Eschmeyer 2010), which means that it is abundant and widely distributed.
Distribution	
Capacity for larval dispersal or larval duration – hatching to settlement	This species has a pelagic larval phase (Froese & Pauly 2021) that in African waters lasts 50–100 days (Shannon 1998).

(benthic species), hatching to yolk sac re-adsorption (pelagic species).	
Capacity for adult/juvenile movement – lifetime range post-larval stage.	There are records of horizontal movements up to 754 nautical miles (1,396 km) in 175 days (Serra & Tsukuyama 1988).
Physiological tolerance – latitudinal coverage of adult species as a proxy of environmental tolerance.	It is a cosmopolitan species that in Pacific off South America occurs off Ecuador, Galapagos Islands, throughout Peru and Chile (Chirichigno & Cornejo 2001); approximately 116° of latitudinal coverage. 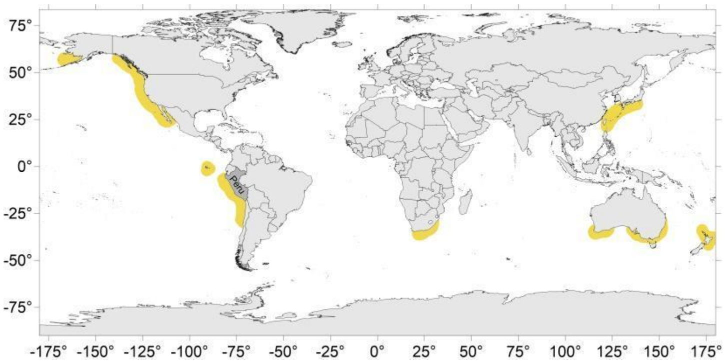
Spatial availability of unoccupied habitat for most critical life stage – ability to shift distributional range.	Approximately 41° of latitude may be available to the south of Peru; approximately 62° of latitude may be available to the north of Peru.
Phenology	
Environmental variable as a phenological cue for spawning or breeding – cues include salinity, temperature, currents, and freshwater flows.	In the California Current system, this species has a high degree of flexibility in spawning events associated with environmental variability due to El Niño and La Niña (Weber & McClatchie 2010; Song *et al.* 2012).
Environmental variable as a phenological cue for settlement or metamorphosis	Not available.
Temporal mismatches of life-cycle events – duration of spawning, breeding or moulting season.	Presents an extended spawning event that lasts approximately 9 months, from July to March. There is a main spawning peak in August-September and a smaller one in February-March (Serra & Tsukuyama 1988).
Migration (seasonal and spawning)	Migrates to the coast in search of food and also carries out migrations into the sea during the spawning season (Mejía *et al.* 1970).

**Exposure**	
Response to environmental variability	The sardine changed in distribution to the south, where juveniles were common and there was an increase in reproductive events, biomass and landings in Peru during El Niño 1997–1998 (Ñiquen & Bouchon 2004). In the California Current system, this species has a high degree of flexibility in spawning events associated with environmental variability due to El Niño and La Niña (Weber & McClatchie 2010; Song *et al.* 2012).

References

Chirichigno, N., & Cornejo, R.M. Catálogo comentado de los peces marinos del Perú. *Inst. Mar Perú*. Callao, Perú (2001).

Froese, R., & Pauly, D. (eds.) FishBase. World Wide Web electronic publication. Available at: http://www.fishbase.org/summary/1477 (2021).

Iwamoto, T., & Eschmeyer, W. *Sardinops sagax* ssp. *sagax*. The IUCN Red List of Threatened Species 2010: e.T184056A8229422. Available at: http://dx.doi.org/10.2305/IUCN.UK.2010-3.RLTS.T184056A8229422.en (2010).

Lo, N.C.H., Alheit, J., & Alegre, B. Fecundidad parcial de la sardina peruana. *Bol. Inst. Mar Perú* **10**, 48–60 (1986).

Marzloff, M., Shin, Y.J., Tam, J., Travers, M., & Bertrand, A. Trophic structure of the Peruvian marine ecosystem in 2000–2006: Insights on the effects of management scenarios for the hake fishery using the IBM trophic model Osmose. *J. Mar. Syst.* **75**, 290–304 (2009).

Mejía, J., Samamé, M., & Pastor, A. Información básica de los principales peces de consumo. *Instituto del Mar, Serie de Informes Especiales IM–62*. pp 29 (1970).

Ñiquen, M., & Bouchon, M. Impact of El Niño events on pelagic fisheries in Peruvian waters. *Deep Sea Res. Part II Top. Stud. Oceanogr.* **51**, 563–574 (2004).

Serra, R, & Tsukuyama, I. Sinopsis de datos biológicos y pesqueros de la sardina *Sardinops sagax* (Jenyns, 1842) en el Pacífico Suroriental. *FAO Sinop Pesca* **13**. pp 60 (1988).

Shannon, L.J. Modelling environmental effects on the early life history of the South African anchovy and sardine: A comparative approach. In: Pillar, S.C., Moloney, C.L., Payne, A.I.L., Shillington, F.A. (eds.) Benguela dynamics. *S. Afr. J. mar. Sci.* **19**, 291–304 (1998).

Song, H., *et al.* Application of a data-assimilation model to variability of Pacific sardine spawning and survivor habitats with ENSO in the California Current System. *J. Geophys. Res.* **117**, C03009 (2012).

Weber, E.D., & McClatchie, S. Predictive models of northern anchovy *Engraulis mordax* and Pacific sardine *Sardinops sagax* spawning habitat in the California Current. *Mar. Ecol. Prog. Ser.* **406**, 251–263 (2010).

1.26 Peruvian anchovy - *Engraulis ringens*	
Sensitivity	
Abundance	
Fecundity – egg production (total fecundity)	Fecundity is approximately 8,300 oocytes per spawning batch; spawning occurs approximately every 6 days for three months, which is an approximate total of 12 times (Betsy Buitron, *pers. comm.*).
Recruitment period – successful recruitment event that sustains the abundance of the fishery	The recruitment to the fishery occurs approximately at 8 cm in standard length, and at the age of 5–6 months (Iwamoto *et al.* 2010); hece recruitment events are likely consistent every 1–2 years.
Average age at maturity	Sexual maturity is reached within one year (Marzloff *et al.* 2009).
Generalist vs. specialist – food and habitat	Feeds mainly on diatoms; inhabits waters within 80 km off the coast at maximum depths of 50 m mainly (Iwamoto *et al.* 2010).
Biomass	The parental biomass of summer 2013 was abundant, with 7.6 million adults (IMARPE). According to the IUCN Red List, this species is in the category LEAST CONCERN (LC) (Iwamoto *et al.* 2010), i.e., it is abundant and widely distributed.
Distribution	
Capacity for larval dispersal or larval duration – hatching to settlement (benthic species), hatching to yolk sac re-adsorption (pelagic species).	The larval stage lasts between 32 days and 64 days (Moreno *et al.* 2011).
Capacity for adult/juvenile movement – lifetime range post-larval stage.	It is a migratory species but with limited capacity of movement (IMARPE, unpublished).
Physiological tolerance – latitudinal coverage of adult species as a proxy of environmental tolerance.	Distributed from Ecuador to southern Chile, including the Galapagos island (Iwamoto *et al.* 2010); approximately 59° of latitudinal coverage.

	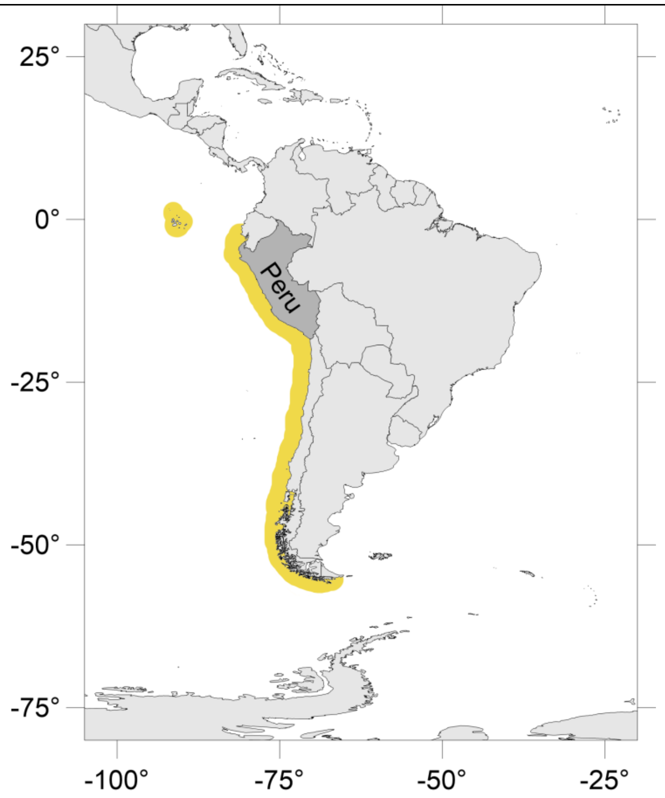
Spatial availability of unoccupied habitat for most critical life stage – ability to shift distributional range.	Approximately 41° of latitude may be available to the south of Peru.
Phenology	
Environmental variable as a phenological cue for spawning or breeding – cues include salinity, temperature, currents, and freshwater flows.	Not available.
Environmental variable as a phenological cue for settlement or metamorphosis	Not available.
Temporal mismatches of life-cycle events – duration of spawning, breeding or moulting season.	Spawning occurs throughout the year on the coasts of Peru, with main peaks in summer and spring (July to September) and with a secondary peak in summer (February and March) (Iwamoto *et al.* 2010); duration of spawning is thus assumed to last > 4 months.
Migration (seasonal and spawning)	Not available.
Exposure	

Response to environmental variability	The landings of this species have decreased during El Niño events; the distribution, size structure and reproductive events of the anchovy in Peru changed during El Niño 1997–1998, where the population moved southwards, adults dominated the size structure, and reproduction was interrupted (Ñiquen & Bouchon 2004).

References

Iwamoto, T., Eschmeyer, W., & Alvarado, J. *Engraulis ringens*. The IUCN Red List of Threatened Species 2010: e.T183775A8174811. Available at: http://dx.doi.org/10.2305/IUCN.UK.2010-3.RLTS.T183775A8174811.en (2010).

Marzloff, M., Shin, Y.J., Tam, J., Travers, M., & Bertrand, A. Trophic structure of the Peruvian marine ecosystem in 2000–2006: Insights on the effects of management scenarios for the hake fishery using the IBM trophic model Osmose. *J. Mar. Syst.* **75**, 290–304 (2009).

Moreno, P., Claramunt, G., & Castro, L.R. Transition period from larva to juvenile in anchoveta *Engraulis ringens*. Length or age related? *J. Fish Biol.* **78**, 825–837 (2011).

Ñiquen, M., & Bouchon, M. Impact of El Niño events on pelagic fisheries in Peruvian waters. *Deep Sea Res. Part II Top. Stud. Oceanogr.* **51**, 563–574 (2004).

1.27 Peruvian silverside - *Odontesthes regia*

Sensitivity

Abundance	
Fecundity – egg production (total fecundity)	Adult females of 4 to 5 year old can spawn between 35,000 and 40,000 eggs (Chura-Cruz *et al.* 2013). In southern Chile, this species is a partial spawner and at each batch it releases 2,051 ± 722 hydrated oocytes (Plaza *et al.* 2011).
Recruitment period – successful recruitment event that sustains the abundance of the fishery	Recruitment in the lake Titicaca occurs between late winter and early summer (IMARPE 2009); recruitment events are thus assumed to be consistent every 1–2 years.
Average age at maturity	The minimum size of sexually mature females is 13.5 cm in length (Mejía *et al.* 1970), although the size at maturity has also been recorded at 23.4 cm (IMARPE 2009). Both sizes are reached in the first and fifth year, respectively (Arrieta *et al.* 2010).
Generalist vs. specialist – food and habitat	Feeds on planktonic organisms and coastal organic detritus near the mouths of the rivers (Mejía *et al.* 1970). Its a pelagic-neritic species that inhabits on sandy bottoms with

	vegetation, as well as in the mouth of rivers; juveniles are found in estuarine environments (Chirichigno & Cornejo 2001; Reis & Lima 2009).
Biomass	In the lake Titicaca, the estimated biomass is 15,320 t (IMARPE 2015; IMARPE-PELT 2015). According to the IUCN Red List this species is in the category LEAST CONCERN (LC) (Reis & Lima 2009), which means that it is abundant and widely distributed.
Distribution	
Capacity for larval dispersal or larval duration – hatching to settlement (benthic species), hatching to yolk sac re-adsorption (pelagic species).	The yolk sack is absorbed at 6–9 days of age (Chirinos & Chuman 1964).
Capacity for adult/juvenile movement – lifetime range post-larval stage.	May carry out reproductive migrations, as has been observed in *O. argentinensis* that migrates in spring-summer to brackish waters of estuaries and lagoons to reproduce (INIDEP 2021).
Physiological tolerance – latitudinal coverage of adult species as a proxy of environmental tolerance.	Occurs from Paita, Peru to Aysén, Chile (Chirichigno & Cornejo 2001; Reis & Lima 2009); approximately 40° of latitudinal coverage. 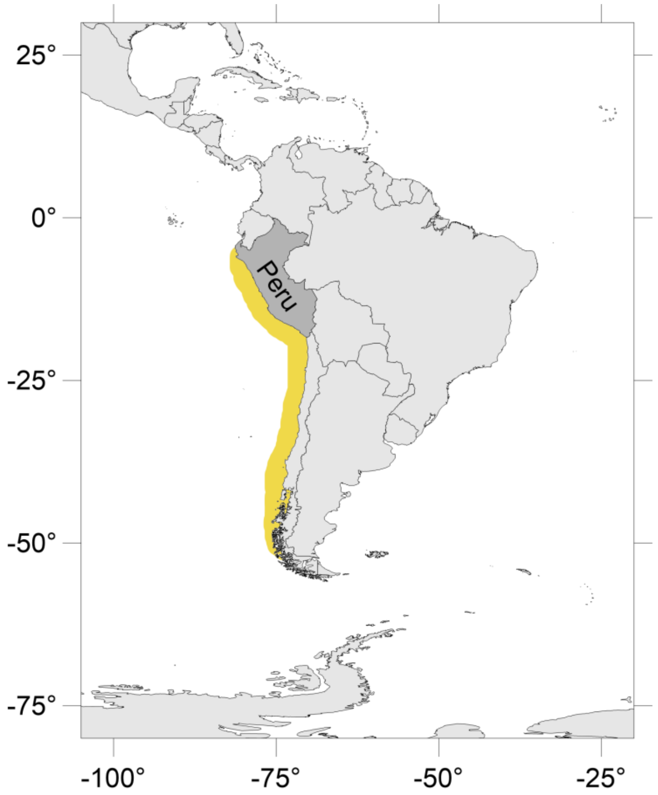

Spatial availability of unoccupied habitat for most critical life stage – ability to shift distributional range.	Approximately 27° of latitude may be available to the south of Peru.
Phenology	
Environmental variable as a phenological cue for spawning or breeding – cues include salinity, temperature, currents, and freshwater flows.	Not available.
Environmental variable as a phenological cue for settlement or metamorphosis	Not available.
Temporal mismatches of life-cycle events – duration of spawning, breeding or moulting season.	Appears to have different spawning peaks depending on the area; the reproductive events in Huacho occurred in spring and winter, in Callao in autumn, in Pisco in winter and summer, and in Ilo in winter (Mejía *et al.* 1970; González-Ynope 2001); duration of spawning is thus assumed to last > 4 months.
Migration (seasonal and spawning)	Not available.
Exposure	
Response to environmental variability	During El Niño events, the capture of silverside is reduced; for instance, the incursion of warm waters (e.g., during El Niño events) to the Peruvian coast causes the decrease of phytoplankton blooms and of primary production. As a consequence, the species move to other areas due to the lack of food, which may in part explain the landing fluctuations of coastal species such as silverside in 1996–1999 (González-Ynope 2001).
References	

Arrieta, S.B., Goicochea, C.E., & Mostacero, J.A. Edad y crecimiento del pejerrey *Odontesthes regia regia* (Humboldt) en el mar peruano. 2002. *Inf. Inst. Mar Perú* **37**, 75–77 (2010).

Chirichigno, N., & Cornejo, R.M. Catálogo comentado de los peces marinos del Perú. *Inst. Mar Perú*. Callao, Perú (2001).

Chirinos, A., & Chuman, E. Notas sobre el desarrollo de huevos y larvas del pejerrey *Odontesthes* (*Austromenidia*) *regia regia* (Humboldt). *Bol. Inst. Mar Perú* **1**, pp 31 (1964).

Chura-Cruz, R., Cubillos, L.A., Tam, J., Segura, M., & Villanueva, C. Relación entre el nivel del lago y la precipitación sobre los desembarques del pejerrey *Odontesthes bonariensis* (Valenciennes, 1835) en el sector peruano del lago Titicaca entre 1981 y 2010. *Ecol. Apl.* **12**, 19–28 (2013).

González-Ynope, A. Contribución al conocimiento pesquero y biológico de cinco peces costeros de importancia comercial en el Perú: Cabinza, lisa, lorna, machete y pejerrey. Periodo 1996–2000. *Inf. Prog. Inst. Mar Perú* **195**, pp 46 (2001).

IMARPE. Programa de apoyo a la pesca artesanal, la acuicultura y el manejo sostenible del ambiente (2007–2010) Propesca. Informe de actividades 2008. Puno, Peru. pp 28 (2009).

IMARPE. Anuario Científico Tecnológico 15. PRODUCE-IMARPE. pp 255 (2015).

IMARPE-PELT. Crucero de evaluación de recursos pesqueros y condiciones limnológicas del lago Titicaca – CR. 1407. Informe técnico. *IMARPE-PELT*. pp 12 (2015).

INIDEP. Pejerrey escardón o baboso. Available at: https://www.inidep.edu.ar/especies/48-pejerrey-escardon-o-baboso.html (2021).

Mejía, J., Samamé, M., & Pastor, A. Información básica de los principales peces de consumo. *Instituto del Mar, Serie de Informes Especiales IM–62*. pp 29 (1970).

Plaza, G., Espejo, V., Almanza, V., & Claramunt, G. Female reproductive biology of the silverside *Odontesthes regia*. *Fish. Res.* **111**, 31–39 (2011).

Reis, R., & Lima, F. *Odontesthes regia*. The IUCN Red List of Threatened Species 2009: e.T167710A6371187. Available at: http://dx.doi.org/10.2305/IUCN.UK.2009-2.RLTS.T167710A6371187.en (2009).

1.28 Yellowfin tuna - *Thunnus albacares*

Sensitivity	
Abundance	
Fecundity – egg production (total fecundity)	In Hawaiian waters its fecundity is 2,370,000–8,590,000 eggs, which annually can reach up to 60,000,000 eggs (Joseph 1963; FAO 2021).
Recruitment period – successful recruitment event that sustains the abundance of the fishery	Recruitment events are likely consistent every 1–2 years, however these also may be variable.
Average age at maturity	Reaches sexual maturity at 2.5–3 years (Joseph 1963; FAO 2021).
Generalist vs. specialist – food and habitat	In general, tunas are opportunistic and do not depend on a particular type of prey; the larvae feed on zooplankton. In

	particular, the yellow fin tuna feeds on mesopelagic fish but a type of prey may be dominant depending on the area and season (FAO 2021). Mesopelagic species that is essentially confined to the upper 100 m of the water column in areas with marked oxyclines (Collette & Nauen 1983; FAO 2021).
Biomass	The yellow fin tuna had a maximum biomass in 2001, which declined in 2002, and is considered fully exploited (Shotton 2005). According to the IUCN Red List this species is in the category NEAR THREATENED (NT) (Collette *et al.* 2011), i.e., it is not currently vulnerable or endangered but is likely to be in the near future.
Distribution	
Capacity for larval dispersal or larval duration – hatching to settlement (benthic species), hatching to yolk sac re-adsorption (pelagic species).	The passive transport of *T. albacares* probably occurs for 8–10 days, while larvae still have the yolk sack (Wexler *et al.* 2007).
Capacity for adult/juvenile movement – lifetime range post-larval stage.	Tunas move constantly to allow water through their gills and perform long seasonal migrations in search of food and to reproduce (FAO 2021). Its capable of active movementes of around 633 miles (1,019 km) monthly in the Eastern Pacific (Fonteneau & Hallier 2015).
Physiological tolerance – latitudinal coverage of adult species as a proxy of environmental tolerance.	Cosmopolitan species that occurs in the Eastern Pacific from Punta Concepcion, U.S.A. to the south of Valdivia, Chile (40°S) (IMARPE); approximately 73° of latitudinal coverage. 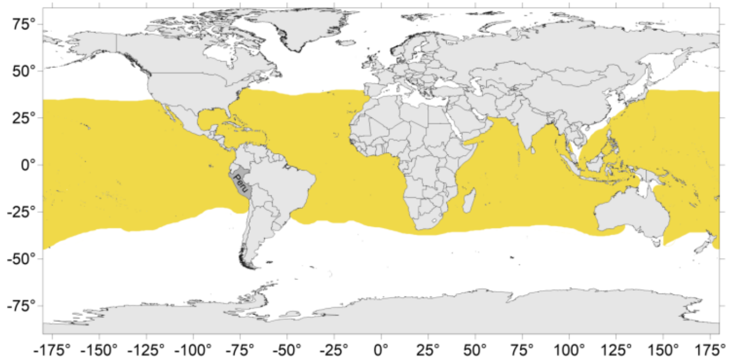
Spatial availability of unoccupied habitat for most critical life stage – ability to shift distributional range.	Approximately 11° of latitude may be available to the south of Peru; approximately 39° of latitude may be available to the north of Peru.
Phenology	

Environmental variable as a phenological cue for spawning or breeding – cues include salinity, temperature, currents, and freshwater flows.	The temperature window for spawning is narrower and higher than 24°C, and larvae occur in warm surface waters (FAO 2021).
Environmental variable as a phenological cue for settlement or metamorphosis	Not available.
Temporal mismatches of life-cycle events – duration of spawning, breeding or moulting season.	Spawns throughout the year but has higher spawning peaks in summer (Joseph 1963; FAO 2021); duration of spawning is thus assumed to last > 4 months.
Migration (seasonal and spawning)	Not available.
Exposure	
Response to environmental variability	This species occurs approximately between 18°C and 31°C (Collette & Nauen 1983). The temperature window for spawning is narrower and higher than 24°C, and larvae occur in warm surface waters (FAO 2021). During El Niño 1997–1998, tuna underwent changes in distribution to the south, from Ecuador to northern Peru, and reproductive events and landings increased in Peru (Ñiquen & Bouchon 2004).<br><br>Ocean acidification causes damage to the kidneys, liver, pancreas, eyes and muscles in larvae; and thus it has negative effects on larval growth and survival (Frommel *et al.* 2016).<br><br>Concentrations of dissolved oxygen <2 mL/L below the thermocline exclude the presence of this species (Collette & Nauen 1983). The habitat of this species may be reduced due to the increase in temperature and decrease in oxygen concentration likely associated with climate change.

References

Collette, B. *et al. Thunnus albacares*. The IUCN Red List of Threatened Species 2011: e.T21857A9327139. Available at: http://dx.doi.org/10.2305/IUCN.UK.2011-2.RLTS.T21857A9327139.en (2011).

Collette, B.B., & Nauen, C.E. FAO Species Catalogue. Vol. 2. Scombrids of the world. An annotated and illustrated catalogue of Tunas, Mackerels, Bonitos and related species known to date. FAO Fish. Synop. 125: 137 p. Available at: http://www.fao.org/fishery/species/2497/en (1983).

FAO. Tuna. Available at: http://www.fao.org/fishery/topic/16082/en (2021).

Fonteneau, A., & Hallier, J.P. Fifty years of dart tag recoveries for tropical tuna: A global comparison of results for the western Pacific, eastern Pacific, Atlantic, and Indian Oceans. *Fish. Res.* **163**, 7–22 (2015).

Frommel, A.Y. *et al.* Ocean acidification has lethal and sub-lethal effects on larval development of yellowfin tuna, *Thunnus albacares*. *J. Exp. Mar. Biol. Ecol.* **482**, 18–24 (2016).

Joseph, J. Fecundity of yellowfin tuna (*Thunnus albacares*) and skipjack (*Katsuwonus pelamis*) from the Eastern Pacific Ocean. *IATTC* **7**, 257–292 (1963).

Ñiquen, M., Bouchon, M. Impact of El Niño events on pelagic fisheries in Peruvian waters. *Deep Sea Res. Part II Top. Stud. Oceanogr.* **51**, 563–574 (2004).

Shotton, R. Examen de la situación de los recursos pesqueros marinos mundiales. FAO. Available at: http://www.fao.org/publications/card/en/c/d69fadd1-6426-552f-bda6-b715a383d7da/ (2005).

Wexler, J.B., Chow, S., Wakabayashi, T., Nohara, K., Margulies, D. Temporal variation in growth of yellowfin tuna (*Thunnus albacares*) larvae in the Panama Bight, 1990−97. *Fish. Bull.* **105**, 1–18 (2007).

Methods S2. Climate exposure factors

The change in mean climate conditions based on a standard deviate of the modeled future relative to the past was mapped with the NOAA Climate Change Web Portal for the area 6°S, 98°W, 20°S, 68°W, using the Standard Anom (average historical) statistic following Hare *et al.* (2016):

$$Z = \frac{\bar{X}_f - \bar{X}_p}{\sigma_p}$$

where $\bar{X}_f \bar{X}_f$ is the mean of the future (2006–2055), $\bar{X}_p \bar{X}_p$ is the mean of the past (1956–2005), and $\sigma_p \sigma_p$ is the standard deviation of the past.

The magnitude of change was 'high' if the future mean climate was > 2 standard deviations different than the historical climate. The magnitude of change was 'medium' if the future mean climate was > 1 standard deviation but ≤ 2 standard deviations different than the historical climate. The magnitude of change was 'low' if the future mean climate was ≤ 1 standard deviation different than the historical climate. The probability for each level of change was estimated based on the theoretical distribution of the change of the climate exposure factor.

Oceanic and neritic areas were examined separate. Changes in inland precipitation and air temperature were examined to assess the exposure of species located near the shore and in inland bodies of water. Negative and positive changes in climate factors were taken into account.

Sea surface temperature

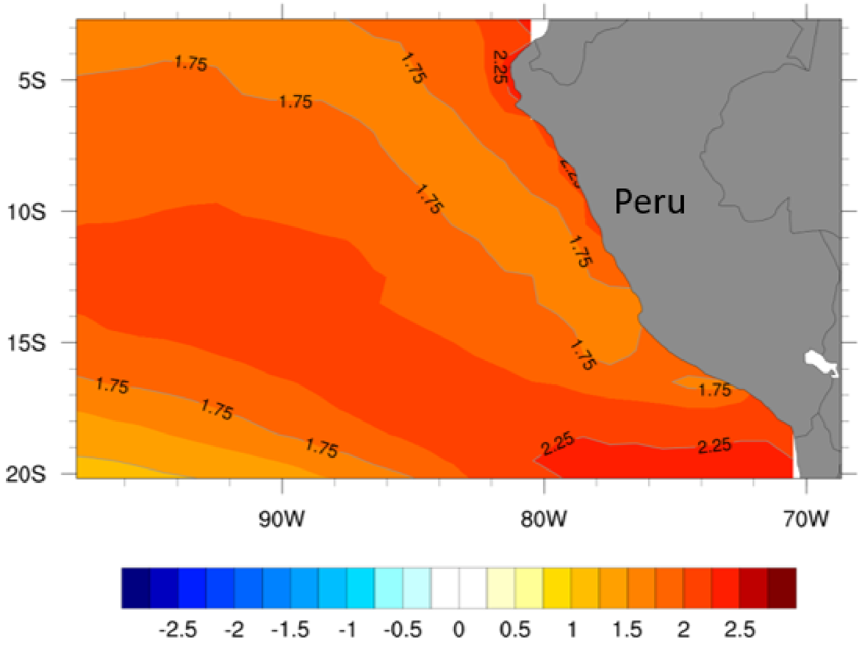

Difference in the mean sea surface temperature in the future (2006–2055) compared with the past (1956–2005) divided by the standard deviation of the past (Z), based on the “business as usual” Representative Concentration Pathway 8.5 (RCP8.5)

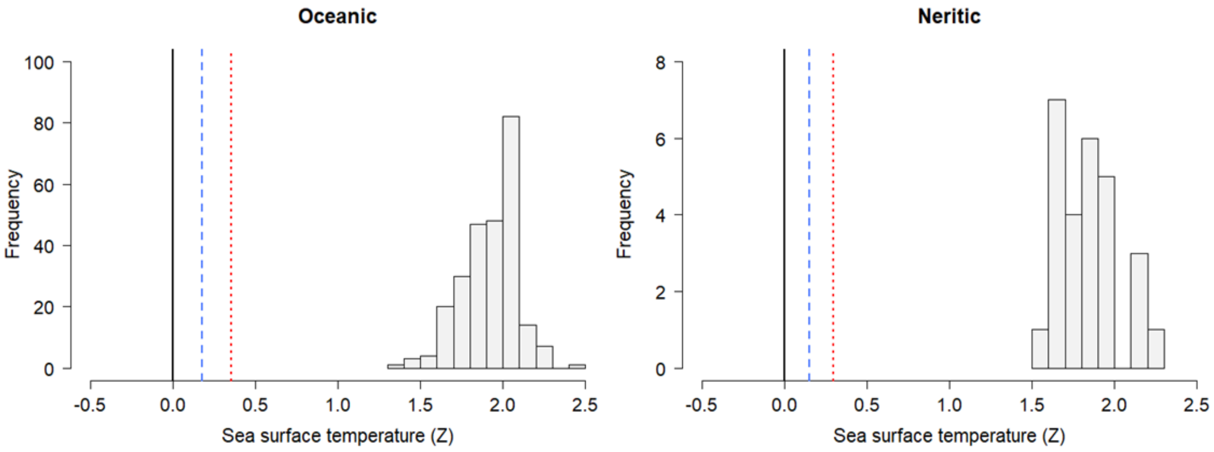

Frequency distribution of the change in mean oceanic and neritic sea surface temperature in the future (2006–2055) compared with the past (1956–2005) (Z). The solid black line indicates no change. The blue dashed line indicates one standard deviation and the red dotted line indicates two standard deviations, representing the low, medium and high magnitude of change thresholds, respectively

Categories of magnitude of change of mean sea surface temperature (SST) and corresponding approximate probabilities

Magnitude of change (SST)	Thresholds	Approximate probabilities
Oceanic		
High	0.3509 < Z	99.9%
Medium	0.1754 < Z ≤ 0.3509	< 0.1%
Low	Z ≤ 0.1754	< 0.1%
Neritic		
High	0.3053 < Z	99.9%
Medium	0.1526 < Z ≤ 0.3053	< 0.1%
Low	Z ≤ 0.1526	< 0.1%

Z = Difference of the mean SST in the future (2006–2055) compared with the past (1956–2005)

Sea surface salinity

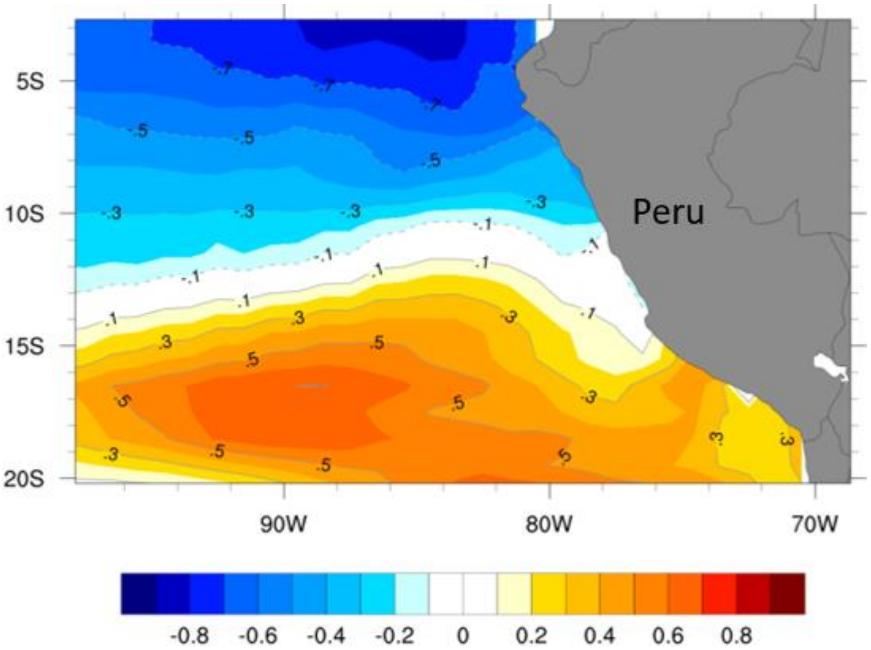

Difference in the mean sea surface salinity in the future (2006–2055) compared with the past (1956–2005) divided by the standard deviation of the past (Z), based on the "business as usual" Representative Concentration Pathway 8.5 (RCP8.5)

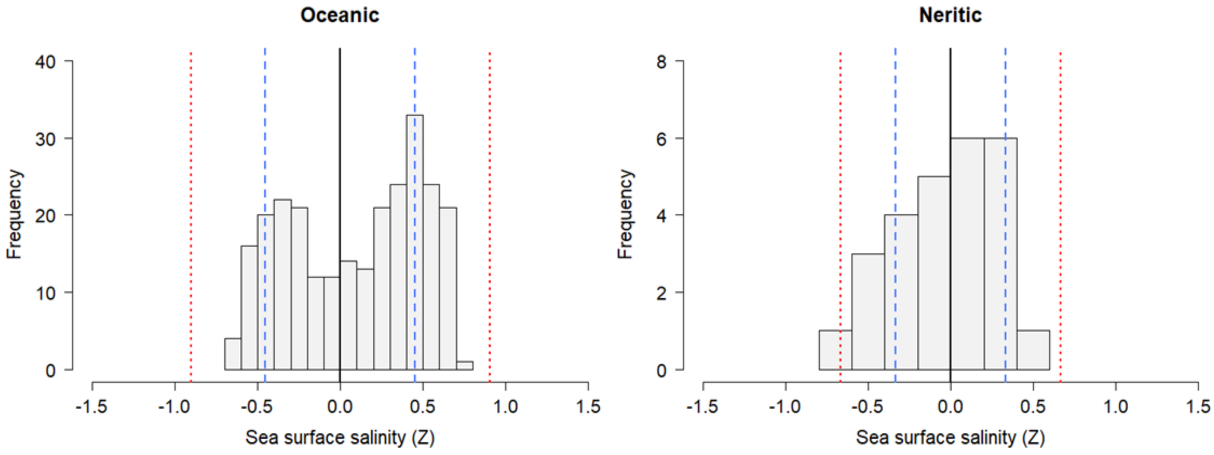

Frequency distribution of the change in mean oceanic and neritic sea surface salinity in the future (2006–2055) compared with the past (1956–2005) (Z). The solid black line indicates no change. The blue dashed line indicates one standard deviation and the red dotted line indicates two standard deviations, representing the low, medium and high magnitude of change thresholds, respectively

Categories of magnitude of change of mean sea surface salinity (SSS) and corresponding approximate probabilities

**Magnitude of change (SSS)**	**Thresholds**	**Approximate probabilities**
Oceanic (negative)		
High	Z < –0.9070	2.4%
Medium	–0.9070 ≤ Z < –0. 4535	8.4%
Low	–0.4535 ≤ Z < 0.0000	24.7%
Oceanic (positive)		
High	0.9070 < Z	0.0%
Medium	0.4535 < Z ≤ 0.9070	20.3%
Low	0.0000 < Z ≤ 0.4535	44.1%
Neritic (negative)		
High	Z < –0.6657	3.3%
Medium	–0.6657 ≤ Z < –0.3328	15.9%
Low	–0.3328 ≤ Z < 0.0000	35.0%
Neritic (positive)		
High	0.6657 < Z	0.2%
Medium	0.3328 < Z ≤ 0.6657	11.7%
Low	0.0000 < Z ≤ 0.3328	33.9%

Z = Difference of the mean SSS in the future (2006–2055) compared with the past (1956–2005)

pH

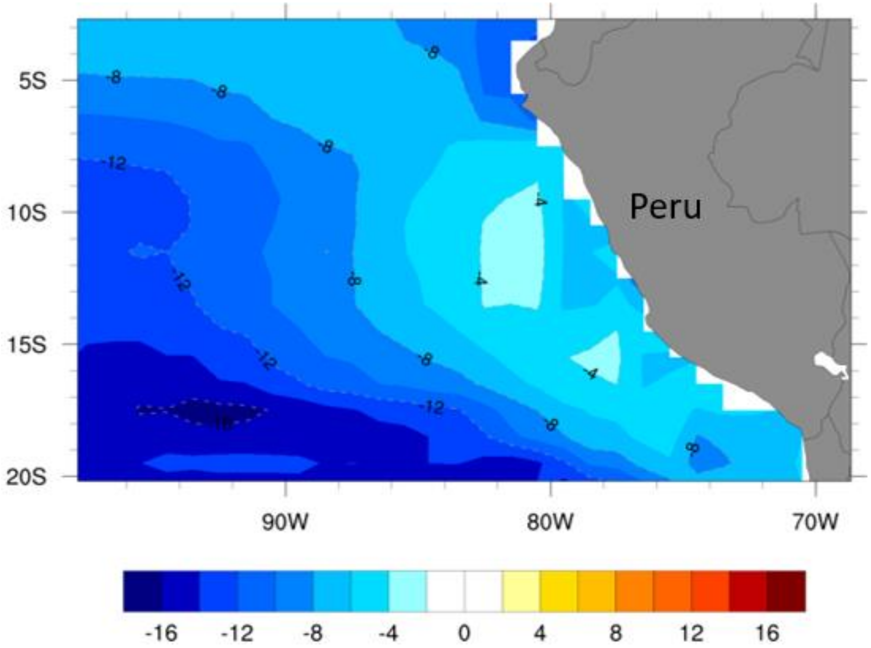

Difference in the mean pH in the future (2006–2055) compared with the past (1956–2005) divided by the standard deviation of the past (Z), based on the “business as usual” Representative Concentration Pathway 8.5 (RCP8.5)

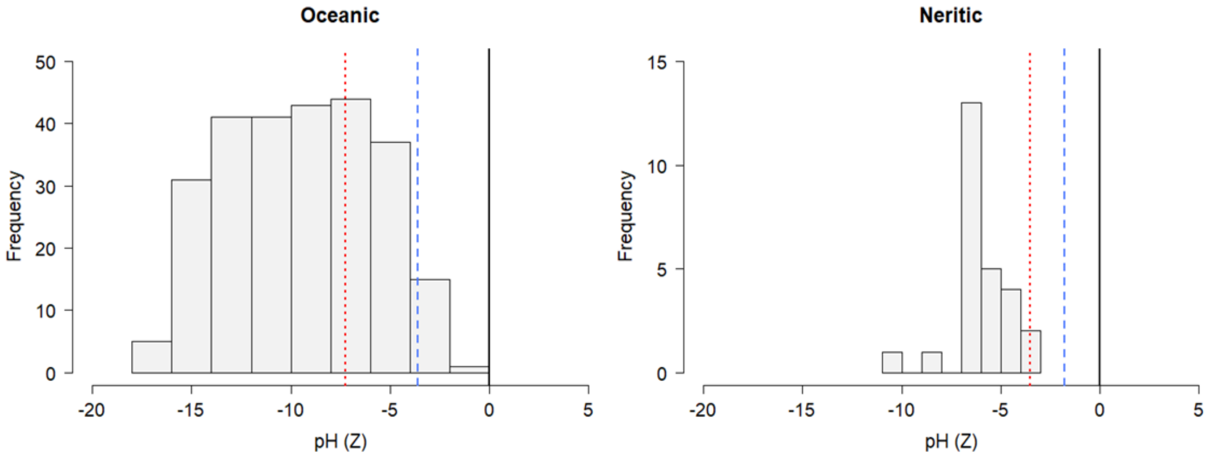

Frequency distribution of the change in mean oceanic and neritic pH in the future (2006–2055) compared with the past (1956–2005) (Z). The solid black line indicates no change. The blue dashed line indicates one standard deviation and the red dotted line indicates two standard deviations, representing the low, medium and high magnitude of change thresholds, respectively

Categories of magnitude of change of mean pH and corresponding approximate probabilities

Magnitude of change (pH)	Thresholds	Approximate probabilities
Oceanic		
High	–7.2471 < Z	72.6%
Medium	–3.6235 < Z ≤ –7. 2471	22.1%
Low	Z ≤ –3.6235	5.3%
Neritic		
High	–3.5201 < Z	98.2%
Medium	–1.7600 < Z ≤ –3.5201	1.8%
Low	Z ≤ –1.7600	0.0%

Z = Difference of the mean pH in the future (2006–2055) compared with the past (1956–2005)

Chlorophyll

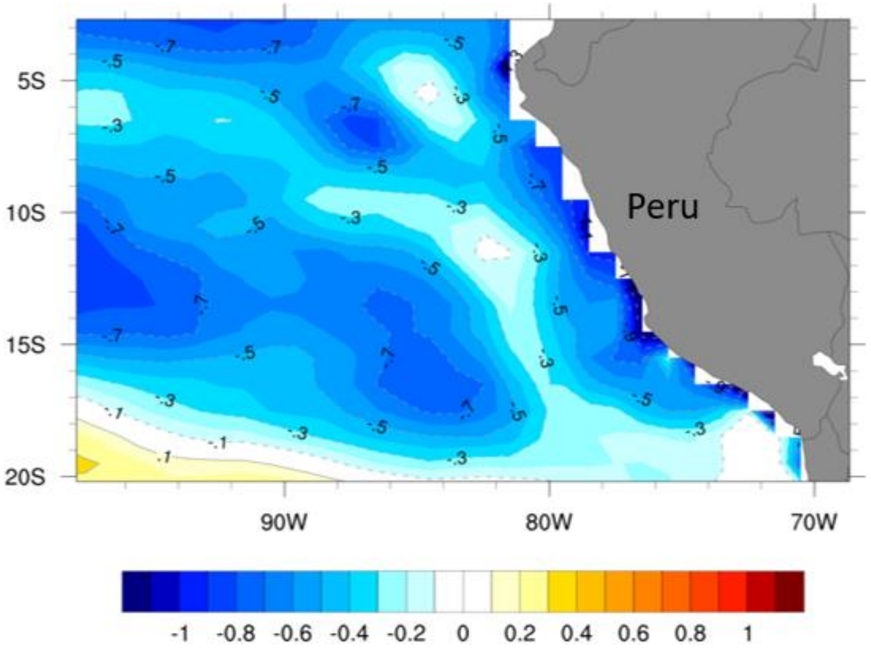

Difference in the mean chlorophyll mass in the future (2006–2055) compared with the past (1956–2005) divided by the standard deviation of the past (Z), based on the "business as usual" Representative Concentration Pathway 8.5 (RCP8.5)

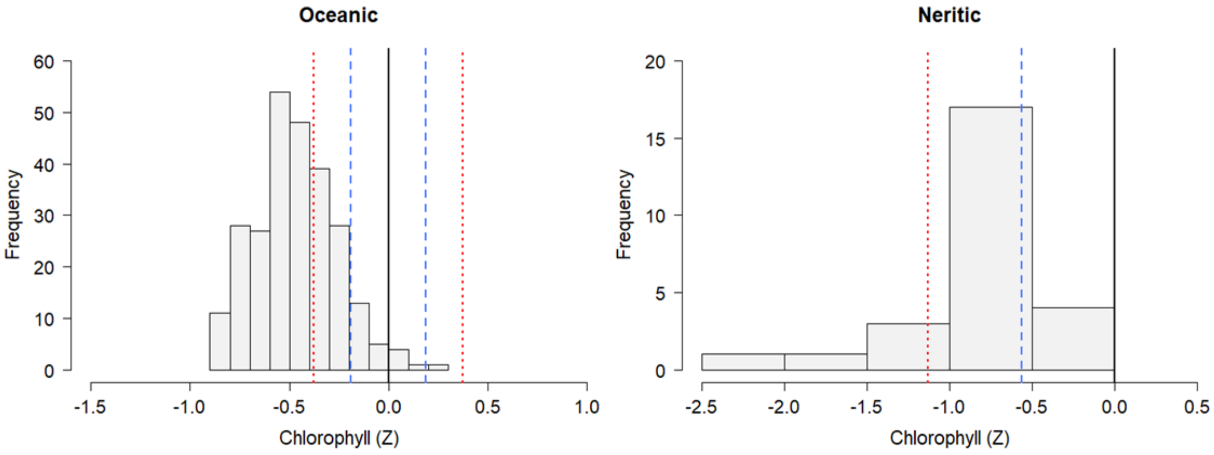

Frequency distribution of the negative and positive change in mean oceanic and neritic chlorophyll in the future (2006–2055) compared with the past (1956–2005) (Z). The solid black line indicates no change. The blue dashed line indicates one standard deviation and the red dotted line indicates two standard deviations, representing the low, medium and high magnitude of change thresholds, respectively

Categories of magnitude of change of mean chlorophyll (Chl) and corresponding approximate probabilities

**Magnitude of change (Chl)**	**Thresholds**	**Approximate probabilities**
Oceanic (negative)		
High	Z < –0.3759	69.0%
Medium	–0.3759 ≤ Z < –0.1879	21.1%
Low	–0.1879 ≤ Z < 0.0000	7.7%
Oceanic (positive)		
High	0.4942 < Z	<0.1%
Medium	0.2471 < Z ≤ 0.4942	0.3%
Low	0.0000 < Z ≤ 0.2471	1.9%
Neritic (negative)		
High	Z < –1.1297	26.6%
Medium	–1.1297 ≤ Z < –0.5648	38.6%
Low	–0.5648 ≤ Z <	34.8%

Z = Difference of the mean Chl in the future (2006–2055) compared with the past (1956–2005)

Primary productivity

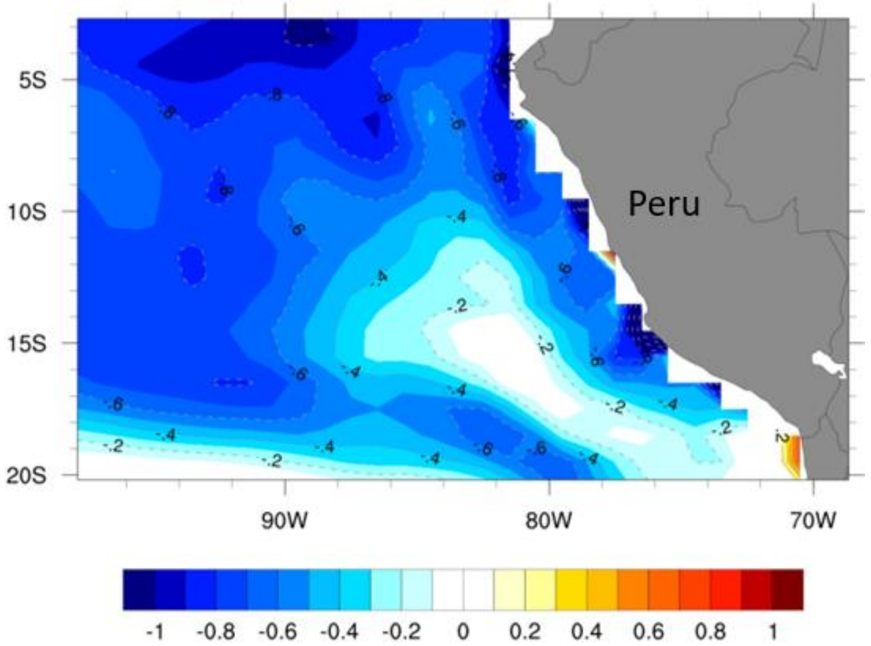

Difference in the mean primary productivity in the future (2006–2055) compared with the past (1956–2005) divided by the standard deviation of the past (Z), based on the "business as usual" Representative Concentration Pathway 8.5 (RCP8.5)

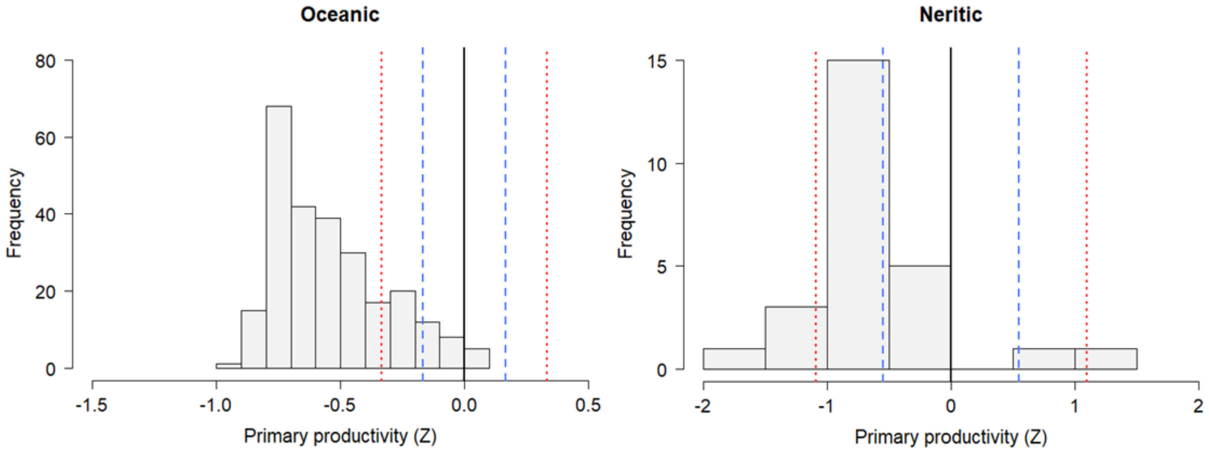

Frequency distribution of the change in mean oceanic and neritic primary productivity in the future (2006–2055) compared with the past (1956–2005) (Z). The solid black line indicates no change. The blue dashed line indicates one standard deviation and the red dotted line indicates two standard deviations, representing the low, medium and high magnitude of change thresholds, respectively

Categories of magnitude of change of mean primary productivity (PP) and corresponding approximate probabilities

**Magnitude of change (PP)**	**Thresholds**	**Approximate probabilities**
Oceanic (negative)		
High	Z < –0.3335	84.2%
Medium	–0.3335 ≤ Z < –0.1667	8.3%
Low	–0.1667 ≤ Z < 0.0000	3.8%
Oceanic (positive)		
High	0.3335 < Z	1.0%
Medium	0.1667 < Z ≤ 0.3335	0.9%
Low	0.0000 < Z ≤ 0.1667	1.8%
Neritic (negative)		
High	Z < –1.0939	19.3%
Medium	–1.0939 ≤ Z < –0.5469	35.3%
Low	–0.5469 ≤ Z < 0.0000	24.8%
Neritic (positive)		
High	1.0939 < Z	3.6%
Medium	0.5469 < Z ≤ 1.0939	5.1%
Low	0.0000 < Z ≤ 0.5469	11.9%

Z = Difference of the mean PP in the future (2006–2055) compared with the past (1956–2005)

Precipitation

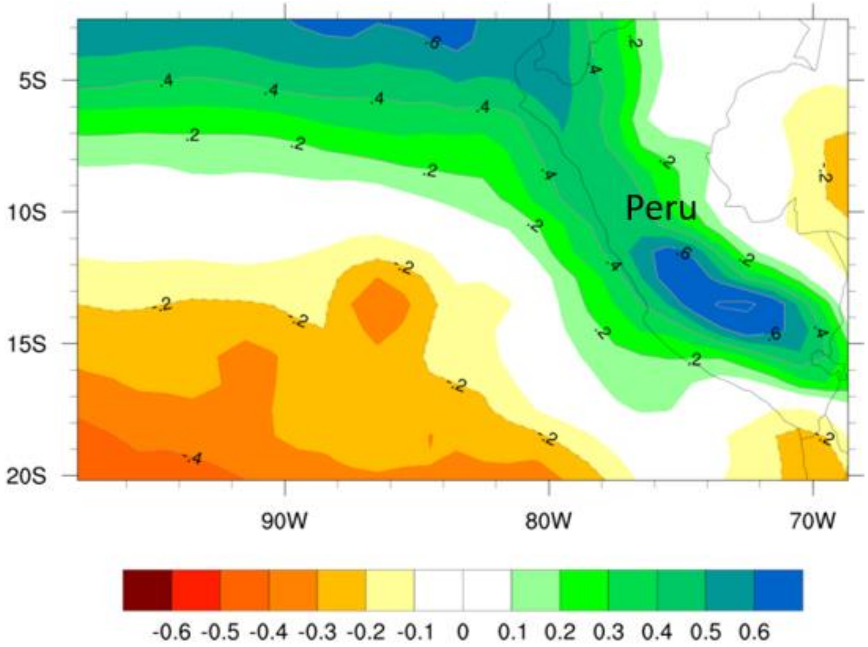

Difference in the mean precipitation in the future (2006–2055) compared with the past (1956–2005) divided by the standard deviation of the past (Z), based on the "business as usual" Representative Concentration Pathway 8.5 (RCP8.5)

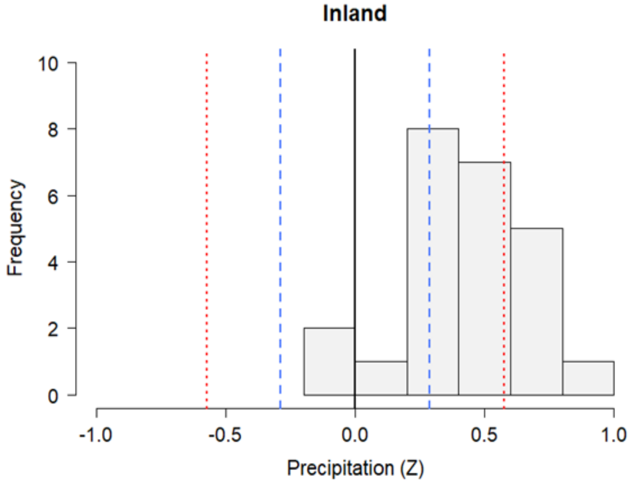

Frequency distribution of the change in mean inland precipitation in the future (2006–2055) compared with the past (1956–2005) (Z). The solid black line indicates no change. The blue dashed line indicates one standard deviation and the red dotted line indicates two standard deviations, representing the low, medium and high magnitude of change thresholds, respectively

Categories of magnitude of change of mean inland precipitation and corresponding approximate probabilities

Magnitude of change (precipitation)	Thresholds	Approximate probabilities
Inland (negative)		
High	Z < –0.5746	<0.1%
Medium	–0.5746 ≤ Z < –0.2873	0.9%
Low	–0.2873 ≤ Z < 0.0000	6.7%
Inland (positive)		
High	0.5746 < Z	29.6%
Medium	0.2873 < Z ≤ 0.5746	39.3%
Low	0.0000 < Z ≤ 0.2873	23.4%

Z = Difference of the mean precipitation in the future (2006–2055) compared with the past (1956–2005)

Air temperature

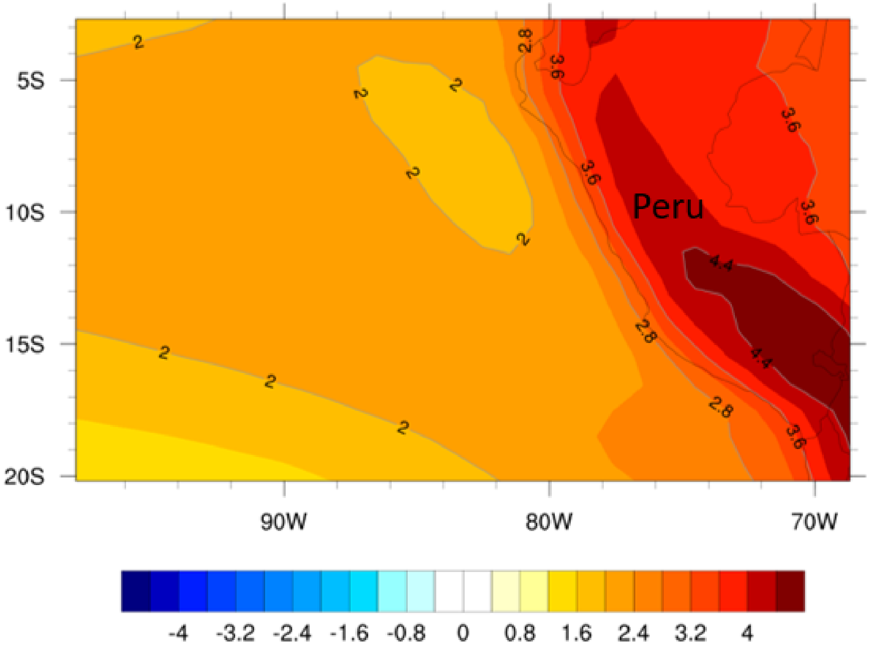

Difference in the mean coastal and inland air temperature in the future (2006–2055) compared with the past (1956–2005) divided by the standard deviation of the past (Z), based on the "business as usual" Representative Concentration Pathway 8.5 (RCP8.5)

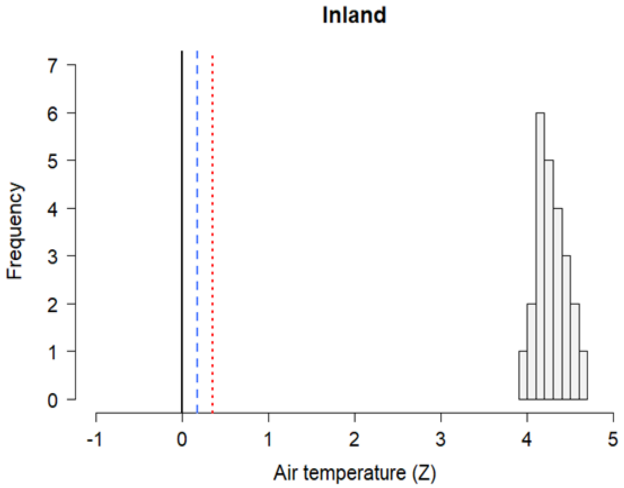

Frequency distribution of the change in mean onshore and inland air temperature in the future (2006–2055) compared with the past (1956–2005) (Z). The solid black line indicates no change. The blue dashed line indicates one standard deviation and the red dotted line indicates two standard deviations, representing the low, medium and high magnitude of change thresholds, respectively

Categories of magnitude of change of mean coastal and inland air temperature (AT) and corresponding approximate probabilities

**Magnitude of change (AT)**	**Thresholds**	**Approximate probabilities**
Inland		
High	0.3492 < Z	99.9%
Medium	0.1746 < Z ≤ 0.3492	< 0.1%
Low	Z ≤ 0.1746	< 0.1%

Z = Difference of the mean AT in the future (2006–2055) compared with the past (1956–2005)

Sea bottom temperature

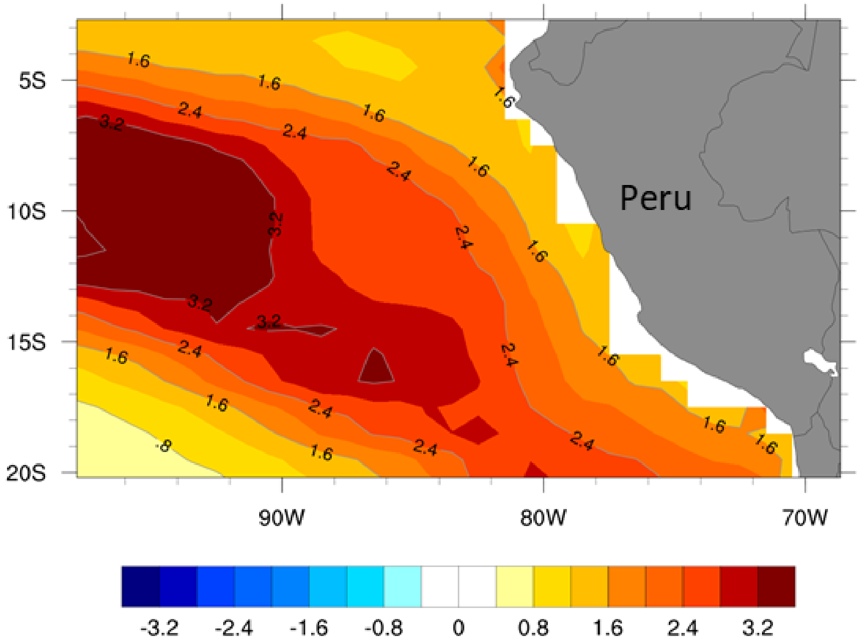

Difference in the mean sea bottom temperature in the future (2006–2055) compared with the past (1956–2005) divided by the standard deviation of the past (Z), based on the "business as usual" Representative Concentration Pathway 8.5 (RCP8.5)

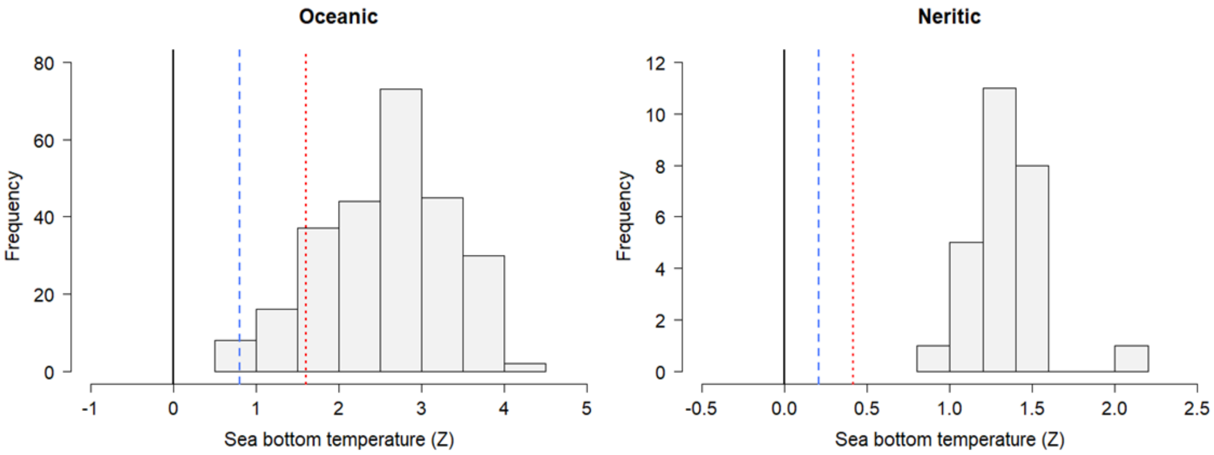

Frequency distribution of the change in mean oceanic and neritic sea bottom temperature in the future (2006–2055) compared with the past (1956–2005) (Z). The solid black line indicates no change. The blue dashed line indicates one standard deviation and the red dotted line indicates two standard deviations, representing the low, medium and high magnitude of change thresholds, respectively

Categories of magnitude of change of mean sea bottom temperature (SBT) and corresponding approximate probabilities

**Magnitude of change (SBT)**	**Thresholds**	**Approximate probabilities**
Oceanic		
High	1.5989 < Z	88.6%
Medium	0.7994 < Z ≤ 1.5989	9.8%
Low	Z ≤ 0.7994	1.6%
Neritic		
High	0.4131 < Z	99.9%
Medium	0.2065 < Z ≤ 0.4131	< 0.1%
Low	Z ≤ 0.2065	< 0.1%

Z = Difference of the mean SBT in the future (2006–2055) compared with the past (1956–2005)

Sea bottom salinity

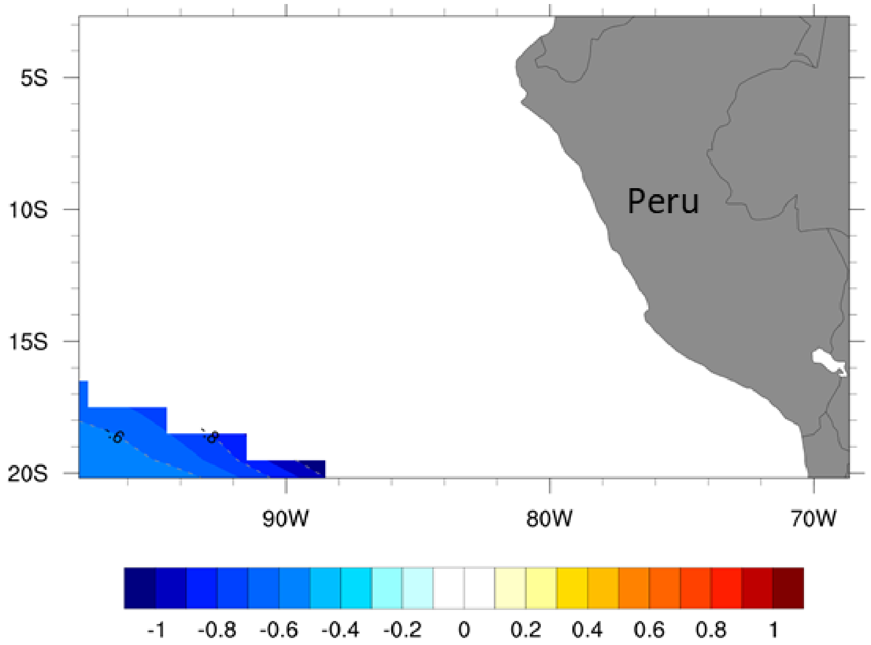

Difference in the mean sea bottom salinity in the future (2006–2055) compared with the past (1956–2005) divided by the standard deviation of the past (Z), based on the “business as usual” Representative Concentration Pathway 8.5 (RCP8.5)

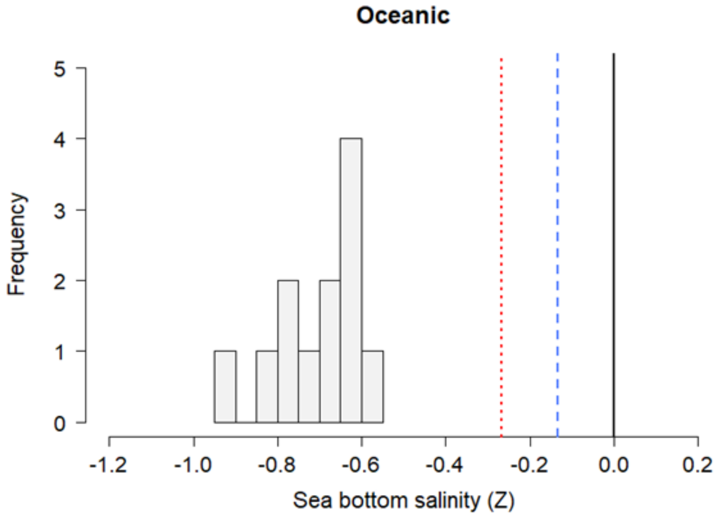

Frequency distribution of the change in mean oceanic sea bottom salinity (absolute values) in the future (2006–2055) compared with the past (1956–2005) (Z). The solid black line indicates no change. The blue dashed line indicates one standard deviation and the red dotted line indicates two standard deviations, representing the low, medium and high magnitude of change thresholds, respectively

Categories of magnitude of change of mean sea bottom salinity (SBS) and corresponding approximate probabilities

Magnitude of change (SBS)	Thresholds	Approximate probabilities
Oceanic		
High	–0.2673 < Z	99.9%
Medium	–0.1336 < Z ≤ –0.2673	< 0.1%
Low	Z ≤ –0.1336	< 0.1%

Z = Difference of the mean SBS in the future (2006–2055) compared with the past (1956–2005)

References

NOAA's Ocean Climate Change Web Portal https://psl.noaa.gov/ipcc/ocn/ (2014).

Hare, J.A. *et al*. A vulnerability assessment of fish and invertebrates to climate change on the Northeast U.S. Continental Shelf. *PLoS ONE* **11**, e0146756. https://doi.org/10.1371/journal.pone.0146756 (2016).

Methods S3. Climate exposure factor: Sea level rise

The Intergovernmental Panel on Climate Change Fifth Assessment Report (AR5) estimates that the rate of global mean sea level rise for the period 2081–2100 will be 2.0–15.7 mm/year, reaching a sea level rise between 0.26 to 0.82 m across the different Representative Concentration Pathways (RCPs). The "business as usual" RCP8.5 scenario estimates a global mean sea level rise of 0.22 to 0.38 m during 2046–2065; a rate of 8–16 mm/year during 2081–2100 will result in a sea level rise of 0.52 to 0.98 m by the year 2100 (Church *et al.* 2013). Off Peru, the expected mean trend in sea level rise is of approximately 1.2–2.8 mm/year for 2010–2040, and 1.2–3.2 mm/year for the period 2040–2070 (UN-ECLAC 2015).

Sea level rise will have considerable impacts on the structure of the shoreline and on coastal ecosystems due to intrusion of salt water, accretion of sediments, coastal erosion, increase in water depths, change in tidal variation and water movement (Short & Neckles 1999; De Silva & Soto 2009). Salt water intrusion may represent a threat for early stages of commercial or ecologically important species that use mangroves as nursery grounds. In this sense, salt water intrusion associated with sea level rise will impose ecological and habitat changes with consequences on fisheries production in deltaic areas and brackish habitats (De Silva & Soto 2009). Oceanic water intrusion into fresh or brackish water areas also will affect estuarine plant distribution because of the effect of salinity change on seed germination, propagule formation, photosynthesis, growth and biomass. Whereas increased water depth, water motion and tidal circulation reduces the amount of light that reaches plants, negatively affecting photosynthetic rates (Short & Neckles 1999), with consequences on the structure of plant communities and animal species that rely on those habitats.

Marsh ecosystems appear to be stable at local sea level rise of 2–3 mm/year; however, projected sea level rise of ~5 mm/year can result in a shift from marshes to unvegetated subtidal environments (Kirwan *et al.* 2010; Morris *et al.* 2016). At first glance, coastal wetlands of Peru appear to be able to resist the impacts

of sea level rise rates estimated for the region (mean range = 1.2–2.8 mm/year for 2010–2040; 1.2–3.2 mm/year for 2040–2070). However, sediment accretion and tide breadth also influence the capacity of wetlands to counteract the effects of sea level rise. For instance, critical rates of sea level rise for marshes can be of only a few millimeters per year at low suspended sediment concentrations (~1–10 mg/L); whereas adaptation can occur at high suspended sediment concentrations (30–100 mg/L) (Kirwan *et al.* 2010). Overall, marshes with high tidal ranges and high suspended sediment concentrations are more resilient to sea level rise compared with marshes with low tidal ranges and low suspended sediment concentrations (Kirwan *et al.* 2010). Critical sea level rise for Peruvian coastal wetlands should be taken with caution given that, to our knowledge, there are no estimations on accretion rate for such ecosystems in the region.

Scoring

Studies suggest that wetlands may adapt at local sea level rise of 2–3 mm/year but would be highly affected at sea level rise >5 mm/year, turning into unvegetated subtidal environments (Kirwan *et al.* 2010; Morris *et al.* 2016). Assign your tallies across all three bins based on the dependency of the species on habitats that are expected to be affected by sea level rise. Take in consideration the effect of regional differences in sea level rise and fixed shoreline structures built to minimize the impact of shore erosion and floods, and that can stop coastal wetland communities from migrating inland (Nicholls 2004; Nicholls *et al.* 2007). The three bin scores (low, medium, high) are defined as:

Category	Score	Criteria
High	3	The species depends on wetland or estuary habitat at any given life history stage, and the regional sea level rise is >5 mm year^{-1} by 2050
Medium	2	The species depends on wetland or estuary habitat at any given life history stage, and the regional sea level rise is 2–3 mm year^{-1} by 2050
Low	1	The species does not depend on wetland or estuary habitat at any life history stage

References

Church, J.A. *et al.* Sea Level Change. In: Climate Change 2013: The Physical Science Basis. Contribution of Working Group I to the Fifth Assessment Report of the Intergovernmental Panel on Climate Change (eds. Stocker, T.F. *et al.*) 1139–1216 (Cambridge University Press, 2013).

De Silva, S.S., & Soto, D. Climate change and aquaculture: potential impacts, adaptation and mitigation. In: Climate change implications for fisheries and aquaculture: overview of current scientific knowledge. FAO Fisheries and Aquaculture Technical Paper **530 (**eds. Cochrane, K., De Young, C., Soto, D., & Bahri, T.) 151–212 (FAO, 2009).

Kirwan, M.L., *et al.* Limits on the adaptability of coastal marshes to rising sea level. *Geophys. Res. Lett.* **37**, L23401. https://doi.org/10.1029/2010GL045489 (2010).

Morris, J.T. *et al.* Contributions of organic and inorganic matter to sediment volume and accretion in tidal wetlands at steady state. *Earths Future* **4**, 110–121. https://doi.org/10.1002/2015EF000334 (2016).

Nicholls, R.J. Coastal flooding and wetland loss in the 21st century: changes under the SRES climate and socio-economic scenarios. *Glob. Environ. Change* **14**, 69–86. https://doi.org/10.1016/j.gloenvcha.2003.10.007 (2004).

Nicholls, R.J. *et al.* Coastal systems and low-lying areas. In: Climate Change 2007: Impacts, Adaptation and Vulnerability. Contribution of Working Group II to the Fourth Assessment Report of the Intergovernmental Panel on Climate Change (eds. Parry, M.L., Canziani, O.F., Palutikof, J.P., van der Linden, P.J., & Hanson, C.E.) 315–356, https://www.ipcc.ch/publications_and_data/ar4/wg2/en/ch6.html (Cambridge University Press, 2007).

Short, F.T., & Neckles, H.A. The effects of global climate change on seagrasses. *Aquat. Bot.* **63**, 169–196. https://doi.org/10.1016/S0304-3770(98)00117-X (1999).

ECLAC (2015) Climate variability, dynamics and trends. The effects of climate change on the coasts of Latin America and the Caribbean. United Nations, Santiago.

Figure S1. Data quality matrix of exposure factors for the Climate Vulnerability Assessment of key fishery resources to climate change in the Northern Humboldt Current System. Exposure factor: F1) Sea surface temperature; F2) Sea surface salinity; F3) pH; F4) Sea surface chlorophyll; F5) Primary productivity; F6) Precipitation; F7) Air surface temperature; F8) Sea bottom temperature; F9) Sea bottom salinity; F10) Sea level rise. Data quality score: 3) High quality data; 2) Related data; 1) Reviewer judgement; 0) No data.

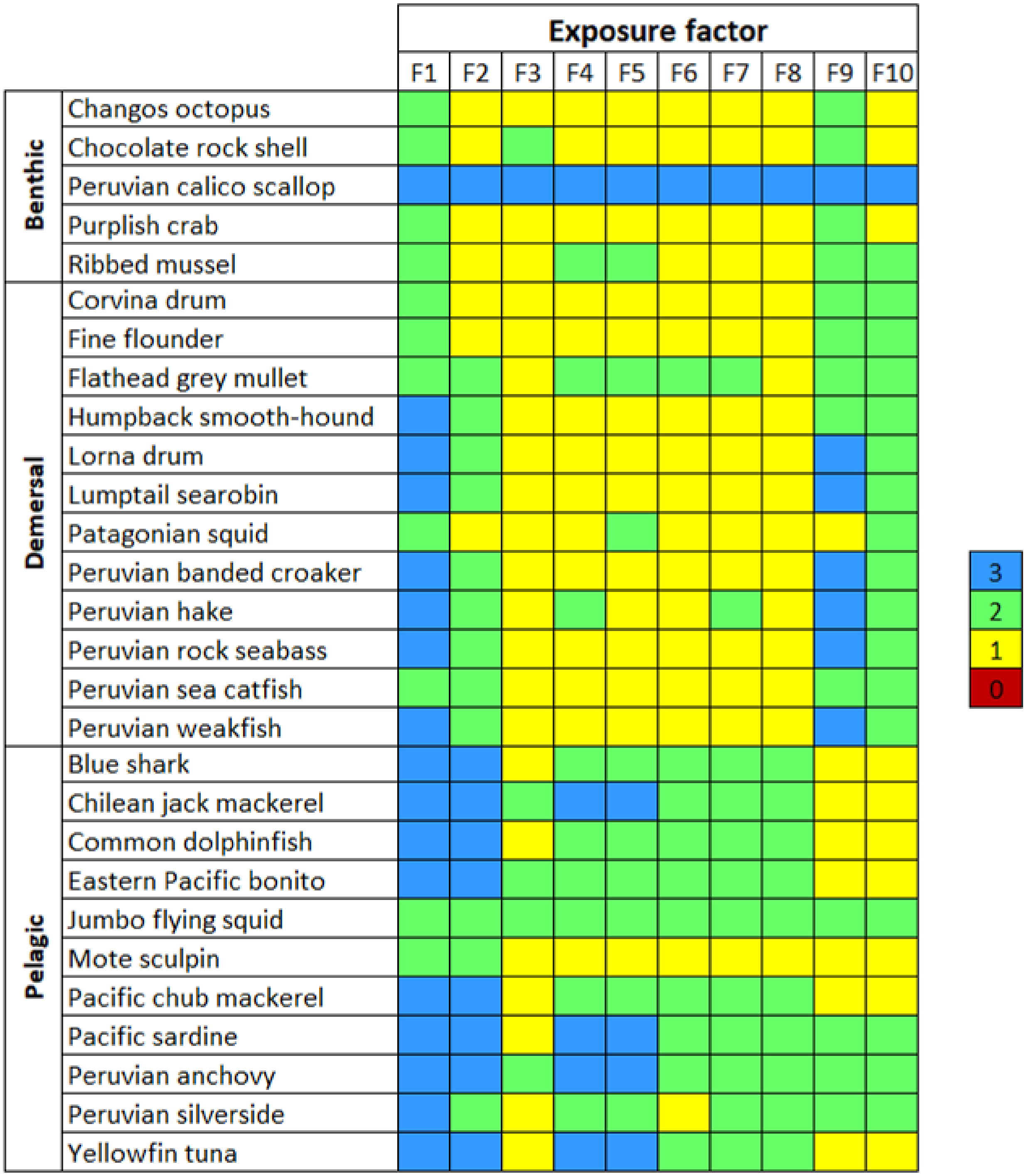

Figure S2. Data quality per group of sensitivity attributes across key fishery resources in the Northern Humboldt Current System. Data quality score: 3) High quality data; 2) Related data; 1) Reviewer judgement; 0) No data.

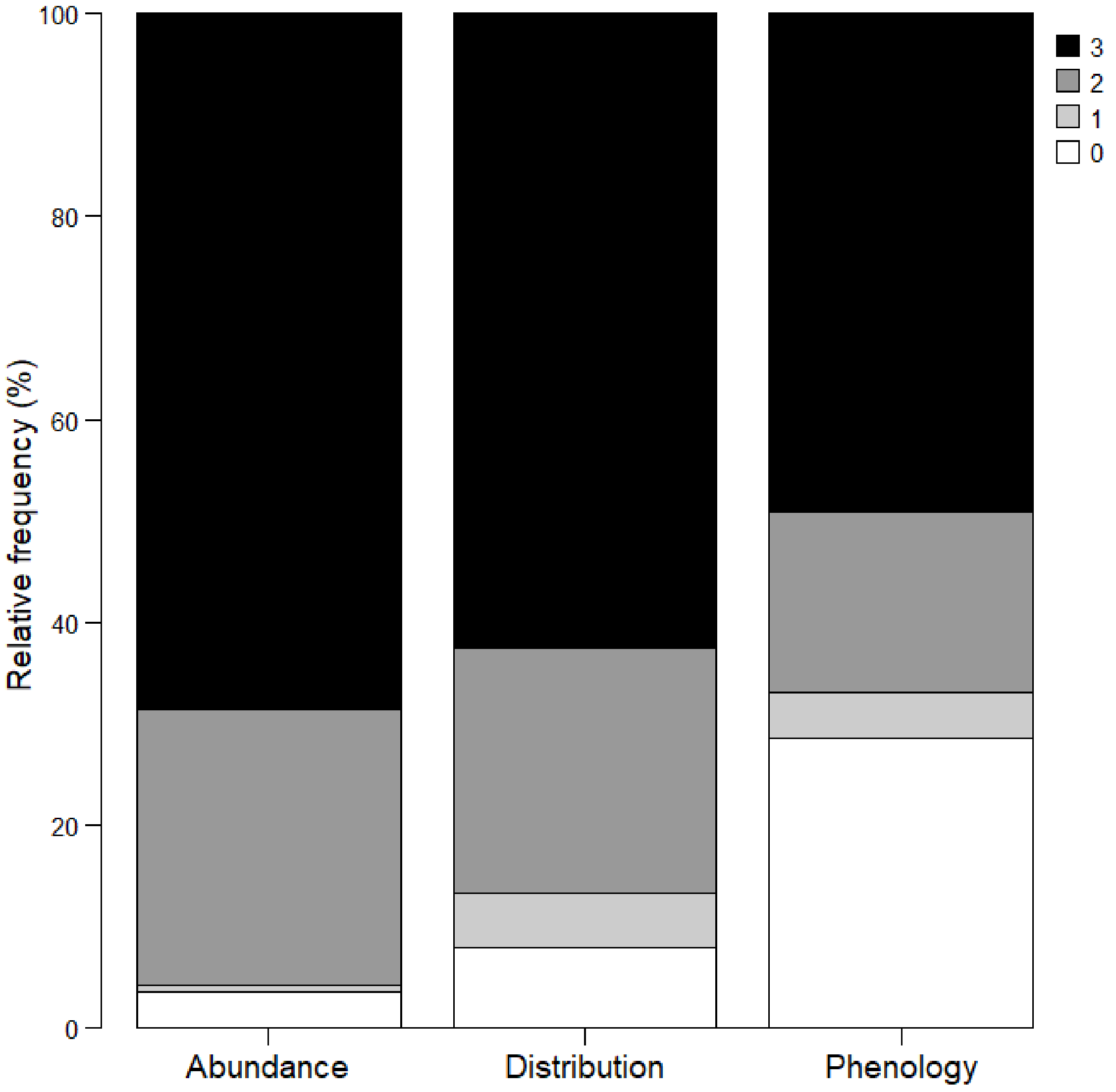

Figure S3. Data quality matrix of sensitivity atributes for the Climate Vulnerability Assessment of key fishery resources to climate change in the Northern Humboldt Current System. Sensitivity attribute: A1) Fecundity; A2) Recruitment period; A3) Average age at maturity; A4) Generalist vs. specialist; A5) Biomass; A6) Capacity for larval dispersal or larval duration; A7) Capacity for adult/juvenile movement; A8) Physiological tolerance; A9) Spatial availability of unoccupied habitat for most critical life stage; A10) Environmental variable as a phenological cue for spawning or breeding; A11) Environmental variable as a phenological cue for settlement or metamorphosis; A12) Temporal mismatches of life-cycle events; A13) Migration. Data quality score: 3) High quality data; 2) Related data; 1) Reviewer judgement; 0) No data.

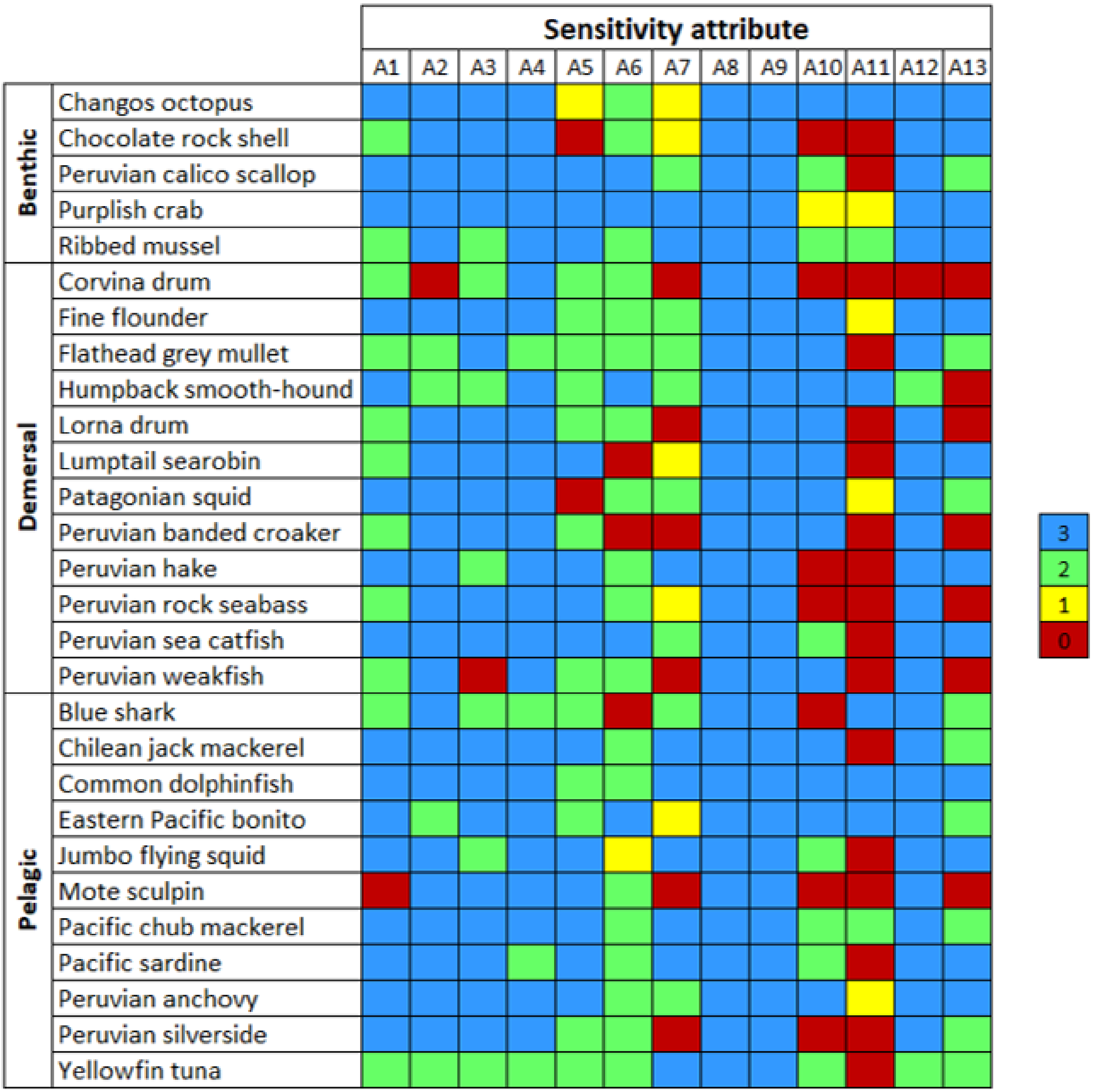

Table S1. Bootstrap outputs of certainty for exposure factors (n = 10) and sensitivity attributes (n = 13) categories (L: Low; M: Medium; H: High), and for vulnerability categories (1–2: Low; 3–4: Medium; 6: High; 9: Very high) for key fishery resources in the Northern Humboldt Current System. Certainty refers to the percentage of 10,000 iterations drawn randomly with replacement that were identical to the original distribution. The logic rule was applied after each iteration and the relative frequencies that were assigned to each bin were recorded. Certainties were classified as very high (>95%), high (91–95%), moderate (70–90%), and low (<70%).

			Exposure						Sensitivity						Vulnerability	
			Factors (n)			Certainty			Attributes (n)			Certainty			Category	Certainty
Group	Common name	Species	L	M	H	L	M	H	L	M	H	L	M	H		
Benthic	Changos octopus	*Octopus mimus*	0	8	2	0.000	0.583	0.417	4	5	4	0.000	0.004	0.996	6	0.998
Benthic	Chocolate rock shell	*Thaisella chocolata*	0	6	4	0.000	0.114	0.887	5	4	4	0.000	0.000	1.000	9	1.000
Benthic	Peruvian calico scallop	*Argopecten purpuratus*	0	6	4	0.000	0.006	0.994	6	4	3	0.000	0.000	1.000	9	1.000
Benthic	Purplish crab	*Platyxanthus orbignyi*	2	7	1	0.000	0.795	0.205	5	6	2	0.000	0.321	0.679	4	0.255
Benthic	Ribbed mussel	*Aulacomya atra*	0	5	5	0.000	0.000	1.000	4	4	5	0.000	0.000	1.000	9	1.000
Demersal	Corvina drum	*Cilus gilberti*	8	2	0	0.539	0.461	0.000	2	7	4	0.000	0.039	0.961	3	0.533
Demersal	Fine flounder	*Paralichthys adspersus*	5	5	0	0.010	0.990	0.000	2	11	0	0.000	0.996	0.004	4	0.986
Demersal	Flathead grey mullet	*Mugil cephalus*	1	9	0	0.000	1.000	0.000	3	7	3	0.000	0.412	0.588	6	0.588
Demersal	Humpback smooth-hound	*Mustelus whitneyi*	8	2	0	0.978	0.022	0.000	2	8	3	0.000	0.238	0.763	3	0.750
Demersal	Lorna drum	*Sciaena deliciosa*	5	5	0	0.015	0.985	0.000	4	7	2	0.000	0.918	0.082	4	0.907
Demersal	Lumptail searobin	*Prionotus stephanophrys*	8	2	0	0.696	0.304	0.000	3	9	1	0.000	0.995	0.005	2	0.696
Demersal	Patagonian squid	*Doryteuthis gahi*	3	4	3	0.000	0.454	0.546	4	7	2	0.000	0.799	0.201	6	0.629
Demersal	Peruvian banded croaker	*Paralonchurus peruanus*	4	6	0	0.004	0.996	0.000	2	9	2	0.000	0.925	0.075	4	0.922
Demersal	Peruvian hake	*Merluccius gayi peruanus*	8	2	0	0.840	0.160	0.000	2	10	1	0.000	0.943	0.057	2	0.795
Demersal	Peruvian rock seabass	*Paralabrax humeralis*	3	7	0	0.003	0.997	0.000	3	7	3	0.000	0.427	0.573	6	0.572
Demersal	Peruvian sea catfish	*Galeichthys peruvianus*	8	2	0	0.444	0.556	0.000	1	9	3	0.000	0.016	0.984	3	0.439
Demersal	Peruvian weakfish	*Cynoscion analis*	6	4	0	0.012	0.988	0.000	1	10	2	0.000	0.977	0.023	4	0.965
Pelagic	Blue shark	*Prionace glauca*	2	7	1	0.000	1.000	0.000	6	5	2	0.000	0.959	0.041	4	0.959
Pelagic	Chilean jack mackerel	*Trachurus murphyi*	3	7	0	0.000	1.000	0.000	6	5	2	0.000	0.926	0.074	4	0.926
Pelagic	Common dolphinfish	*Coryphaena hippurus*	4	6	0	0.000	1.000	0.000	7	4	2	0.073	0.927	0.000	4	0.927
Pelagic	Eastern Pacific bonito	*Sarda chiliensis chiliensis*	3	7	0	0.000	0.980	0.020	3	9	1	0.000	0.999	0.001	4	0.979
Pelagic	Jumbo flying squid	*Dosidicus gigas*	3	5	2	0.000	0.954	0.046	9	4	0	0.136	0.864	0.000	4	0.827
Pelagic	Mote sculpin	*Normanichthys crockeri*	3	7	0	0.000	1.000	0.000	4	6	3	0.000	0.587	0.413	6	0.413
Pelagic	Pacific chub mackerel	*Scomber japonicus*	4	6	0	0.000	0.991	0.009	6	4	3	0.000	0.110	0.890	6	0.891
Pelagic	Pacific sardine	*Sardinops sagax*	3	5	2	0.000	0.981	0.019	4	9	0	0.000	0.998	0.002	4	0.980

Pelagic	Peruvian anchovy	*Engraulis ringens*	4	5	1	0.000	0.924	0.076	3	8	2	0.000	0.713	0.287	4	0.657
Pelagic	Peruvian silverside	*Odontesthes regia*	1	8	1	0.000	0.765	0.235	3	8	2	0.000	0.824	0.176	4	0.630
Pelagic	Yellowfin tuna	*Thunnus albacares*	5	5	0	0.000	1.000	0.000	7	2	4	0.000	0.003	0.997	6	0.997

Table S2. Frequency of exposure factors and sensitivity attributes, for key fishery resources in the Northern Humboldt Current System, per category (L: Low; M: Medium; H: High) based on the logic rule (LR), with exposure and sensitivity component scores. † Rubrics with 70% certainty after the leave-one-out analysis. * Rubrics with 77% certainty after the leave-one-out analysis. All other rubrics had 100% certainty.

Group	Common name	Scientific name	Exposure			Exposure component	Sensitivity			Sensitivity component	Vulnerability	Vulnerability (leave-one-out)
			L	M	H		L	M	H			
Benthic	Changos octopus	*Octopus mimus*	0	8	2	2	4	5	4	3	6	6
Benthic	Chocolate rock shell	*Thaisella chocolata*	0	6	4	3	5	4	4	3	9	9
Benthic	Peruvian calico scallop	*Argopecten purpuratus*	0	6	4	3	6	4	3	*3	9	6
Benthic	Purplish crab	*Platyxanthus orbignyi*	2	7	1	2	5	6	2	2	4	4
Benthic	Ribbed mussel	*Aulacomya atra*	0	5	5	3	4	4	5	3	9	9
Demersal	Corvina drum	*Cilus gilberti*	8	2	0	1	2	7	4	3	3	3
Demersal	Fine flounder	*Paralichthys adspersus*	5	5	0	2	2	11	0	2	4	4
Demersal	Flathead grey mullet	*Mugil cephalus*	1	9	0	2	3	7	3	*3	6	4
Demersal	Humpback smooth-hound	*Mustelus whitneyi*	8	2	0	1	2	8	3	*3	3	2
Demersal	Lorna drum	*Sciaena deliciosa*	5	5	0	2	4	7	2	2	4	4
Demersal	Lumptail searobin	*Prionotus stephanophrys*	8	2	0	1	3	9	1	2	2	2
Demersal	Patagonian squid	*Doryteuthis gahi*	3	4	3	†3	4	7	2	2	6	4
Demersal	Peruvian banded croaker	*Paralonchurus peruanus*	4	6	0	2	2	9	2	2	4	4
Demersal	Peruvian hake	*Merluccius gayi peruanus*	8	2	0	1	2	10	1	2	2	2
Demersal	Peruvian rock seabass	*Paralabrax humeralis*	3	7	0	2	3	7	3	*3	6	4
Demersal	Peruvian sea catfish	*Galeichtys peruvianus*	8	2	0	1	1	9	3	*3	3	2
Demersal	Peruvian weakfish	*Cynoscion analis*	6	4	0	2	1	10	2	2	4	4
Pelagic	Blue shark	*Prionace glauca*	2	7	1	2	6	5	2	2	4	4
Pelagic	Chilean jack mackerel	*Trachurus murphyi*	3	7	0	2	6	5	2	2	4	4
Pelagic	Common dolphinfish	*Coryphaena hippurus*	4	6	0	2	7	4	2	2	4	4
Pelagic	Eastern Pacific bonito	*Sarda chiliensis chiliensis*	3	7	0	2	3	9	1	2	4	4
Pelagic	Jumbo flying squid	*Dosidicus gigas*	3	5	2	2	9	4	0	2	4	4
Pelagic	Mote sculpin	*Normanichthys crockeri*	3	7	0	2	4	6	3	*3	6	4
Pelagic	Pacific chub mackerel	*Scomber japonicus*	4	6	0	2	6	4	3	*3	6	4

Pelagic	Pacific sardine	*Sardinops sagax*	3	5	2	2	4	9	0	2	4	4
Pelagic	Peruvian anchovy	*Engraulis ringens*	4	5	1	2	3	8	2	2	4	4
Pelagic	Peruvian silverside	*Odontesthes regia*	1	8	1	2	3	8	2	2	4	4
Pelagic	Yellowfin tuna	*Thunnus albacares*	5	5	0	2	7	2	4	3	6	6
